# Partial ligand exchange on CsPbI_3_ nanocrystals with alkyl phosphonium iodide improves phase stability and emission efficiency in light-emitting diodes

**DOI:** 10.1039/d6sc06271g

**Published:** 2026-10-07

**Authors:** Shence Zhang, Meenakshi Pegu, Luca Goldoni, Hossein Roshan, Umberto Filippi, Abdessamad El Adel, Francesco De Boni, Simone Lauciello, Zhanzhao Li, Yuting Song, Ivan Infante, Francesco Di Stasio, Liberato Manna

**Affiliations:** a Nanochemistry, Istituto Italiano di Tecnologia Via Morego 30 16163 Genova Italy liberato.manna@iit.it; b Department of Chemistry, Materials and Chemical Engineering, Politecnico di Milano Piazza Leonardo da Vinci 32 20133 Milano Italy; c Materials Characterization Facility, Istituto Italiano di Tecnologia Via Morego 30 16163 Genova Italy; d Electron Microscopy Facility, Istituto Italiano di Tecnologia Via Morego 30 16163 Genova Italy; e School of Materials Science and Engineering, Shanghai University Shangda Road 99 200444 Shanghai China; f BCMaterials, Basque Center for Materials, Applications, and Nanostructures Leioa 48940 Spain; g Ikerbasque Basque Foundation for Science Bilbao 48009 Spain; h Photonic Nanomaterials, Istituto Italiano di Tecnologia Via Morego 30 16163 Genova Italy

## Abstract

All-inorganic colloidal CsPbI_3_ nanocrystals are promising materials for light-emitting applications owing to their excellent optoelectronic properties. However, their practical implementation remains limited by surface defects, ligand instability, and phase degradation under ambient conditions. Here, we employ trimethyl(dodecyl)phosphonium iodide (TDP-I) as a post-synthetic ligand for partial ligand exchange on CsPbI_3_ nanocrystals initially coated with a ligand shell that is primarily composed of oleylammonium ions. X-ray photoelectron spectroscopy, Fourier-transform infrared spectroscopy, and nuclear magnetic resonance analyses confirm that TDP-I is only partially replacing the native ligand shell. These findings are in line with classical molecular dynamics calculations, which indicate that a mixed oleylammonium/TDP^+^ ligand shell is more stable than a full TDP^+^ one. The TDP-I treatment improved photophysical properties including an increase in the photoluminescence quantum yield (from ∼77% to ∼90%), accompanied by prolonged photoluminescence lifetime and reduced nonradiative recombination. The TDP-I-treated nanocrystals also exhibit improved stability, with delayed transformation from the orthorhombic phase to the non-perovskite δ-phase under ambient conditions. When incorporated into light-emitting diodes, the TDP-I-treated CsPbI_3_ nanocrystals deliver a maximum external quantum efficiency of 12.4%. These results demonstrate that partial phosphonium ligand exchange provides an effective strategy for optimizing the surface chemistry, optical properties, and device performance of CsPbI_3_ nanocrystals, highlighting the importance of mixed-ligand surface engineering for perovskite optoelectronics.

## Abstract 2

Partial ligand exchange on CsPbI_3_ nanocrystals with trimethyl(dodecyl)phosphonium iodide improves their photoluminescence quantum yield and stability. Light emitting diodes incorporating such nanocrystals reach a 12.4% external quantum efficiency.

## Graphical abstract

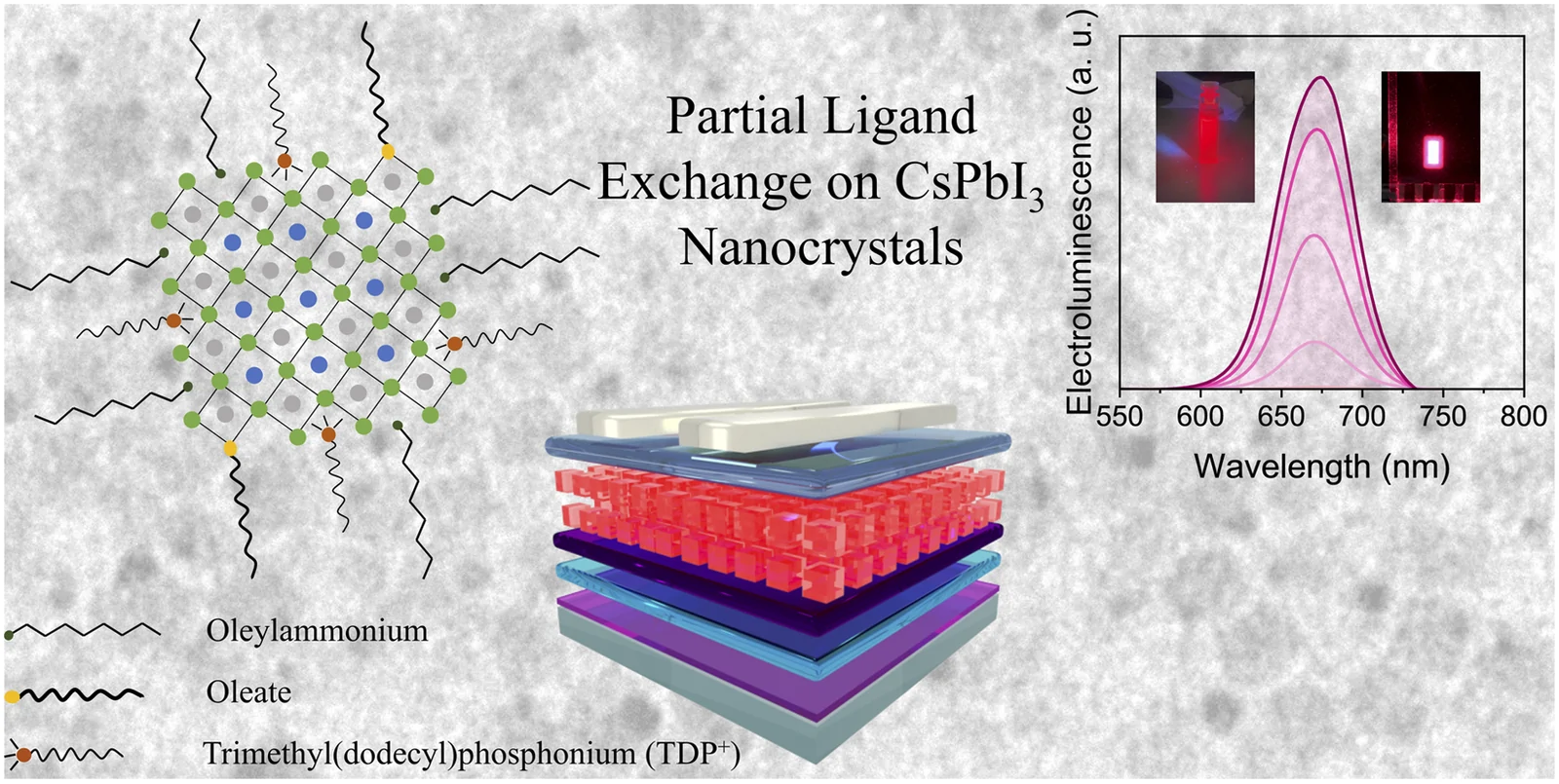

## Introduction

Lead halide perovskite nanocrystals (NCs) have emerged as promising materials for next-generation optoelectronic applications, including light emitting diodes (LEDs), scintillators, luminescent solar concentrators, photodetectors, and solar cells.^1–4^ The interest in these materials arises from their appealing optoelectronic properties, such as high photoluminescence quantum yield (PLQY), narrow emission linewidths, and tunable bandgaps spanning the visible to near-infrared spectral regions.^5–7^ In addition, their solution-processability and defect-tolerant electronic structure make them attractive for low-cost, high-performance optoelectronic applications.^8^ Among them, all-inorganic caesium lead halide perovskite NCs (CsPbX_3_, X = Cl, Br, I, or mixed halides) have drawn particular attention.^9–11^ CsPbI_3_ NCs, for example, are highly attractive for red-emitting and photovoltaic applications owing to their narrow bandgap and strong absorption across the visible spectrum.^12,13^ However, their practical implementation is limited by the intrinsic surface and phase instability under ambient conditions.^14,15^ One main issue is represented by the native surface ligands, which might detach from the surface of NCs, adversely affecting both PLQY and material stability.^11,16^ The synthesis of CsPbI_3_ NCs typically employs oleic acid (OA) and oleylamine (OLAm), leading to a ligand shell that is composed of oleate and oleylammonium ions.^17,18^ Over time, these ligands can acquire (oleate) or lose (oleylammonium) a proton, hence becoming neutrally charged and thus can get detached from the surface of the NCs, resulting in colloidal instability and formation of trap states, as discussed previously. Furthermore, these long-chain ligands create an electrically insulating barrier around the NC surface.^19^ Consequently, the ligand shell limits interparticle electronic coupling and hinders charge injection and transport across device interfaces, leading to low performance of NC-based LEDs.^20–22^ Hence, the design of a more stable ligand shell delivering defect passivation while ensuring more favourable interparticle electronic coupling is essential for improving the optoelectronic performance of CsPbI_3_ NCs.^19,23–25^

Especially to address the surface stability challenge a wide range of ligands, including alkyl quaternary ammonium halides, thiols, sulfonium halides, phosphine oxides, and zwitterionic molecules have been explored for surface passivation, either through *in situ* synthesis or by post-synthetic treatments.^26–29^ The use of quaternary ammonium ions has been shown to improve both stability and PLQY in perovskite NCs.^26,31^ On the other hand, it is reasonable to expect that quaternary ammonium ions with too many bulky alkyl chains (for example dimethyl-didodecyl ammonium ions) can hinder charge-carrier transport in NC films. These limitations highlight the need for alternative ligand chemistries that provide effective surface passivation while reducing steric hindrance and so improve electronic coupling.^30,31^

In this context, phosphonium-based organic ligands have recently emerged as a promising class of surface modifiers for perovskite NCs, demonstrating effective surface passivation together with improved colloidal stability.^25,32,33^ Recently, aromatic phosphonium halide ion pairs, when used to passivate CsPbX_3_ NC films, were found to facilitate charge transport and injection, and overall improve the efficiency of perovskite LEDs.^25,32,33^ Also, in our previous report we demonstrated that a post-synthesis ligand exchange on CsPbBr_3_ NCs with trimethyl(tetradecyl)phosphonium bromide (TTP-Br) results in long-term stability, excellent optical properties and preservation of the morphology of the NCs. Both experimental characterization and density functional theory (DFT) calculations revealed that phosphonium ligands exhibited binding geometries and adsorption strengths similar to those of didodecyldimethylammonium (DDA). Notably, unlike DDA, which contains two long alkyl chains, TTP^+^ possesses only a single long alkyl chain. This reduces steric hindrance and indeed TTP^+^ improved charge transport and carrier injection in NC films, ultimately leading to better LED performance.^25^

To date, the use and role of phosphonium ligands in regulating the surface chemistry of iodide-based perovskite NCs has remained underexplored, hence the motivation in the present work is to extend this type of ligand exchange strategy to CsPbI_3_ NCs. In this case, we select trimethyl(dodecyl)phosphonium iodide (TDP-I) as a ligand pair with which to treat the CsPbI_3_ NCs. We synthesize TDP-I *via* alkylation of trimethylphosphine and then employ it in the post-synthetic ligand-exchange on CsPbI_3_ NCs, which are initially coated with a mixed oleylammonium/oleate ligand shell. Contrary to our previous findings on CsPbBr_3_ NCs,^25^ in the present case we can only achieve a limited replacement of the native ligand shell, as assessed by various techniques, including nuclear magnetic resonance (NMR) analysis (Scheme 1). Still, the TDP-I-treated CsPbI_3_ NCs exhibit an enhancement in optical performance, with PLQY increasing from ∼77% for the pristine CsPbI_3_ NCs to ∼90% after ligand exchange, as well as improved stability over time.

**Scheme 1:**
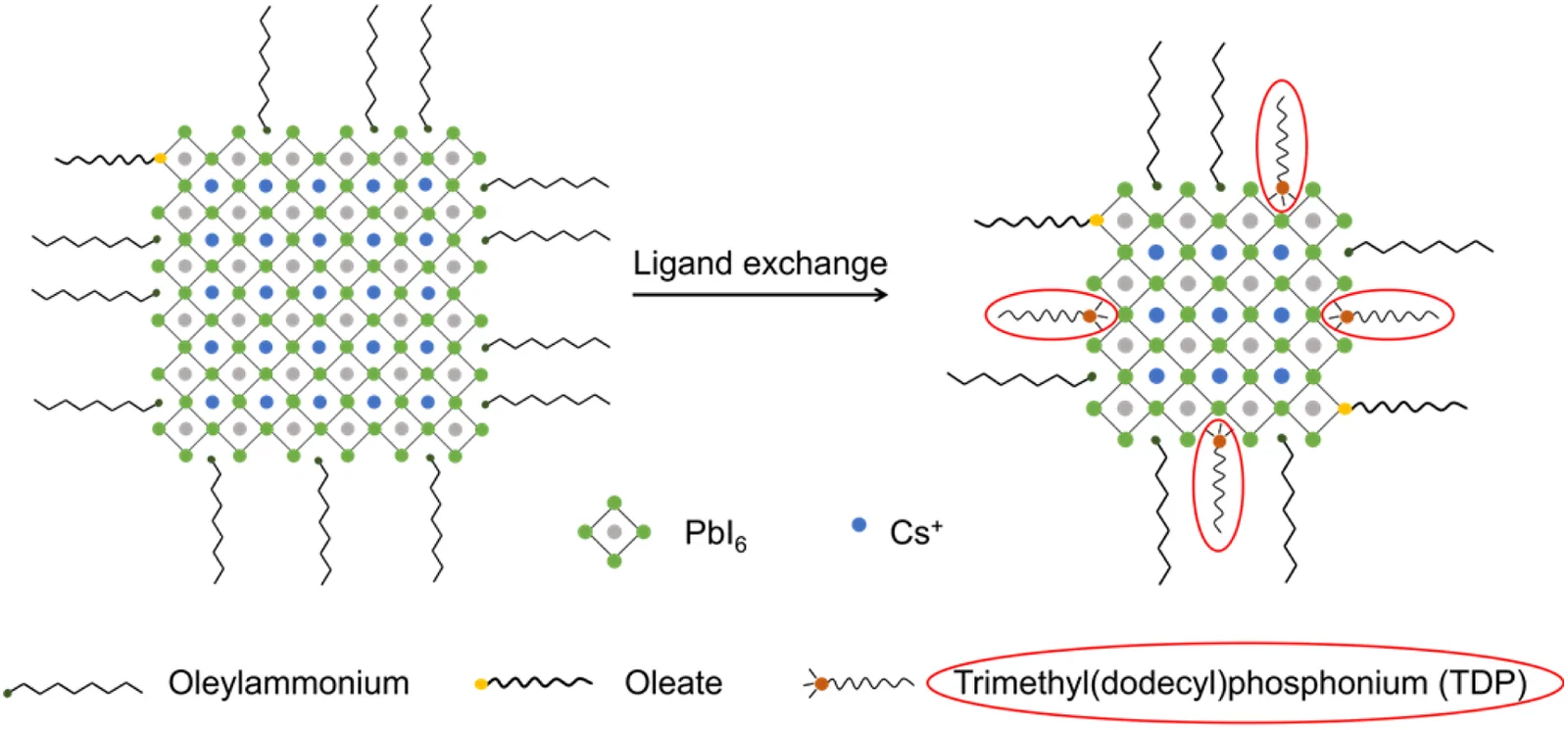
Schematic illustration of the post-synthesis ligand exchange involving Cs-oleate- and oleylammonium-capped CsPbI_3_ NCs with TDP-I.

We run classical molecular dynamics (MD) simulations, which reveal that a fully TDP^+^ passivated NC surface is much less stable (in terms of root-mean-square deviation of the inorganic core atoms of the NC) than a fully oleylammonium one or a mixed TDP^+^/oleylammonium one. According to MD, the instability is mainly related to the iodine atoms. This outcome is in line with the strong binding affinity of the phosphonium ion (a soft cation) with the iodide ion (a soft anion). It is also in line with our experimental findings that the use of increasing amounts of TDP-I during the ligand exchange procedure leads to progressive etching of the NCs, most likely initiated by extraction of I^−^ ions from the surface of the NCs by the phosphonium ions. Finally, we fabricate LEDs based on the TDP-I-treated NCs and achieve a maximum EQE of 12.4%.

Together, these results demonstrate that controlled partial exchange with quaternary phosphonium ligands provides an effective strategy to optimize the surface chemistry and optoelectronic performance of CsPbI_3_ NCs.

## Results and discussion

TDP-I was synthesized following our previously reported one-pot procedure under an inert nitrogen atmosphere.^25,34^ The synthesis involved a nucleophilic substitution reaction between trimethylphosphine (PMe_3_) and dodecyl iodide that led to the corresponding phosphonium iodide salt. Briefly, dodecyl iodide (5.5 mmol) was added to a three-neck round-bottom flask containing 10 mL of anhydrous toluene and degassed for 30 min. Subsequently, 5 mL of a 1.0 M trimethylphosphine (PMe_3_) solution in toluene was introduced, and the reaction mixture was refluxed under nitrogen to yield the desired product (Scheme S1). The chemical structure of the purified salt was confirmed by ^1^H, ^1^H-^13^HSQC (heteronuclear single quantum coherence) and HSQC ^13^C NMR spectroscopy (Fig. S1–S3, the ligand spectra with assignment). Detailed synthetic procedures and characterization data are provided in the SI. The purified TDP-I was subsequently employed for the surface treatment of the CsPbI_3_ NCs.

The pristine CsPbI_3_ NCs were synthesized *via* a modified trimethylsilyl iodide (TMS-I) hot-injection procedure at 150 °C following previously reported recipes (see the SI for details).^20^ Oleic acid and oleylamine were employed in the synthesis. For the TDP-I treatment, soon after quenching the synthesis, predetermined volumes of a 25 mM TDP-I solution (0.5, 0.75 mL, and 1 mL) in a chloroform/toluene mixture (10% v/v) were added to the crude CsPbI_3_ NC solution under a nitrogen atmosphere. The mixture was stirred for 20 min to facilitate the interaction between TDP-I and the NCs' surface, followed by precipitation with ethyl acetate, after which the NCs were collected by centrifugation and then redispersed in anhydrous toluene for further characterization (Fig. S4). The resulting samples are denoted according to the volume of TDP-I solution employed during the treatment (0.5 mL, 0.75 mL, and 1 mL), while the untreated OA/OLAm-capped CsPbI_3_ NCs are referred to as the 0 mL sample and served as the pristine sample throughout this study.

The morphology of the CsPbI_3_ NCs before and after the TDP-I treatment was investigated by transmission electron microscopy (TEM). The pristine CsPbI_3_ NCs had a uniform cubic morphology with an average size of 11.8 ± 1.6 nm (Fig. 1a). With increasing amounts of TDP-I added, the NCs progressively lost their cubic shape (Fig. 1a and S5), evolving into more irregular shapes, and decreased in size to 4.6 ± 1.2 nm, pointing to unselective ligand-induced surface etching. This is different from the facet-selective etching reported for phosphonate-treated CsPbBr_3_ NCs.^35,36^ Also, this behaviour is consistent with our previous observations on the TTP-Br-treated CsPbBr_3_ NCs,^25^ although the effect appears more pronounced in the CsPbI_3_ NCs of this work. The crystal structure of the CsPbI_3_ NCs before and after TDP-I treatment was examined by X-ray diffraction (XRD) analysis. As reported in Fig. 1b, the diffraction patterns of the pristine (0 mL), 0.5 mL, and 0.75 mL treated samples were consistent with the orthorhombic γ-CsPbI_3_ phase (ICSD No. 264725), indicating that the TDP-I treatment preserves the perovskite crystal structure. The pristine NCs (*i.e.*, 0 mL) exhibited two dominant diffraction peaks at 14.1° and 28.6°, due to preferential orientation of the cubic NCs. Upon TDP-I treatment, additional peaks corresponding to the (110), (112), (220), (202), (310), and (224) planes became visible, consistent with the orthorhombic γ-CsPbI_3_ phase. The higher number of observable reflections is attributed to the partial disruption of the cubic shape induced by the ligand treatment, which led to a lower degree of preferential orientation. The treatment with 1 mL of TDP-I (Fig. S6) led to the appearance of diffraction features associated with the non-perovskite δ-phase, indicating the onset of structural degradation. Therefore, only the 0.5 and 0.75 mL TDP-I-treated samples are considered in the follow-up experiments.

**Fig. 1:**
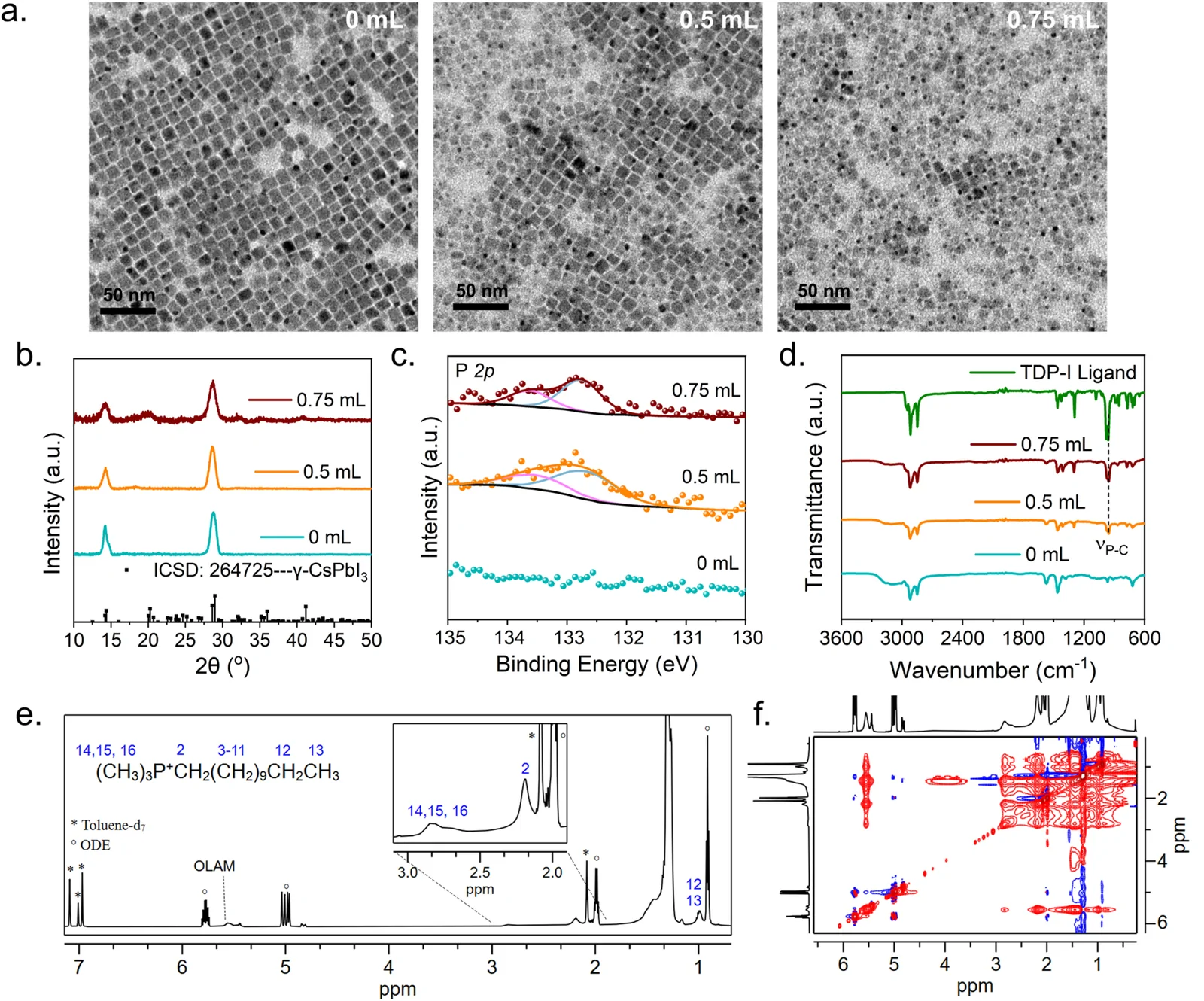
(a) TEM micrographs of pristine (0 mL), 0.5 mL TDP-I-treated, and 0.75 mL TDP-I-treated CsPbI_3_ NCs. (b) X-ray diffraction (XRD) patterns of pristine and TDP-I-treated CsPbI_3_ NCs together with the reference pattern of orthorhombic γ-CsPbI_3_ (ICSD No. 264725). (c) XPS spectra of the P 2p region for pristine and TDP-I-treated CsPbI_3_ NCs. (d) FTIR spectra of the TDP-I ligand, pristine, and TDP-I-treated CsPbI_3_ NCs. (e) ^1^H NMR spectrum of TDP-I treated CsPbI_3_ NCs in toluene-d8 with peak assignment. (f) 2D ^1^H–^1^H NOESY spectrum of TDP-I treated CsPbI_3_ NCs in toluene-d8.

The structural stability of the CsPbI_3_ NCs was further assessed by monitoring their XRD patterns during storage under air (Fig. S7–S9). After one week, the 0.5 mL and 0.75 mL TDP-I-treated samples largely retained the diffraction features of orthorhombic γ-CsPbI_3_, whereas the pristine (0 mL) sample exhibited more pronounced signatures of phase degradation. After two weeks, the reflections associated with the non-perovskite δ-phase became increasingly evident in all samples. These observations indicate that the TDP-I treatment delays the γ-to-δ phase transition, thereby enhancing the structural stability of CsPbI_3_ NCs during aging under ambient conditions.

We studied the various samples with X-ray photoelectron spectroscopy (XPS). The measurements were performed on the pristine (0 mL) and TDP-I-treated samples (0.5 mL and 0.75 mL). As shown in Fig. 1c, distinct P 2p peaks emerged at 132.8 eV and 133.6 eV after the TDP-I treatment, while no detectable P 2p signal was observed for the pristine CsPbI_3_ NCs (see also Fig. S10–S13, and Table S1–S2 in the SI). Scanning electron microscopy (SEM) energy-dispersive X-ray spectroscopy (EDX) evidenced a homogeneous distribution of phosphorus across the CsPbI_3_ NC film (Fig. S14–S16 and Tables S3–S5). We also carried out Fourier-transform infrared (FTIR) analysis. Compared to the untreated sample, the TDP-I-treated NCs exhibited a progressive and distinct peak at ∼950 cm^−1^, assigned to the P–C stretching vibration of the PMe_3_ group, confirming the presence of the TDP^+^ species.

The ligand-shell reorganization upon TDP-I treatment was probed by ^1^H NMR spectroscopy (Fig. 1e). The ^1^H NMR spectrum of the TDP-I treated NCs evidenced the presence of a peak at 5.6 ppm stemming from the protons of the double bond in the OA/OLAm ligands. A signal corresponding to the PMe_3_^+^ group was also visible, as broadened features, in the range from 2.1 ppm to 3.0 ppm, corroborating the binding of TDP^+^ to the NC surface. The interaction of the TDP^+^ ions with the CsPbI_3_ NCs' surface was further investigated by ^1^H–^1^H NOESY spectroscopy, acquired in toluene-d8 (Fig. 1f). The spectrum displayed negative (red) NOE cross-peaks, which are characteristic of species possessing long rotational correlation times (*τ*_c_). The negative NOE cross-peaks indicate that the TDP^+^ molecules adopt the slow-tumbling dynamic regime of the NCs upon binding the CsPbI_3_ NC surface (Fig. 1f). In contrast, free ligands typically exhibit positive (blue) NOE cross-peaks owing to their much shorter *τ*_c_ values. The complete NMR characterization of TDP-I-capped CsPbI_3_ NCs in toluene-d8 and ligand structure formula with peak assignment are reported in the SI (Fig. S17–S19).

The ligand-shell composition was determined by combining quantitative ^1^H NMR (q-NMR) analysis (Fig. S20) with compositional analysis of the purified CsPbI_3_ NCs using inductively coupled plasma optical emission spectroscopy (ICP-OES). The pristine NCs were predominantly passivated by oleylammonium ligands (93%), with a minor fraction of oleate ligands (7%). After the treatment with 0.75 mL TDP-I, the native ligand shell undergoes partial reorganization to yield a mixed ligand shell composed of 54% oleylammonium, 14% oleate, and 32% TDP^+^ ions (Table S6).

We investigated the influence of the TDP-I treatment on the optical properties of the CsPbI_3_ NCs. The TDP-I-treated samples exhibited blue-shifted excitonic absorption peaks compared to the pristine (0 mL) CsPbI_3_ NCs (Fig. 2a). The PL peak position, full width at half-maximum (FWHM), PL quantum yield (PLQY), and PL lifetime of the NCs in solution and film are summarized in Table 1. The PL emission progressively blue-shifted from 678 nm for the pristine (0 mL) CsPbI_3_ NCs to 674 and 668 nm for the 0.5 mL and 0.75 mL TDP-I treated samples (Fig. 2a). For the 1 mL TDP-I-treated sample, the peak further blue-shifted to 627 nm (Fig. S21 and S22). The blue shifts in both absorption and emission spectra are consistent with the reduction in NC size observed under TEM. In terms of PLQY, we observe an increment from 77 ± 8% for the pristine (0 mL) CsPbI_3_ NCs to a maximum of 89 ± 9% for the 0.5 mL and 86 ± 9% for the 0.75 mL TDP-I-treated samples. Further increasing the TDP-I treatment to 1 mL resulted in a decrease in PLQY to 75 ± 7%, suggesting that an excess of TDP-I leads to NC degradation and reduced optical performance. A similar trend was observed in thin films (Fig. 2b and S23). The PL emission maximum shifted from 680 nm for the pristine (0 mL) film to 675 nm and 671 nm for the 0.5 mL and 0.75 mL TDP-I-treated films, respectively. The close correspondence between the solution and film PL spectra further confirms that the TDP-I treatment effectively preserves their emissive characteristics in the film, which is desirable for fabricating LEDs with enhanced performance.

**Fig. 2:**
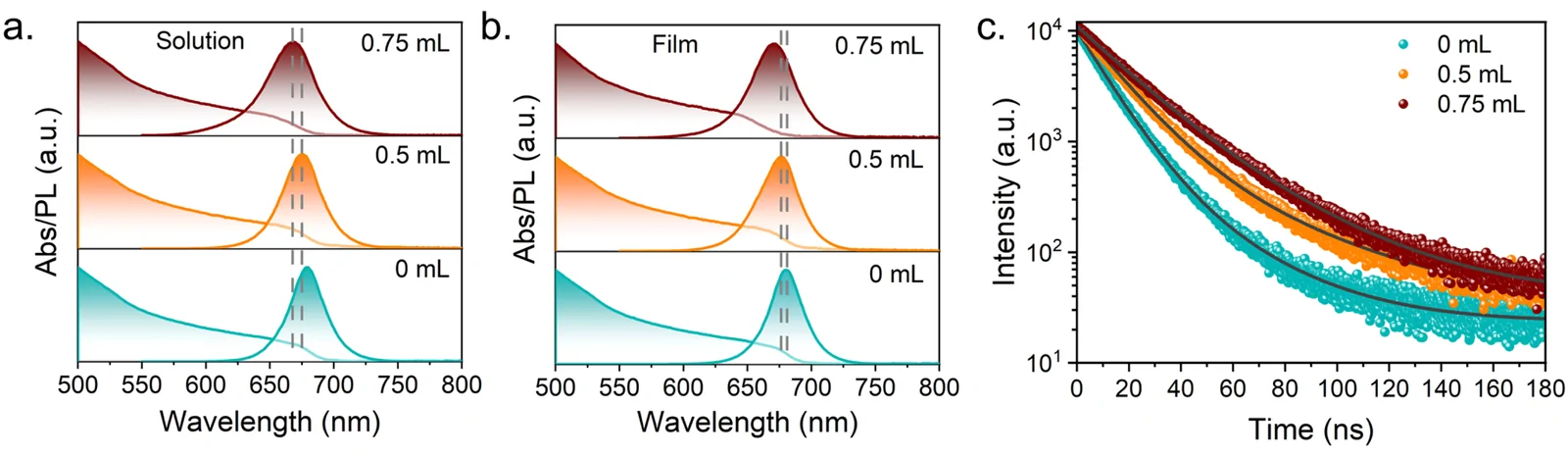
(a) UV-visible absorption and PL spectra of 0 mL, 0.5 mL and 0.75 mL TDP-I-treated CsPbI_3_ NCs dispersed in anhydrous toluene. (b) UV-visible absorption and PL spectra of 0 mL, 0.5 mL and 0.75 mL TDP-I-treated CsPbI_3_ NCs in thin film. (c) PL decay profile of 0 mL, 0.5 mL and 0.75 mL TDP-I-treated CsPbI_3_ NCs in anhydrous toluene.

**Table 1:** Optical properties of pristine and TDP-I treated CsPbI_3_ NCs in solution and thin film

Samples	Solution				Thin film		
	PL peak (nm)	FWHM, PL (nm)	PLQY (%)	*τ*_avg_ (ns)	PL peak (nm)	FWHM, PL (nm)	PLQY (%)
0 mL	678	36	77 ± 8	16.9	680	35	41 ± 4
0.5 mL	674	39	89 ± 9	24.3	675	41	49 ± 5
0.75 mL	668	47	86 ± 9	26.4	671	44	47 ± 5

Time-resolved PL measurements revealed that the PL lifetime increases after the TDP-I treatment, consistent with the observed enhancement in PLQY. The PL decay profiles are fitted with bi-exponential functions. The average lifetime (*τ*_avg_) increased from 16.9 ns for the pristine (0 mL) CsPbI_3_ NCs to 24.3 ns (0.5 mL) and 26.4 ns (0.75 mL) for the TDP-I-treated samples, respectively (Fig. 2c and Table S7). The prolonged PL lifetime combined with the increased PLQY indicate reduced nonradiative recombination, induced by surface defect passivation and suppressed trap-assisted recombination operated by TDP-I. The stability of the CsPbI_3_ NCs was assessed by monitoring their PLQY during storage under ambient conditions for a period of six weeks (Fig. S24). Compared to the pristine (0 mL) sample, both TDP-I-treated samples (0.5 mL and 0.75 mL) exhibited significantly improved PLQY retention over time. Notably, the 0.75 mL-treated sample maintained the highest PLQY (∼80%) for up to four weeks.

To gain atomistic insight into the TEM-observed TDP induced morphology changes, classical molecular dynamics (MD) simulations were performed in toluene over 50 ns for CsPbI_3_ NCs capped with either oleylammonium or TDP^+^ ligands at high and low surface densities (see SI for computational details). To ensure high fidelity, the force field parameters were initially tailored to describe each pure ligand-surface system independently (Fig. S25 and Table S8). The mixed-ligand surface was subsequently simulated by applying standard combination rules to cross-interactions. While this approach does not constitute a direct parameterization of the multi-component shell, it provides a reliable approximation of the blended interface. The root-mean-square deviation (RMSD) of the inorganic core atoms, calculated with respect to the initial configuration, was used to monitor the structural evolution of each system throughout the trajectory (Fig. 3a).

**Fig. 3:**
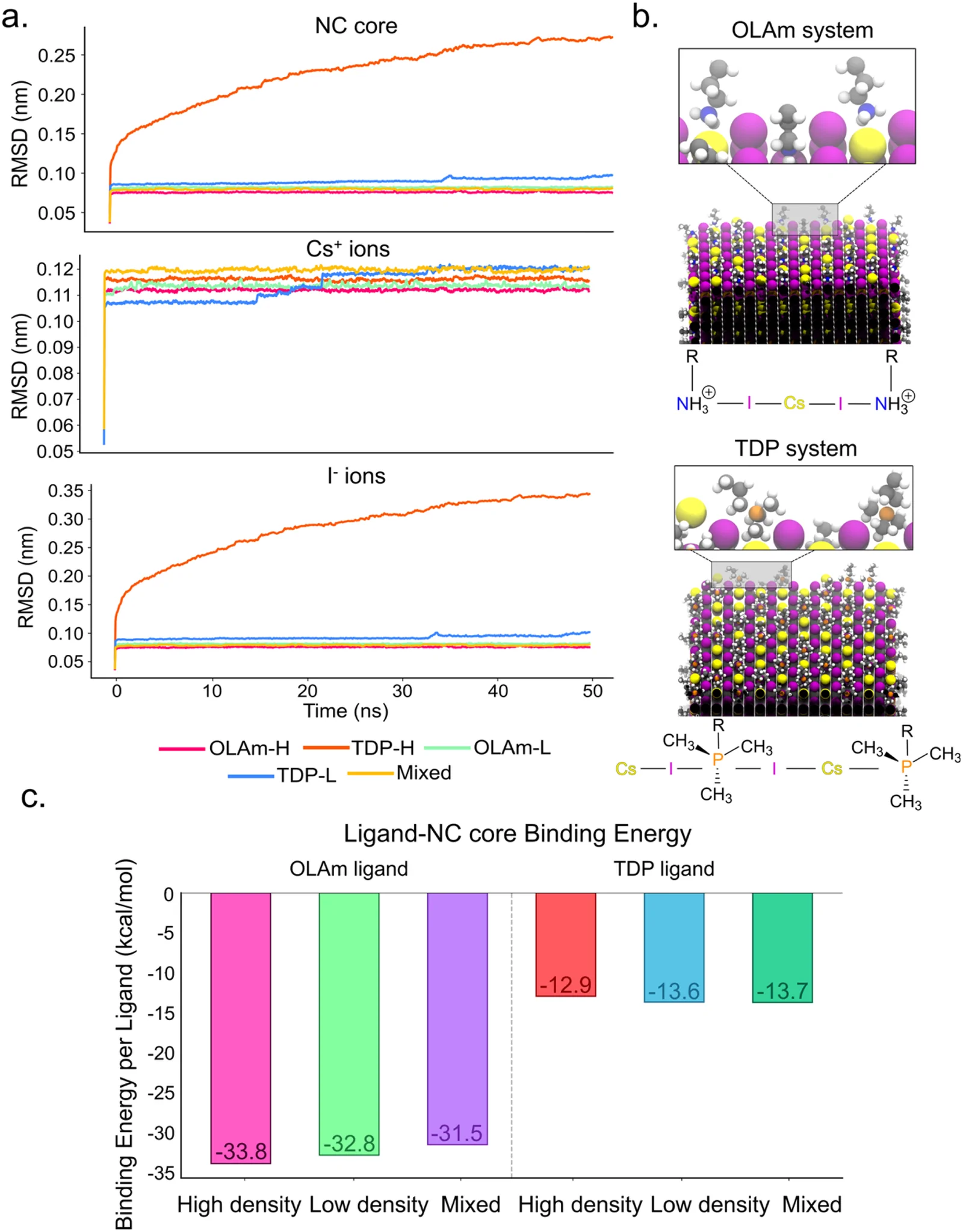
(a) Time evolution of the root-mean-square deviation (RMSD) of the NC inorganic core atoms, Cs^+^ ions and I^−^ ions over the 50 ns MD trajectory. (b) Representative binding motifs of OLAm (top) and TDP (bottom) on the CsPbI_3_ surface. (c) Per-ligand ligand–NC core binding energies extracted from energy-group decomposition for OLAm and TDP ligands in high-density, low-density, and mixed OLAm/TDP-capped NC systems. The “mixed” bars correspond to the OLAm and TDP ligands within the same mixed-ligand NC model.

In the high-density case, the OLAm-capped NCs remained highly stable (average RMSD of 0.073 nm), whereas the TDP-capped counterpart displayed a progressively increasing core RMSD, indicative of continuous lattice reorganization. To isolate the driver of this instability without the interference of highly mobile organic tail conformations, the RMSDs of the Cs^+^ and I^−^ ions were tracked independently (Fig. 3a). While the Cs^+^ sublattice remained rigid across all models, the I^−^ RMSD of the high-density TDP-capped NCs increased continuously throughout the trajectory. This selective disorder tracks the displacement of surface iodide ions, signalling the detachment of phosphonium–iodide ion pairs and directly supporting the experimentally observed surface etching at higher phosphonium iodide concentrations to ligand exchange the native OLAm ligand. Crucially, lowering the TDP^+^ density to 1.1 ligand per nm^2^ or simulating the mixed-ligand surface restored full structural stability, yielding core and iodide RMSD profiles indistinguishable from the pristine OLAm references.

To quantify these surface affinities, the per-ligand potential interaction energy between the organic ligand headgroup and the NC core was extracted *via* snapshot-based energy-group decomposition (Fig. 3c). Since these values bypass the configuration sampling of desorbed states, they represent a localized enthalpic interaction rather than a true thermodynamic binding free energy (Δ*G*). OLAm exhibited consistently stronger enthalpic binding across all coverages (−31.5 to −33.8 kcal mol^−1^), while the direct interaction of the TDP cation with the core was significantly weaker but uniform (−12.9 to −13.7 kcal mol^−1^). The preservation of these distinct energetic signatures within the mixed-ligand simulation confirms that each species retains its individual coordination character. The difference in binding energies for TDP^+^ and OLAm appears to be incompatible with the experimentally measured surface ligand composition assessed from NMR. However, one should note that during the ligand-exchange process TDP-I is added in large excess relative to the available surface binding sites on the CsPbI_3_ NCs. The resulting high concentration (hence high chemical potential) of TDP-I in solution provides a strong thermodynamic driving force for partial exchange of OLAm with TDP^+^ according to the mass-action principle, despite its weaker intrinsic binding affinity compared to OLAm.

These results collectively demonstrate that the destabilization at high TDP^+^ coverage arises primarily from excessive ligand packing density and the associated tendency to form phosphonium–iodide pairs that are prone to detach from the surface rather than from an intrinsic incompatibility between TDP^+^ and the CsPbI_3_ NCs surface, and that the partial ligand-exchange strategy employed experimentally strikes an optimal balance between strong OLAm anchoring and complementary TDP^+^ surface passivation, consistent with the enhanced PLQY retention and delayed phase transformation observed experimentally for the partially exchanged NCs.

To assess the impact of the TDP-I treatment on device operation, the treated CsPbI_3_ NCs were incorporated into LEDs based on the following architecture: indium tin oxide (ITO)/poly(2,3-dihydrothieno-1,4-dioxin)–poly(styrenesulfonate) (PEDOT:PSS)/poly(4-butylphenyldiphenylamine) (Poly-TPD)/0.75 mL TDP-I-treated CsPbI_3_ NCs/1,3,5-tris(1-phenyl-1*H*-benzimidazol-2-yl)benzene (TPBi)/lithium fluoride (LiF)/Al, where PEDOT:PSS/Poly-TPD and TPBi serve as the hole transport and electron transport layers, respectively. A representative schematic and cross-sectional SEM image of the device is shown in Fig. 4a. The combined thickness of the PEDOT:PSS and Poly-TPD layers was approximately 30 nm, while the CsPbI_3_ NC, TPBi, and LiF/Al layers had thicknesses of approximately 20, 30, and 100 nm, respectively. The corresponding flat-band energy diagram is illustrated in Fig. 4b.^7,25^ Ultraviolet photoelectron spectroscopy (UPS) combined with optical absorption measurements were employed to determine the energy levels of CsPbI_3_ NC films before and after 0.75 mL TDP-I treatment (Fig. S26–S28 and Table S9). Both control and TDP-I-treated films exhibited a valence band maximum of approximately –5.6 eV, consistent with previously reported values for CsPbI_3_ NCs.^37^

**Fig. 4:**
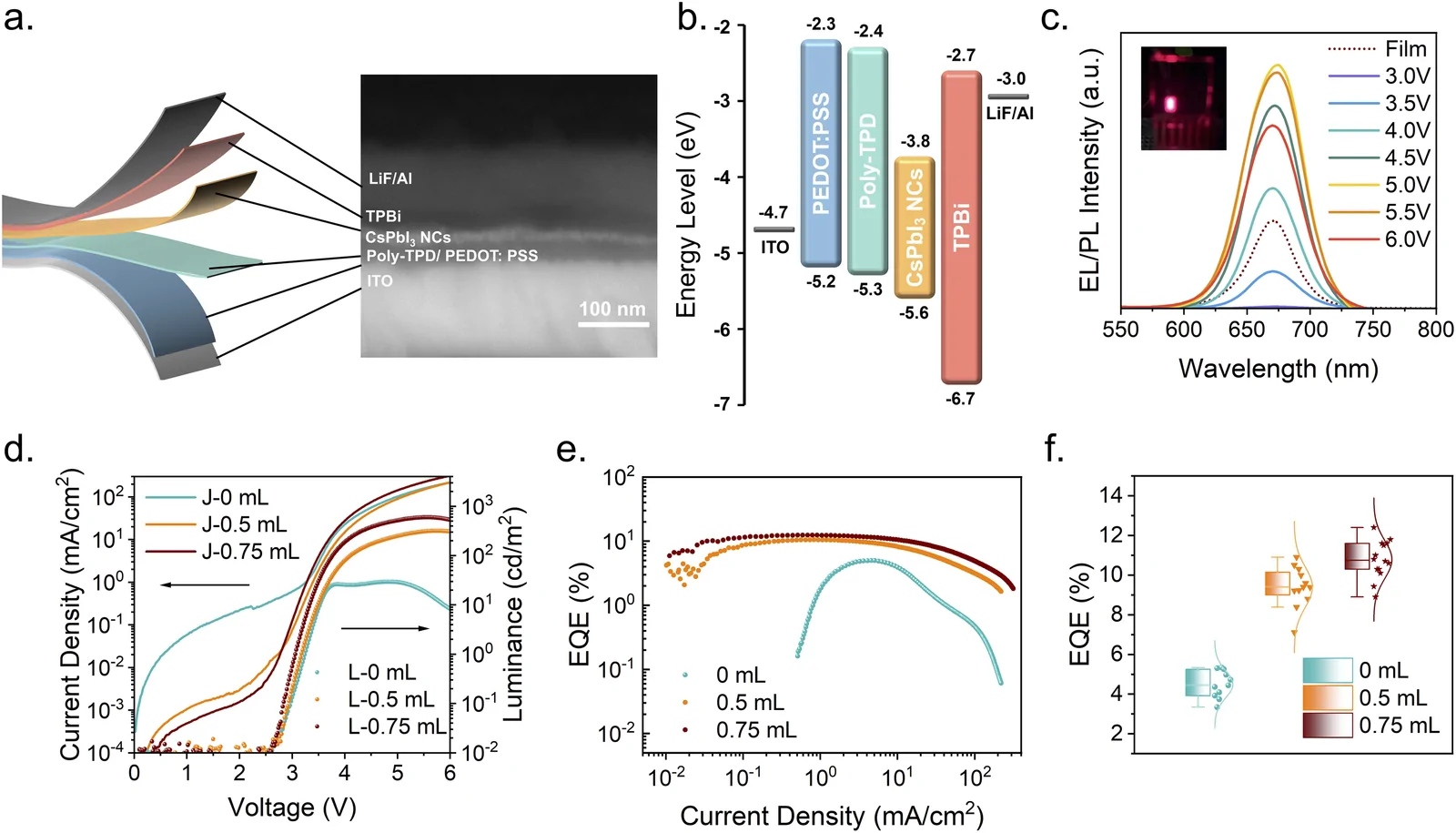
(a) Device architecture of CsPbI_3_ NCs-based LED. Inset: cross-sectional SEM image of a representative LED device. (b) Schematic flat-band energy diagram of the ITO/PEDOT:PSS/Poly-TPD/CsPbI_3_ NCs/TPBi/LiF/Al device architecture. (c) Evolution of the EL spectra of the 0.75 mL TDP-I-treated device under different applied voltages. (d) Current density and luminance as a function of applied voltage (*J*–*V*–*L* characteristics) for LEDs fabricated from pristine and TDP-I-treated CsPbI_3_ NCs. (e) External quantum efficiency (EQE) *versus* current density for the corresponding devices. (f) Statistical distribution of EQEs of 0 mL, 0.5 mL and 0.75 mL TDP-I-treated CsPbI_3_ NCs-based LEDs.

The electroluminescence (EL) spectra of 0.75 mL-TDP-I-treated CsPbI_3_ NC LED at increasing applied bias are presented in Fig. 4c (also see Fig. S29 and S30 for pristine or 0 mL and 0.5 mL TDP-I-treated LEDs in the SI). The EL spectrum closely matched the PL of the corresponding CsPbI_3_ NC films, and no additional EL features were detected.^37,38^ LEDs based on TDP-I-treated NCs exhibited EL at 672 nm for the 0.5 mL TDP-I-treated sample, and at 669 nm for 0.75 mL TDP-I treated NCs, with FWHMs of 50 nm and 49 nm, respectively (Fig. S31 and Table S10). In contrast, the control device reached its maximum luminance at 4.5 V and displayed an EL peak at 679 nm with a narrower FWHM of 41 nm. The EL spectra closely followed the PL characteristics of the corresponding NC films, which showed PL emission maxima at 675 nm (0.5 mL TDP-I), 671 nm (0.75 mL TDP-I), and 680 nm (0 mL); see Table S10 in the SI. The resulting Commission Internationale de l’Éclairage (CIE) coordinates of (0.7065, 0.2934) indicated highly saturated red emission and suggests potential for wide-color-gamut displays when combined with suitable green and blue emitters (Fig. S32).

The beneficial effect of the TDP-I treatment was also evident in the electrical characteristics of the devices. As shown in Fig. 4d, the turn-on voltage, defined as the voltage required to achieve a luminance of 1 cd m^−2^, decreased from 3.26 V (for the control device) to 3.06 and 2.98 V for the 0.5 mL and 0.75 mL TDP-I-treated devices, respectively. This reduction is attributed primarily to the improved surface quality of the TDP-I-treated NC films, which exhibited reduced nonradiative carrier losses, as suggested by PL measurements. The trap-state density was further evaluated using the space-charge-limited current (SCLC) method in hole-only devices. Hole-only devices were fabricated using the following structure: indium tin oxide (ITO)/poly(2,3-dihydrothieno-1,–-dioxin)-poly(styrenesulfonate) (PEDOT:PSS) (30 nm)/poly(4-butylphenyldiphenylamine) (Poly-TPD) (30 nm)/CsPbI_3_ NCs (150 nm)/4,4′-bis(carbazole-9-yl) biphenyl (CBP) (15 nm)/MoO_*x*_ (5 nm)/Au (100 nm) (Fig. S33). Compared with the pristine CsPbI_3_ NC film, the TDP-I-treated samples exhibited lower trap-filled limit voltages (*V*_TFL_), indicating reduced trap densities. The calculated trap-state density decreased from 4.28 × 10^16^ cm^−3^ for the pristine (0 mL) sample to 3.01 × 10^16^ cm^−3^ and 2.86 × 10^16^ cm^−3^ for the 0.5 and 0.75 mL TDP-I-treated samples, respectively. These results confirm that TDP-I effectively suppresses trap states through surface passivation and demonstrate that the TDP-I treatment simultaneously improves charge injection, suppresses leakage currents, and promotes efficient radiative recombination, collectively enabling substantial gains in LED performance. The improved carrier transport in TDP-I-treated CsPbI_3_ NC films could additionally be due to reduction in the interparticle distance in the presence of shorter TDP^+^ ligands compared with the native oleylammonium/oleate ligands, which might facilitate carrier tunnelling between neighbouring NCs. Moreover, the saturated alkyl chains of TDP^+^ can promote more compact ligand packing and better interdigitation than the *cis*-unsaturated oleyl chains. These effects may reduce the barrier for inter-NC charge transport, thereby improving charge injection and transport in the LED devices.

The analysis of the current density–voltage–luminance (*J*–*V*–*L*) characteristics (Fig. 4d) reveals that TDP-I-treated devices exhibited lower current densities before the turn-on regime. The improvements in charge transport and recombination dynamics translate directly into enhanced device performance. While the luminance of the control device began to decrease above 4.8 V, reaching a maximum value of only 30 cd m^−2^ at a current density of 186 mA cm^−2^, the TDP-I-treated devices sustained increasing luminance with increasing bias. Maximum luminance values of 438 cd m^−2^ and 760 cd m^−2^ were achieved for the 0.5 and 0.75 mL TDP-I-treated devices respectively (see the comparison table in the SI, Table S11). The EQE increased from 5.0% for the control device to 10.5% and 12.4% for the 0.5 and 0.75 mL TDP-I-treated devices, respectively (Fig. 4e, and Table S11). To assess the reproducibility of the EQE we calculated the average EQE and luminance from 14 LEDs (Fig. 4f and S34). Device statistics show an average EQE of 10.8% for the optimized 0.75 mL TDP-I-treated LEDs, with a champion EQE of 12.4%. The relatively narrow spread in performance demonstrates good device reproducibility and reveals that the EQE enhancement achieved through TDP-I treatment is consistent.

A comparison of LEDs fabricated with untreated and TDP-I-treated NCs provides insight into the influence of TDP-I treatment on device performance (Fig. 4e). The control device exhibited pronounced efficiency roll-off at current densities exceeding 10 mA cm^−2^, which is tentatively attributed to enhanced nonradiative losses arising from Joule heating and carrier-injection imbalance. Although the current density continued to increase, the emitted luminance decreased, resulting in a sharp reduction in EQE. In contrast, the TDP-I-treated devices maintained substantially higher EQEs over a broader current-density range, highlighting the effectiveness of surface passivation in mitigating efficiency loss under high-injection conditions. Although the maximum EQE achieved in our devices remains below the current state-of-the-art values reported for CsPbI_3_ NC LEDs (∼24%),^39^ it compares favourably with devices employing long-chain-ligand-capped CsPbI_3_ NCs, for which reported EQEs are typically limited to ∼10–21% (see Fig. S35 and Table S12 in the SI).^40–44^

Finally, the operational stability of the LEDs was evaluated under a constant current density of 1 mA cm^−2^ (Fig. S36). The TDP-I-treated devices (0.5 mL, and 0.75 mL) exhibited markedly slower decay than the untreated device (0 mL). The 0.75 mL TDP-I-treated sample exhibited the best operational stability, reaching *T*_50_ after 78 min, while the *T*_50_ of pristine sample was only around 30 min. This improvement is consistent with the reduced defect density and optimized mixed-ligand surface achieved through partial TDP-I ligand exchange.

## Conclusion

In summary, we have demonstrated a phosphonium-based surface engineering strategy for CsPbI_3_ NCs using trimethyl(dodecyl)phosphonium iodide (TDP-I) as a post-synthetic ligand pair. XPS, FTIR, and NMR analyses collectively confirmed the successful incorporation of TDP-I into the native ligand shell through partial ligand exchange. The resulting mixed-ligand surface leads to enhanced optical properties, including an increase in PLQY from ∼77% for pristine NCs to ∼90% for TDP-I-treated samples, together with prolonged photoluminescence lifetimes. In addition, the TDP-I treatment improved the NCs' stability by retarding their degradation and phase transformation under ambient conditions. Molecular dynamics simulations revealed that TDP-I complements the native ligands rather than replacing them entirely, explaining the experimentally observed partial ligand exchange and the onset of NC etching at excessive TDP-I concentrations. The improved surface chemistry translated into better LED performance, with the optimized 0.75 mL TDP-I-treated device achieving a maximum EQE of 12.4%, more than double that of the untreated control device (0 mL). These findings demonstrate that controlled partial phosphonium ligand exchange provides an effective strategy for tailoring the surface chemistry and enhancing the optoelectronic performance of CsPbI_3_ NCs and their corresponding light-emitting devices. Further improvements in device performance will require balancing surface passivation, carrier transport, and colloidal stability. Shorter aromatic phosphonium ligands could further reduce the inter-NC distance while maintaining effective surface passivation, potentially enabling more efficient charge transport. However, short ligands can compromise the colloidal stability of CsPbI_3_ NCs and promote aggregation during solution-phase ligand exchange. Therefore, short-chain phosphonium ligands may be more suitable for future solid-state ligand-exchange strategies.

## Author contributions

S. Z. synthesised and characterised the CsPbI_3_ NCs. M. P. synthesised the ligand. S. Z. and H. R. fabricated and characterised the LED devices. L. G. performed NMR measurements and analysed the resulting data. A. E. A. performed the theoretical calculations and analysed the results. U. F., F. D. B., S. L., Z. L., and Y. S. assisted with CsPbI_3_ NC synthesis and characterisation. I. I. provided guidance on the theoretical calculations and analysis. F. D. S. provided guidance on LED fabrication, characterisation and analysis. M. P. and L. M. conceived the idea and supervised the project. S. Z., M. P., and A. E. A. wrote the original draft. L. G., H. R., I. I., F. D. S., and L. M. contributed to reviewing and editing the manuscript. All authors discussed the results and reviewed the manuscript.

## Conflicts of interest

There are no conflicts to declare.

## Supplementary Material

SC-OLF-D6SC06271G-s001

## Data Availability

The data supporting this study are available within the article and its supplementary Information (SI). Supplementary information: experimental details, TEM characterisation, XRD patterns, XPS measurements, SEM–EDS elemental mapping, NMR analysis, optical property measurements, stability studies, molecular dynamics (MD) simulations, UPS measurements, and LED device performance. See DOI: https://doi.org/10.1039/d6sc06271g.
