# Supplementary material for: Partial ligand exchange on CsPbI3 nanocrystals with alkyl phosphonium iodide improves phase stability and emission efficiency in light-emitting diodes
Source: Chem Sci. 2026 Oct 7;17(41):19736–45. doi: 10.1039/d6sc06271g (PMC13644397; doi:10.1039/d6sc06271g)
Supplement: Supplementary file 1 [file SC-017-D6SC06271G-s001.pdf]

Supplementary Information (SI) for Chemical Science.
This journal is © The Royal Society of Chemistry

Supplementary Information for:

Partial Ligand Exchange on $CsPbI_3$ Nanocrystals with Alkyl Phosphonium Iodide Improves Phase Stability and Emission Efficiency in Light Emitting Diodes

Shence Zhang[a,b], Meenakshi Pegu[a*], Luca Goldoni[c], Hossein Roshan[a], Umberto Filippi[a], Abdessamad El Adel[f], Francesco De Boni[c], Simone Lauciello[d], Zhanzhao Li[a], Yuting Song[a,e], Ivan Infante[f,g], Francesco Di Stasio[h], Liberato Manna[a*]

[a] *Nanochemistry, Istituto Italiano di Tecnologia, Via Morego 30, 16163 Genova, Italy.*
[b] *Department of Chemistry, Materials and Chemical Engineering, Politecnico di Milano, Piazza Leonardo da Vinci 32, 20133 Milano, Italy.*
[c] *Materials Characterization Facility, Istituto Italiano di Tecnologia, Via Morego 30, 16163 Genova, Italy.*
[d] *Electron Microscopy Facility, Istituto Italiano di Tecnologia, Via Morego 30, 16163 Genova, Italy.*
[e] *School of Materials Science and Engineering, Shanghai University, Shangda Road 99, 200444 Shanghai, China.*
[f] *BCMaterials, Basque Center for Materials, Applications, and Nanostructures, Leioa 48940, Spain.*
[g] *Ikerbasque Basque Foundation for Science, Bilbao 48009, Spain.*
[h] *Photonic Nanomaterials, Istituto Italiano di Tecnologia, Via Morego 30, 16163 Genova, Italy.*

Materials.

Cesium carbonate (Cs_2CO_3, reagent plus, 99 %), lead acetate trihydrate ($PbAc_2$. $3H_2O$, 99.99 %), oleic acid (OA, 90 %), Trimethyliodosilane (TMSI, 97 %), octadecene (ODE, technical grade, 90%), toluene (anhydrous, 99.5 %), ethyl acetate (anhydrous, 99.8 %), trimethyl phosphine (PMe_3, 97%), dodecyl iodide (98 %), diethyl ether (HPLC grade, 99.9%) dimethyl sulfone (DMS, TraceCERT Certified Reference Materials for quantitative NMR, 99.99%), toluene d8 (99 atom % D), chloroform-d ($CDCl_3$, 99.8 atom % D), 1,4-dioxane (anhydrous, 99.5 %), chlorobenzene (anhydrous, 99.5 %), dimethylsufoxide-d_6 (DMSO-d6, 99.9 atom % D), lithium fluoride (LiF, 99.9%), and Poly(4-butyltriphenylamine) (Poly-TPD) were purchased from Sigma Aldrich. Poly(3,4ethylenedioxythiophene): poly(styrene sulfonate) (PEDOT: PSS) with ratio of 1:6 (AI 4083) was purchased from Ossila. Patterned indium tin oxide (ITO) substrates and 2',2'-(1,3,5-benzinetriyl)-tris(1-phenyl-1-H-benzimidazole) (TPBi, 98%) were purchased from Lumtech. Aluminum pallets were purchased from Ted Pella for evaporation use. Unless otherwise stated, all the materials are used without further purification.

General procedure for the synthesis.

Synthesis of trimethyl(dodecyl)phosphonium iodide (TDP-I) ligand.

1-Iodododecane (5.5 mmol) was dissolved in anhydrous toluene (10 mL) in an oven-dried three-neck Schlenk flask equipped with a magnetic stir bar and degassed for 5 min at room temperature. Trimethylphosphine (5 mmol, 5 mL) was added dropwise under a nitrogen atmosphere, and the reaction mixture was heated to 80 °C

and refluxed for 18 h. After completion of the reaction, the solvent was removed under reduced pressure to afford a crude white solid. The product was purified by washing with diethyl ether (5 x 20 mL) using a Büchner funnel and dried under vacuum at room temperature to yield trimethyl(dodecyl)phosphonium iodide (TDP-I) as a white solid (1.4 g). The chemical structure and purity of TDP-I were confirmed by ^{1}H and ^{13}C NMR spectroscopy in toluene-d8 (see Figures S1-S2)

Scheme S1. Synthesis route for trimethyl(dodecyl)phosphonium iodide (TDP-I).

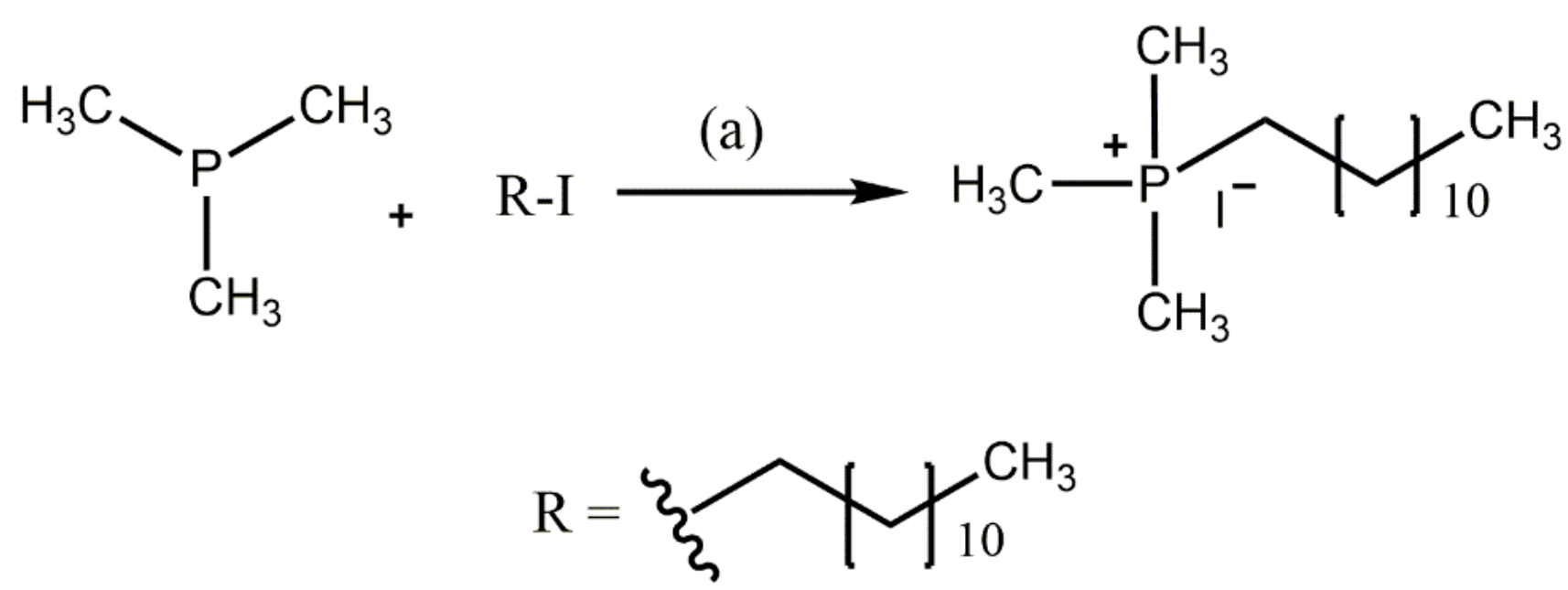

Reaction condition: (a) 10 mL toluene, 80°C, 18h reflux.

Preparation of Cs-Pb-Oleate stock solution.

2 mmol Pb $(CH_3COO)_2{\cdot}3H_2O$, 0.5 mmol Cs_2CO_3, and 5 mL oleic acid were loaded into a 40 mL vial and degassed under vacuum for 1.5 hours at 120 °C. Then the solution was cooled down to room temperature and stored in a glove box.

Synthesis of $CsPbI_3$ NCs.

The pristine $CsPbI_3$ NCs were synthesized by previously reported TMS-I hot injection method with slight modification.[1] 0.5 mL Cs-Pb-Oleate stock solution, 0.25 mL Oleylamine (OLAM), and 9 mL of 1-octadecene

(ODE) were loaded into a 25 mL 3-neck round-bottom flask and degassed under vacuum for 45 mins at 120 °C. Subsequently, the temperature increased to 150 °C under N_2, and 0.4 mL TMS-I (100 μL) in degassed ODE (300 μL) was injected swiftly. After 10 seconds, the reaction mixture was cooled down in an ice-water bath and directly used for ligand exchange.

Ligand exchange of $CsPbI_3$ NCs.

The crude $CsPbI_3$ NC dispersion was divided into four equal fractions (2 mL each). One fraction was retained as the pristine reference (0 mL TDP-I), while the remaining three fractions were subjected to ligand exchange with TDP-I. Since TDP-I exhibits limited solubility in nonpolar solvents such as toluene, a 25 mM stock solution was prepared by dissolving TDP-I in a mixture of chloroform (0.3 mL) and toluene (2.7 mL) at 80 °C for 5 min. Aliquots of 0.5 mL, 0.75 mL, or 1.0 mL of the TDP-I stock solution were then added to separate 2 mL portions of the crude $CsPbI_3$ NC dispersion, and the mixtures were stirred vigorously for 20 min at room temperature.

Following ligand exchange, the NCs were collected by centrifugation at 6000 rpm for 5 min. The supernatant was discarded, and the precipitate was redispersed in anhydrous toluene (1 mL). Anhydrous ethyl acetate (2 mL) was subsequently added as an antisolvent, followed by centrifugation at 6000 rpm for 3 min. The precipitate was collected and redispersed in anhydrous toluene (1 mL). This purification step was repeated once using only anhydrous toluene. The pristine $CsPbI_3$ NCs were purified following the same protocol in the absence of TDP-I. The final purified NC dispersions were redispersed in 0.5 mL of anhydrous toluene. All

purification steps were carried out using anhydrous solvents to minimize moisture-induced degradation of the $CsPbI_3$ NCs.

Device Fabrication

Patterned indium tin oxide (ITO) glass substrates were sequentially cleaned by ultrasonication in ITO cleaning solution, deionized water, acetone, and isopropanol for 20 min each, followed by drying under a nitrogen flow. The substrates were subsequently treated with oxygen plasma for 3 min. A PEDOT: PSS layer was spin-coated onto the cleaned ITO substrates at 4000 rpm for 40 s and annealed at 130 °C for 20 min in air. The substrates were then transferred to a nitrogen-filled glovebox, where a poly-TPD solution (6 mg mL^{-1} in chlorobenzene) was spin-coated at 2000 rpm for 45 s, followed by annealing at 130 °C for 20 min. The emissive layer was deposited by spin-coating a $CsPbI_3$ NC dispersion (20 mg mL^{-1} in toluene) at 3000 rpm for 45 s. Subsequently, TPBi (30 nm), LiF (1 nm), and Al (100 nm) were sequentially deposited by thermal evaporation through a shadow mask under a base pressure of 3×10^{-6} Torr. The thickness of each layer was monitored using a quartz crystal microbalance. The active device area, defined by the overlap of the ITO and Al electrodes, was approximately 4 mm^2.

LED Characterization.

Current density-voltage-luminance (*J-V-L*) characteristics were measured using a Keithley 2636 Source Meter coupled to a calibrated PDA100A Si photodetector (Thorlabs). The detector output was converted to optical power (photon flux) using the calibrated spectral responsivity of the photodetector. Luminance was determined by collecting the light emitted through the ITO/glass substrate without the use of an integrating sphere. The

external quantum efficiency (EQE) was calculated from the ratio of the emitted photon flux to the injected charge. Electroluminescence (EL) spectra were recorded using an Edinburgh Instruments FLS1000 fluorescence spectrometer in step-scan mode. All measurements were performed under ambient conditions (∼50% relative humidity).

Methods.

Powder X-ray diffraction (XRD) analysis.

X-ray diffraction (XRD) patterns were collected using a PANalytical Empyrean diffractometer equipped with a 1.8 kW Cu Kα ceramic X-ray source and a PIXcel3D 2 × 2 area detector, operating at 45 kV and 40 mA in parallel-beam geometry under symmetric reflection mode. The NC samples were prepared by concentrating the colloidal dispersions under a nitrogen atmosphere, followed by drop-casting onto zero-background Si substrates. All measurements were performed at room temperature under ambient conditions.

Transmission Electron Microscopy (TEM).

Transmission electron microscopy (TEM) images were acquired using a JEOL JEM-1400Plus microscope operated at an accelerating voltage of 120 kV. TEM samples were prepared by drop-casting dilute NC dispersions onto 200-mesh carbon-coated copper grids.

Scanning Electron Microscopy (SEM).

Energy-dispersive X-ray spectroscopy (EDS) was performed on NC films spin-coated onto Si substrates using a ZEISS GeminiSEM 560 field-emission scanning electron microscope equipped with a Schottky field emitter and an Oxford X-Max 80 silicon drift detector (SDD, 80 mm^2 active area), operated at an accelerating voltage of 10 kV.

Optical Absorption and Photoluminescence Spectroscopy.

Optical Characterization. UV-visible absorption spectra of the NC dispersions and thin films were recorded using Varian Cary 300 and Varian Cary 5000 spectrophotometers, respectively. Photoluminescence (PL) spectra of the NC dispersions and thin films were measured using a Varian Cary Eclipse fluorescence spectrophotometer and an Edinburgh Instruments FLS900 spectrometer, respectively, with an excitation wavelength of 500 nm for all measurements. Solution-phase measurements were performed using dilute NC dispersions in toluene loaded into 1 cm path-length quartz cuvettes.

Photoluminescence Quantum Yield (PLQY) and Time-Correlated Single-Photon Counting (TCSPC).

Photoluminescence quantum yields (PLQYs) were measured using an Edinburgh Instruments FLS900 spectrophotometer equipped with an integrating sphere. Spectra were collected with a step size of 0.5 nm and an integration time of 0.2 s per data point. The NC samples were prepared by diluting 10-15 μL of concentrated NC dispersion in 2.5 mL of anhydrous toluene in 1 cm path-length quartz cuvettes, sealed with PTFE stoppers (Hellma Analytics, part no. 111-10-40). A blank reference was prepared using 2.5 mL of anhydrous toluene. Prior to PLQY measurements, all samples were diluted to an optical density of ∼ 0.10 - 0.12 at 500 nm and measured using the integrating sphere under excitation from the continuous xenon lamp.

Time-resolved photoluminescence (TRPL) measurements were performed at room temperature using the same Edinburgh Instruments FLS900 spectrophotometer equipped with a pulsed laser diode (λex = 375 nm, repetition rate 10 MHz, pulse width 60 ps). The PL decay curves were fitted using a biexponential decay model.

X-ray Photoluminescence Spectroscopy (XPS).

X-ray photoelectron spectroscopy (XPS) measurements were carried out through a Kratos Axis UltraDLD spectrometer (Kratos Analytical Ltd.) with a monochromated Al Kα X-ray source (hν = 1486.6 eV) operating at 20 mA and 15 kV. Specimens were prepared by dropping a concentrated NCs solution in toluene onto a highly ordered pyrolytic graphite (HOPG, ZYA grade) substrate. The wide scans were collected over an analysis area of 300 × 700 μm^2 at a photoelectron pass energy of 160 eV and energy step of 1 eV, while high-resolution spectra of Cs 3d, Pb 4f, Br 3d, and C 1s, are collected at a photoelectron pass energy of 20 eV and an energy step of 0.1 eV. A take-off angle of 0° concerning the sample's normal direction was used for all analyses. The slight differential electrical charging effects (less than 0.5 eV) observed on all samples were not neutralized. The irradiation of an $APbX_3$-like perovskite with an electron beam is known to induce the desorption of halogen species and the nucleation of metallic Pb particles. The spectra have been referenced to the adventitious carbon 1s peak at 284.8 eV. The spectra were analyzed with the Casa XPS software (Casa Software Ltd., version 2.3.24), and the residual background was eliminated by the Shirley method across the binding energy range of the peaks of interest. The relative atomic concentrations were then estimated using the specific function in the Casa XPS software.

Ultraviolet photoelectron spectroscopy (UPS)

The ultraviolet photoelectron spectroscopy (UPS) measurements were performed using a He I (21.22 eV) discharge lamp, fitted in the same chamber used for XPS analyses, on an area of 55 μm in diameter, at a pass energy of 10 eV and with a dwell time of 100 ms. The energy levels within the equipment and UPS-based calculations are described as follows. The work function, φ, i.e. the position of the Fermi level versus the vacuum level, was determined for each sample from the position of the secondary electron cutoff in the UPS spectrum, using the following equation: φ = hv - E0, where hν is the source energy (21.22 eV for He I photons) and E0 is the secondary electron cut off. The position of the valence band maximum (VBM) versus the vacuum level, i.e. the ionization energy, Eion, was determined from the width of the entire UPS spectrum, according to the following equation: $E_{ion} = hv - (E_0 - E_1) = hv - E_0 + E_1$, where E_1 is the position of the VBM concerning the zero (Fermi) level. The values E0 and E1 were determined in the UPS spectrum through the background functions "Edge Up" and "Edge Down", respectively, in the Casa XPS software. The error bar associated with this procedure was estimated to be equal to 0.2 eV.

Nuclear magnetic resonance (NMR).

The NMR experiments were acquired at 298K on a Bruker 600 MHz spectrometer fit with a 5 mm QCI cryoprobe. In the $^{1}H\,NMR$ spectrum 128 transients were accumulated by using 65536 digit points and 30 s of inter pulses delay, over a spectral width of 20.83 ppm, with the offset positioned at 6.18 ppm and the receiver fixed at 18. An exponential line broadening equivalent to 1 Hz for the NCs dispersed in toluene-D_8 and 0.3 Hz for the NCs dissolved in DMSO-d_6 respectively were applied to FIDs (Free Induction Decays) prior to the Fourier transforms, followed by a polynomial baseline correction.

The *^{1}H–^{1}H NOESY* (Nuclear Overhauser Effect Spectroscopy) experiment (noesygpphpp, Bruker's library) was acquired with 80 scans, after 32 steady ones, by using 2048 digit points, 256 increments, and a mixing time of 300 ms, over a spectral width of 15.15 ppm (offset positioned at 7.49 ppm).

In the *^{1}H-^{13}C HSQC* (Heteronuclear Single-Quantum Correlation sensitive improved) spectrum was collected with 256 scans, 2048 digit points and 256 increments, an inter pulses delay of 1.5 s and a J_{CH} filter of 145 Hz, over a spectral width of 13.02 ppm for ^{1}H and 165.0 ppm for ^{13}C, with the transmitter frequency offsets positioned at 4.7 and 75.0 ppm, respectively.

All the chemical shifts were referred to the not deuterated toluene solvent peak at 7.09 ppm for ^{1}H and 129.24 ppm for ^{13}C, and not deuterated DMSO solvent peak at 2.50 ppm for ^{1}H and 39.52 ppm for ^{13}C respectively.

Ligand quantification.

The concentration of different ligands (trimethyl(dodecyl)phosphonium, oleylammonium, and oleate) was quantified using quantitative NMR spectroscopy by comparing the integrated area of ligand signals, each normalized for the number of resonances generating the peak (2H), to that of a solution of dimethyl sulfone 10 mM (TraceCERT®) used as a standard solution, in DMSO-d_6, the latter normalized to 6 protons by using the PULCON[1] (Pulse Length-based Concentration determination) method.

Inductively Coupled Plasma–Optical Emission Spectroscopy (ICP-OES).

After the ligand quantification on the deuterated DMSO solution, the Pb concentration of the NCs was estimated by ICP-OES on an aiCAP 6000 spectrometer (Thermo Scientific) Prior to the measurement, 50 μL

of the DMSO-d_6 sample were diluted to 25 mL in an aqueous solution of aqua regia (1:25) and subjected to an acid digestion overnight.

Ligand-shell composition analysis.

The Ligand-shell composition of the samples was estimated by NMR and ICP-OES, respectively. The concentrations of the ligand and Pb were obtained after evaporating the colloidal solution of the NCs in deuterated toluene (previously characterized). The resulting precipitate was thoroughly dried under a nitrogen flow and subsequently dissolved in deuterated-DMSO (200 μL) containing 5 μL of Trifluoro acetic acid (TFA) and transferred into a 3 mm Samplejet tube for NMR ligand quantification analysis.

The total number of ligands is based on the ligand's concentration in the DMSO-d6 sample and total volume of sample (measured by NMR):

$$N^{o}\ \text{Ligands} = \text{Concentration of ligand}\ [M] * 200\ \text{ul}$$

The total number of NCs is determined by the Pb concentration in the DMSO-d6 sample (measured by ICP analysis) and the size distribution of the NCs considering a unit cell size (a=0.8856 nm, b=1.2472 nm, c=0.8577 nm):

$$N^{o}\ \text{Pb atoms} = \text{Concentration of Pb atoms}\ [M] * 200\ \text{ul}$$

$$N^{o}\ \text{Pb atoms/NC} = \text{NCs volume}/(a * b * c) * 4$$

$$N^{o}\ \text{NCs} = \frac{N^{o}\ \text{Pb atoms}}{N^{o}\ \text{Pb atoms/NC}}$$

The number of each type of ligand on the surface of each NCs:

$$N^o \text{ Ligands/NC} = N^o \text{ Ligands}/N^o \text{ NCs}$$

Molecular Dynamics Simulation.

All MD simulations were performed using a custom force field optimized for the $CsPbI_3$/ligand system using the Auto-FOX package[2-3]. The toluene solvent was modeled using a coarse-grained (CG) representation[4]. Non-bonded interactions were described by Lennard-Jones (LJ) and Coulomb terms. A summary of the key force field parameters is provided in Table S8. Five simulation systems were constructed to probe the effect of ligand identity and surface density on the structural stability of $CsPbI_3$ NCs: (i) high-density OLAm-capped, (ii) high-density TDP-I-capped, (iii) low-density OLAm-capped, (iv) low-density TDP-I-capped, and (v) a mixed OLAm/TDP-I-capped model approximating the partial-exchange surface composition observed experimentally. In all cases, the $CsPbI_3$ NC core was constructed as a cubic nanocrystal with an edge length of 5.6 nm, comprising a charge-balanced composition of Cs₉₇₂Pb₇₂₉I₂₄₃₀. Ligands were placed on the NC surface using CAT package[5] at the following surface densities: 441 molecules (2.3 molecules/nm^2) for the high-density models, 191 molecules (1 molecules/nm^2) for the low-density models, and 299 OLAm + 161 TDP-I molecules, corresponding to a mixed ligand shell composed of 65% OLAm and 35% TDP-I by molecule count for the mixed model. Each QD system was solvated in a 20 nm cubic simulation box containing 44,966 coarse-grained toluene molecules, using PACKMOL[6] with a minimum distance of 1 nm between the QD surface and the box boundary to avoid periodic image interactions. All simulations were performed using GROMACS 2024.2[7-16]. Each system was first subjected to energy minimization using the steepest descent algorithm, with

a maximum force convergence threshold of 100.0 kJ mol⁻^{1} nm⁻^{1} and a maximum of 10,000 steps. Energy minimization was followed by a 1 ns NVT equilibration at 300 K using the V-rescale thermostat[17] with a time constant of 1.0 ps, with initial velocities assigned from a Maxwell–Boltzmann distribution at 300 K. Subsequently, a 2 ns NPT equilibration was performed at 300 K and 1 bar using the V-rescale thermostat (τ = 0.1 ps) and the Berendsen barostat[18] with isotropic pressure coupling. Production runs of 50 ns were then performed in the NPT ensemble at 300 K and 1 bar, using the V-rescale thermostat (τ = 0.1 ps) and the Parrinello–Rahman barostat[19] with isotropic pressure coupling. A timestep of 1 fs was used throughout all simulation stages. Bonds involving hydrogen atoms were constrained using the LINCS algorithm[20]. Long-range electrostatic interactions were treated using the Smooth Particle Mesh Ewald (SPME) method[21] with a fourth-order interpolation, a Fourier spacing of 0.15 nm, and a real-space cutoff of 1.0 nm. Van der Waals interactions were truncated at 1.0 nm using a potential-shift modifier. Periodic boundary conditions were applied in all three dimensions. Structural analyses were performed on the production trajectories using GROMACS built-in analysis tools. Prior to all analyses, trajectories were corrected for periodic boundary conditions, and the QD center of mass drift was removed. The root-mean-square deviation (RMSD) of the inorganic core atoms was calculated, with the initial frame of the production trajectory as the reference configuration. Ion-resolved RMSD profiles for Cs⁺ and I⁻ were obtained by applying the same analysis independently to each ionic species using the same reference frame. The root-mean-square fluctuation (RMSF) of the core atoms and the radius of gyration (Rg) were computed averaged over the full production trajectory. The per-ligand ligand–core interaction energy was calculated via energy-group decomposition as implemented in GROMACS. The QD inorganic core and each ligand type were defined as separate energy groups in the .mdp input file, and

interaction energies between the ligand group and the core group — comprising short-range Coulomb (Coul-SR) and Lennard-Jones (LJ-SR) contributions — were extracted using gmx energy from a rerun of the production trajectory with the updated energy-group definitions. The total per-ligand ligand–core interaction energy was computed as $E_{int} = (E_{Coul\text{-}SR} + E_{LJ\text{-}SR}) / N_{lig}$, where N_{lig} is the number of ligand molecules of the corresponding type in each system. Coulomb and Lennard-Jones contributions were also extracted separately to assess the relative electrostatic and dispersive character of each ligand–surface interaction (Figure S25).

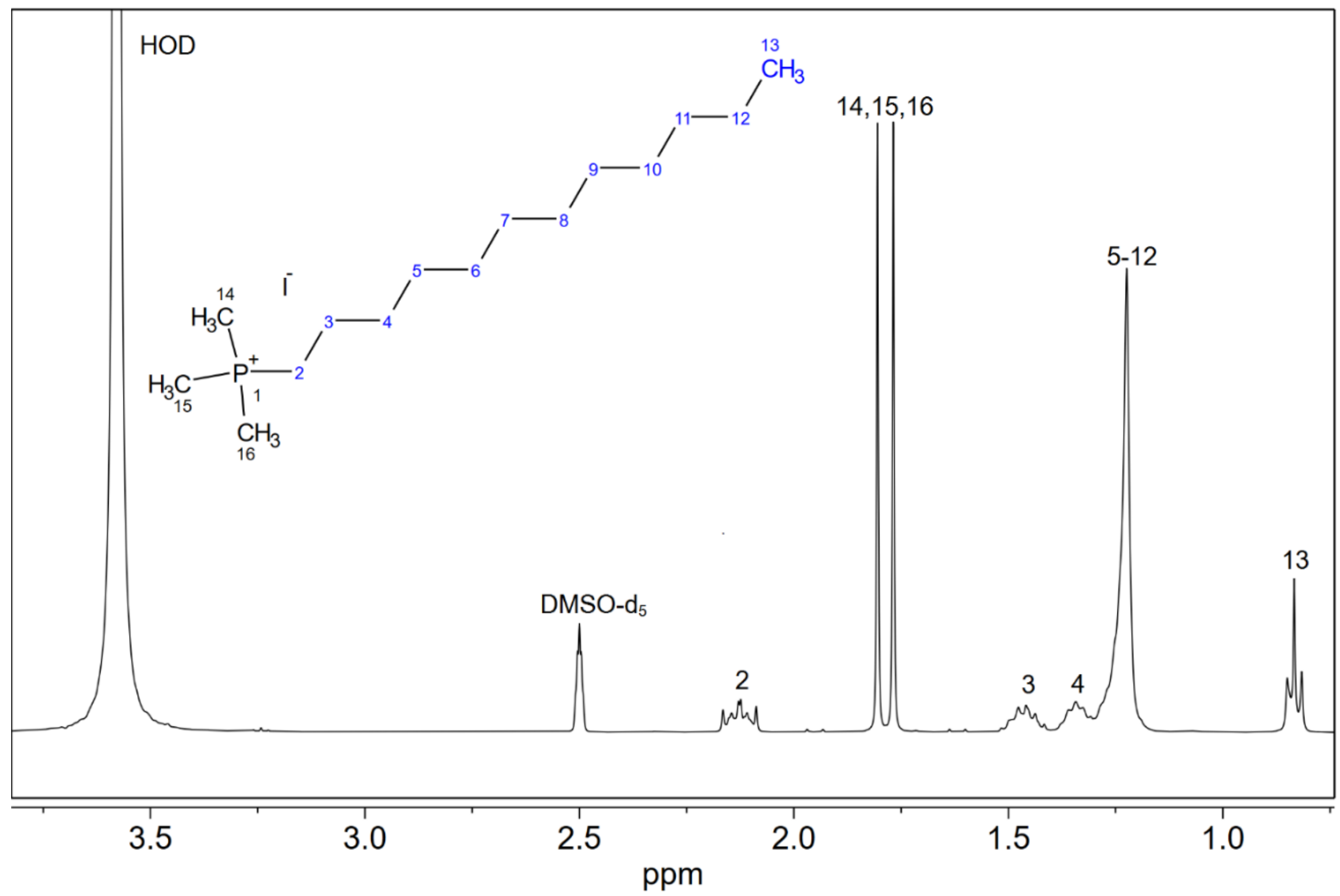

Figure S1: ^{1}H NMR spectrum of TDP-I in DMSO-d6, with the peak assignment, the TDP-I structure formula with 1H numbering. The CH_3 protons (i.e. 14,15,16) are magnetically equivalent but split into a doublet for $J_{^1H\text{-}^{31}P}$ coupling = 14.76 Hz.

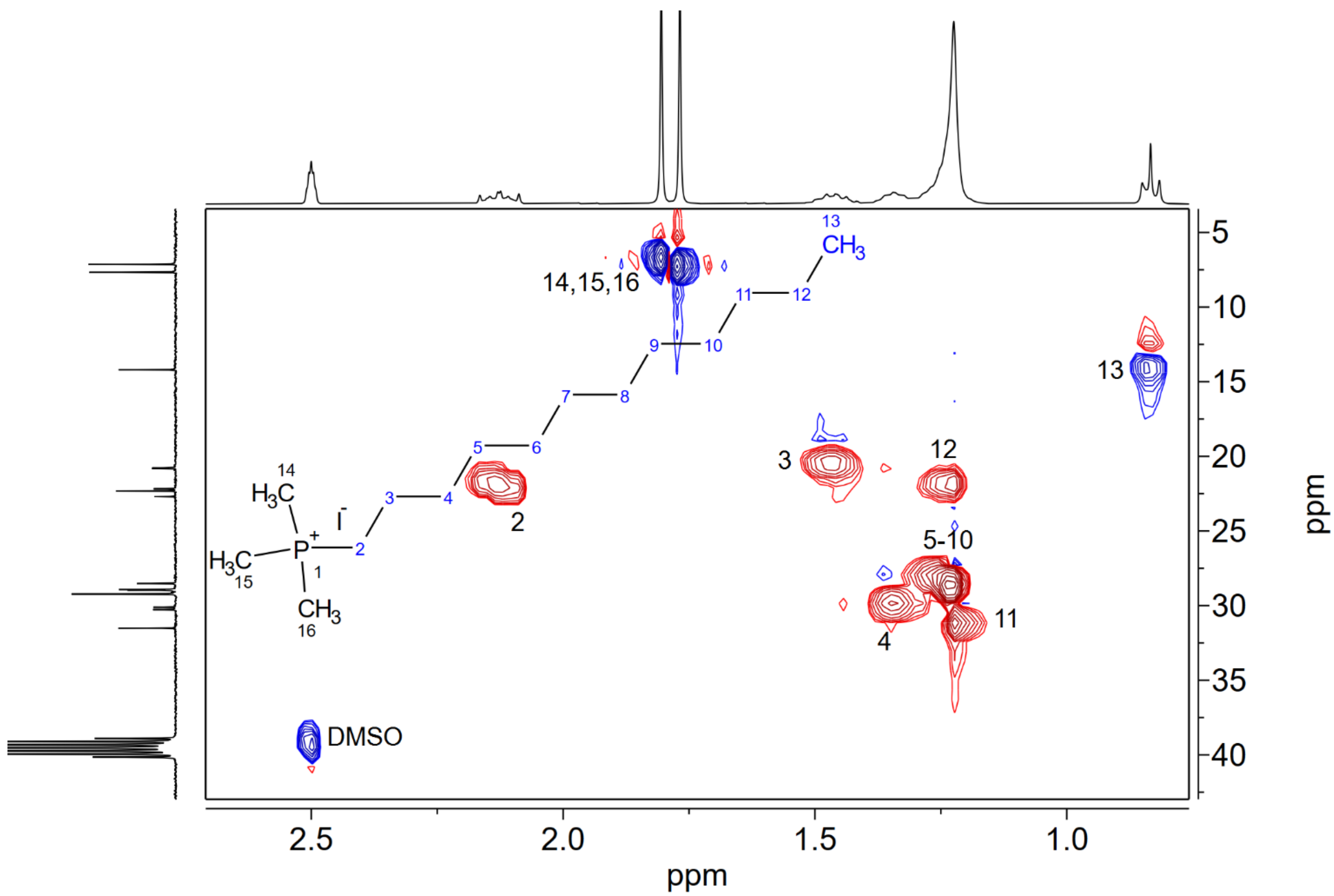

Figure S2: ^{1}H-^{13}C HSQC NMR experiment of TDP-I in DMSO-d6, with the peak assignment, the TDP-I structure formula with numbering. In the edited version the CH and CH_3 cross peaks are represented in blue whereas the CH_2 are shown in red.

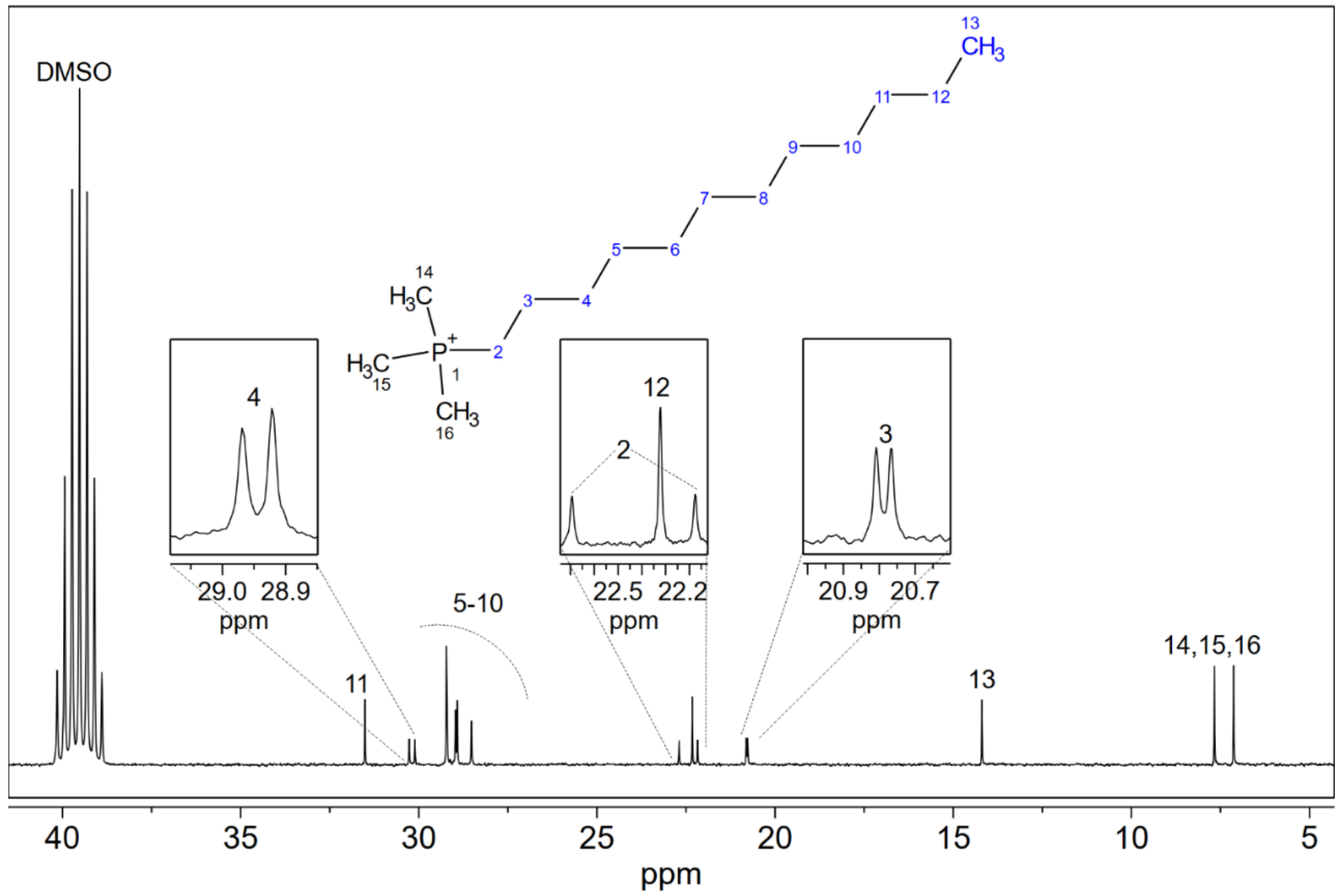

Figure S3: ^{13}C {^{1}H} decoupled NMR spectrum of TDP-I in DMSO-d6 with the peak assignment and the TDP-I structure formula with the carbon numbering. The insert reported the ^{13}C signal splitting due to the J coupling between ^{13}C and ^{31}P.

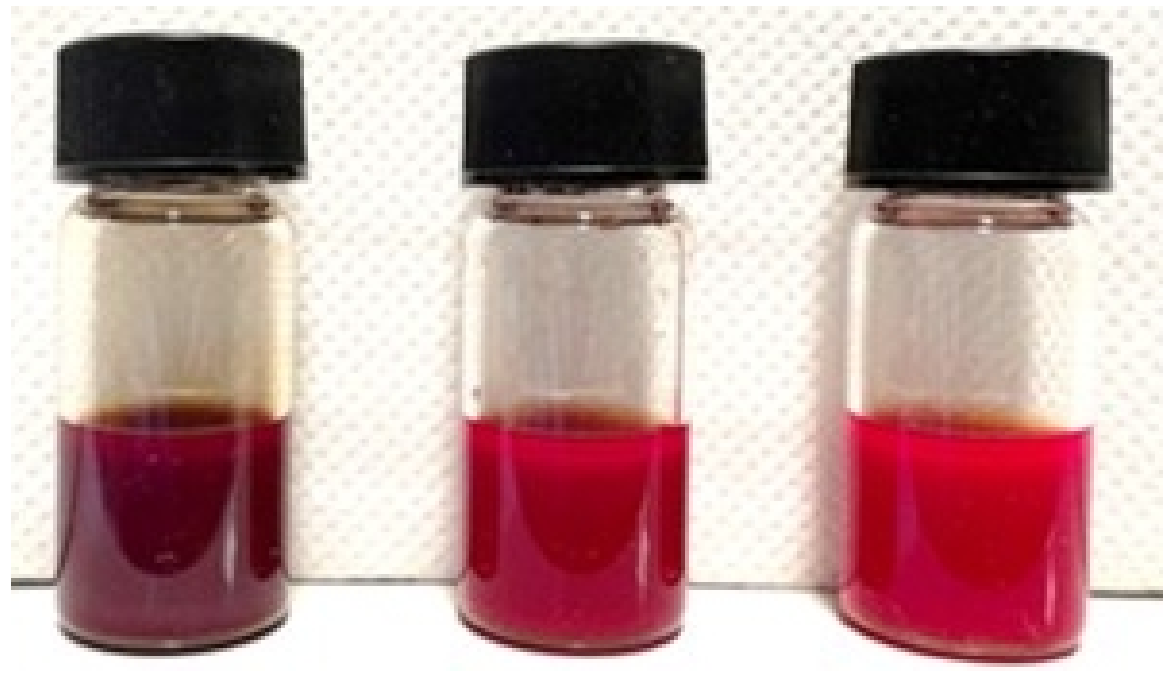

Figure S4: Color of 0 mL, 0.5 mL and 0.75 mL TDP-I-treated $CsPbI_3$ NCs solution.

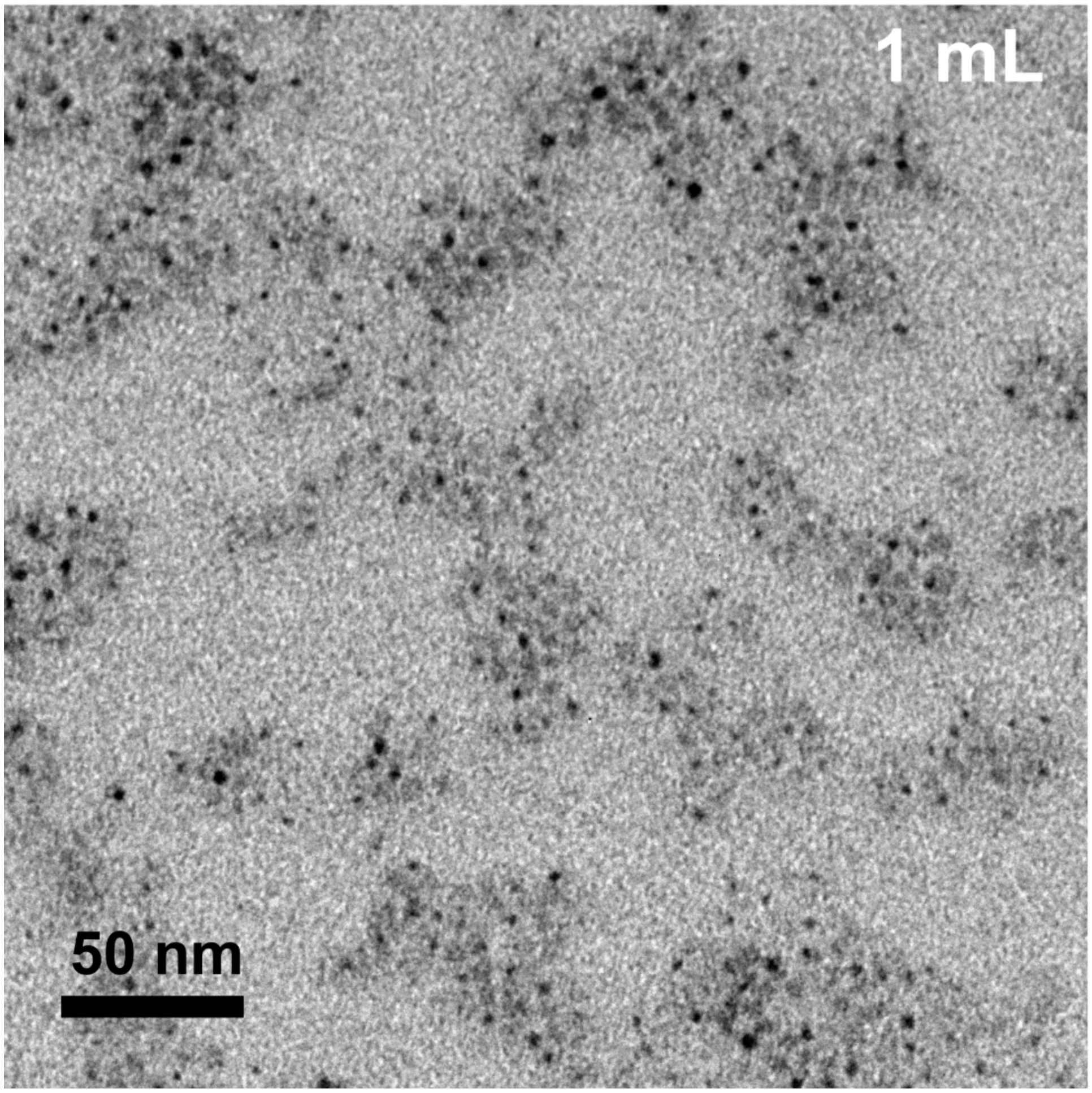

Figure S5: TEM micrographs of 1 mL TDP-I-treated $CsPbI_3$ NCs.

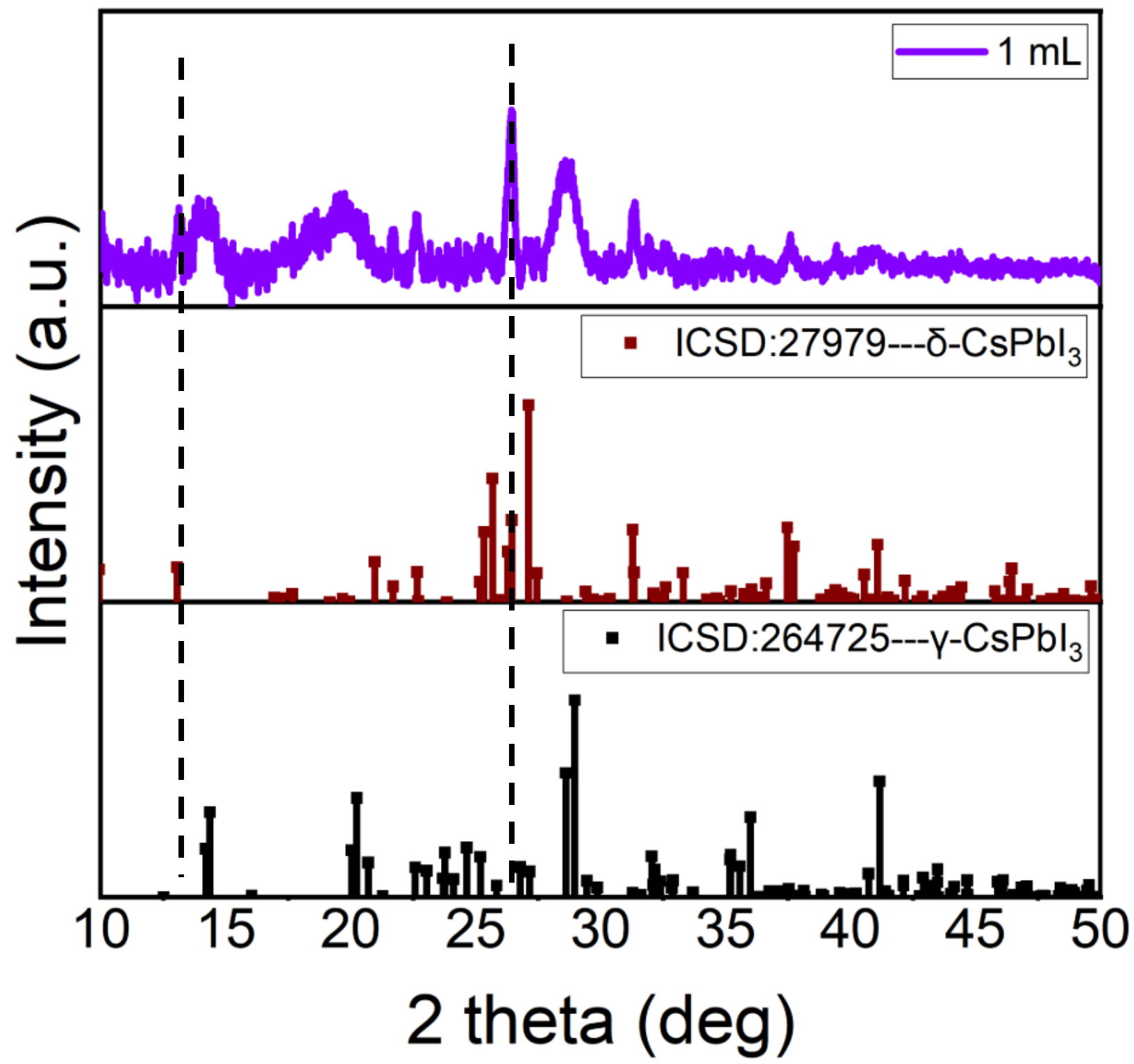

Figure S6: X-ray diffraction (XRD) patterns of 1 mL TDP-I-treated $CsPbI_3$ NCs together with the reference pattern of orthorhombic γ-$CsPbI_3$ (ICSD No. 264725) and orthorhombic δ-$CsPbI_3$ (ICSD No. 27979).

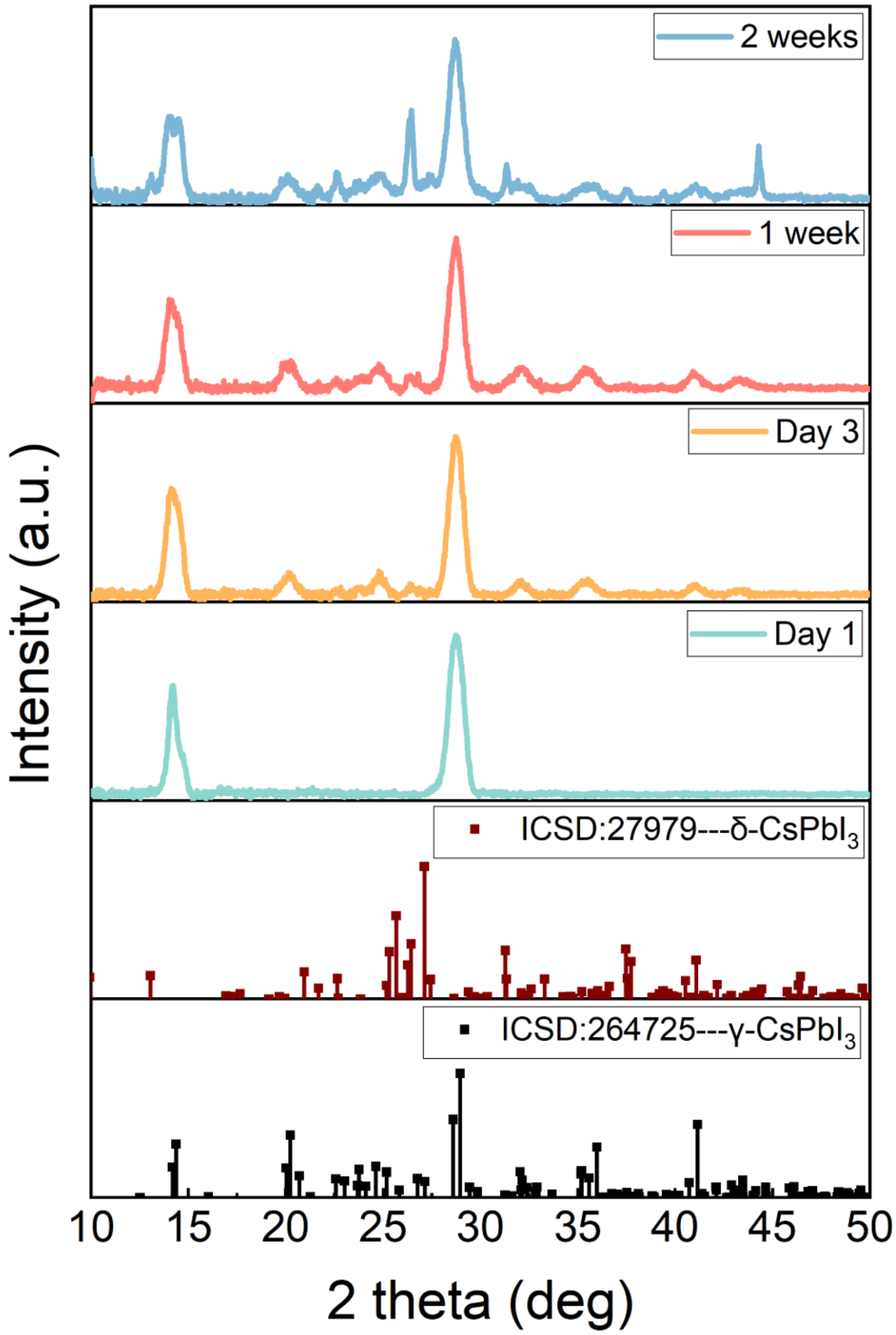

Figure S7: X-ray diffraction (XRD) patterns of prinstine $CsPbI_3$ NCs (control sample) after two weeks of storage in the air.

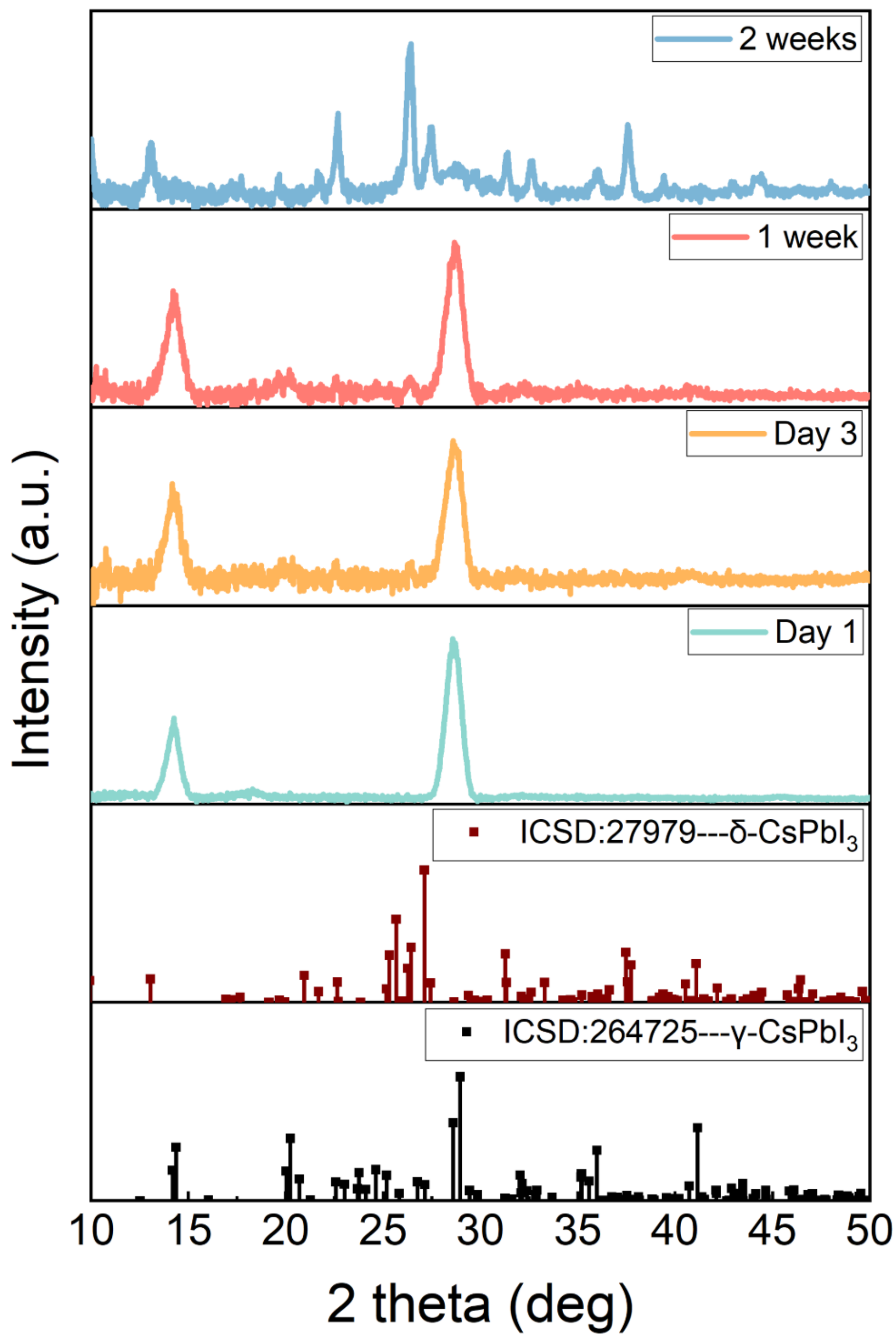

Figure S8: X-ray diffraction (XRD) patterns of 0.5 mL TDP-I-treated $CsPbI_3$ NCs after two weeks of storage in the air.

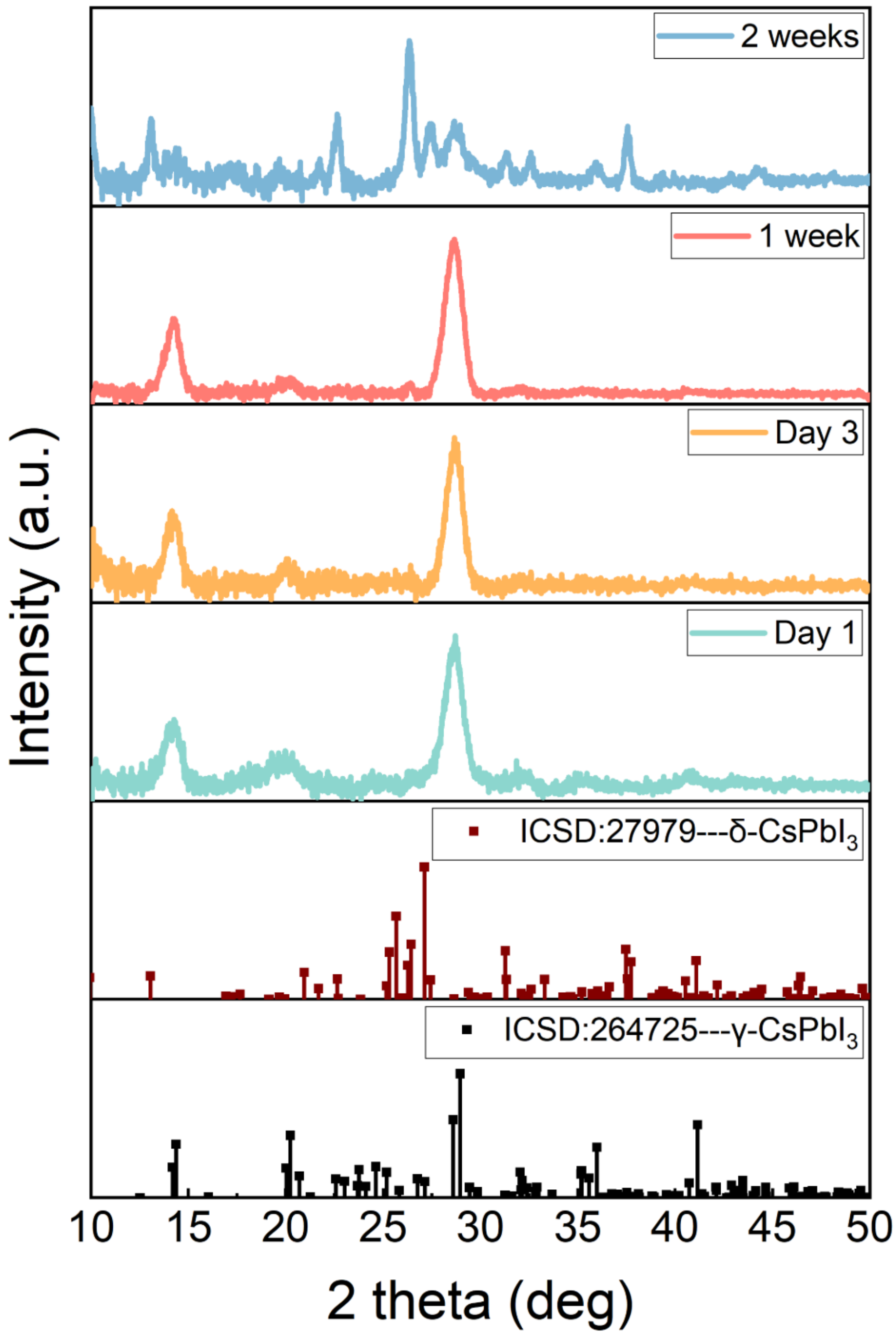

Figure S9: X-ray diffraction (XRD) patterns of 0.75 mL TDP-I-treated $CsPbI_3$ NCs after two weeks of storage in the air.

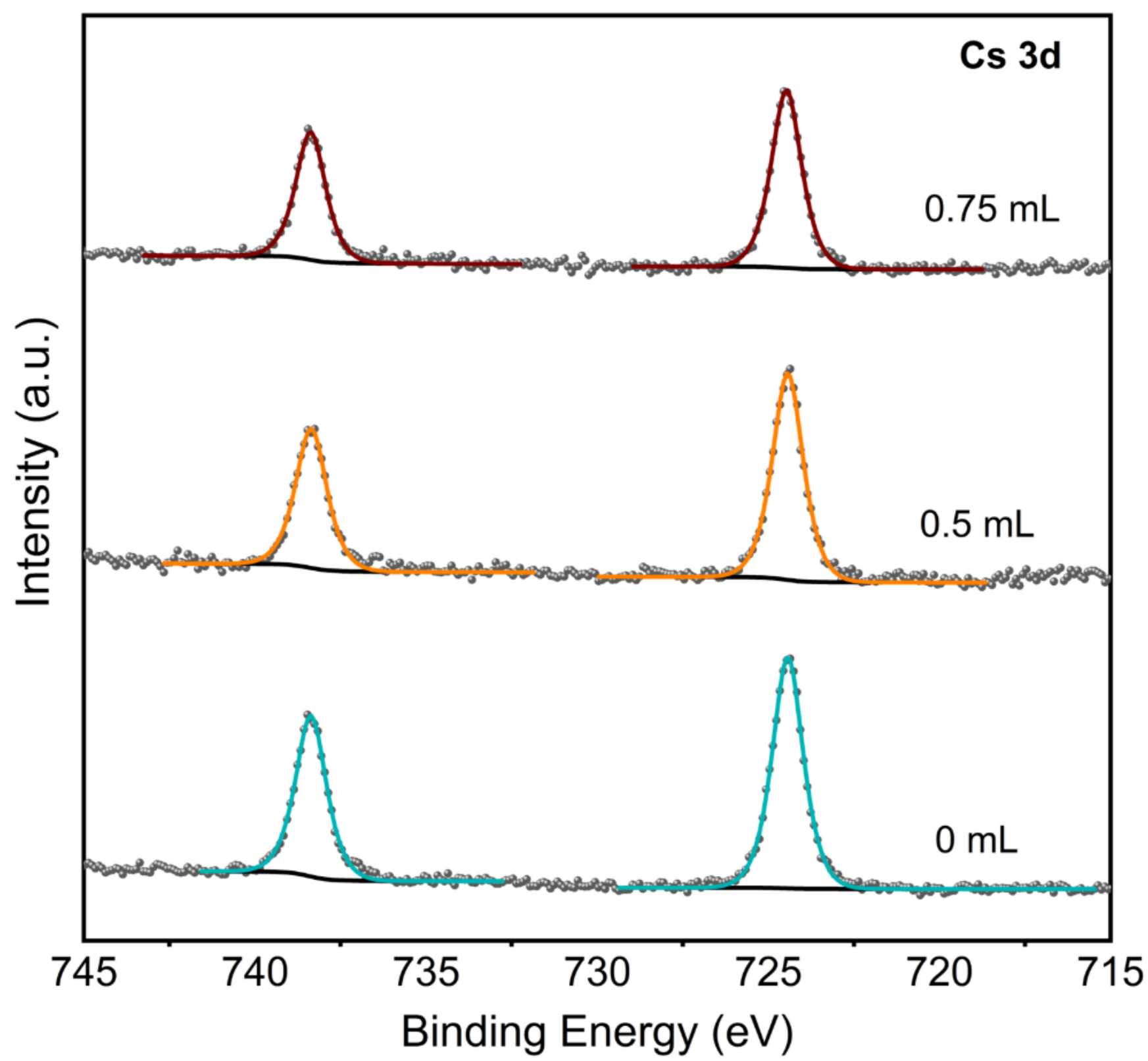

Figure S10: Comparison between Cs 3d XPS spectra of 0 mL, 0.5 mL, and 0.75 mL TDP-I-treated $CsPbI_3$ NCs. The Cs 3d spectra remain essentially unchanged after TDP-I treatment, with no significant binding-energy shifts or new Cs species observed. This indicates that the Cs⁺ framework is well maintained during the surface ligand-exchange process.

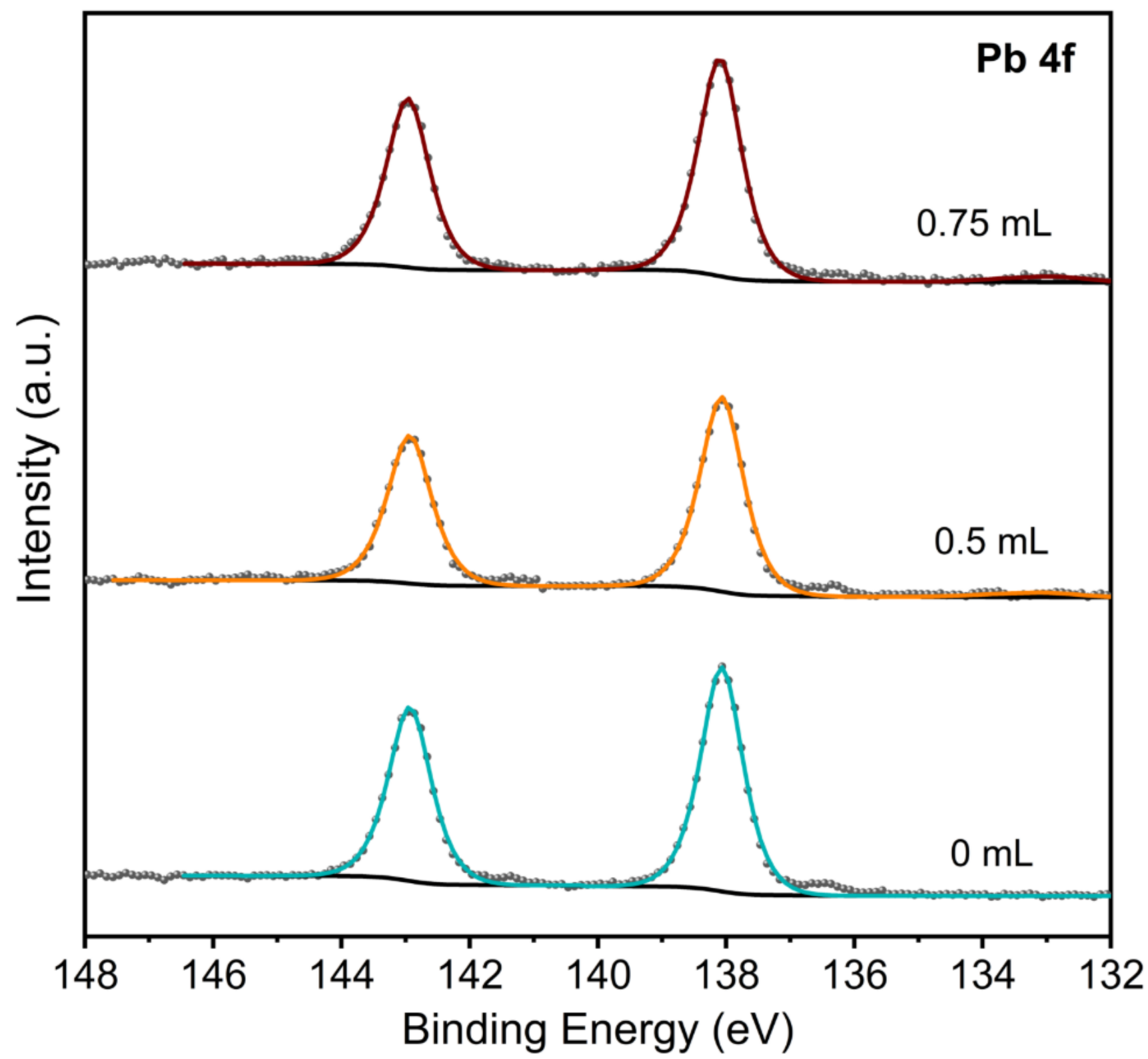

Figure S11: Comparison between Pb 4f XPS spectra of 0 mL, 0.5 mL and 0.75 mL TDP-I-treated $CsPbI_3$ NCs. The Pb 4f spectra remain essentially unchanged after TDP-I treatment, with no noticeable binding-energy shifts or additional Pb species. These results indicate that the Pb^{2}⁺ framework of $CsPbI_3$ is well preserved during the ligand-exchange process.

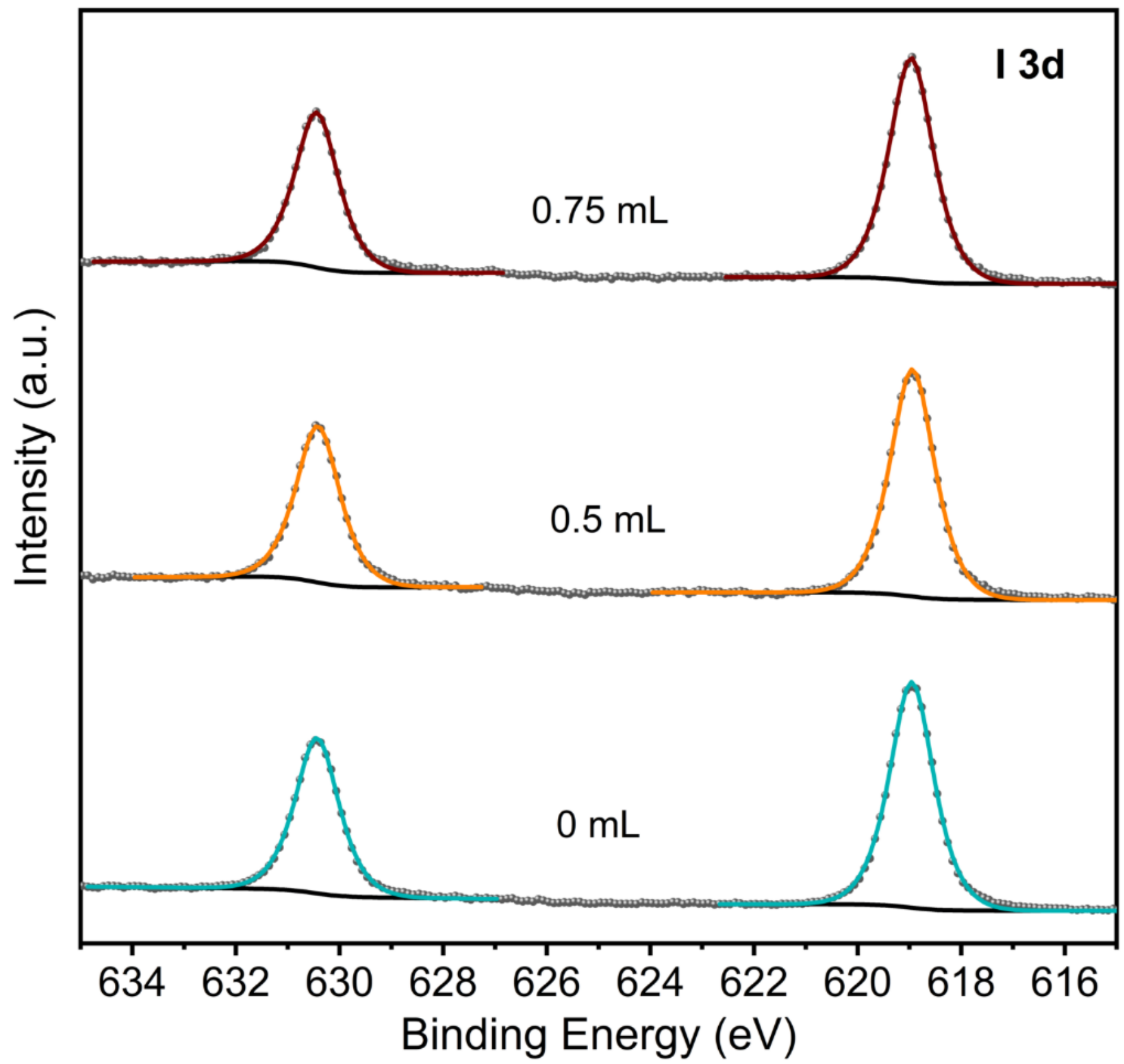

Figure S12: Comparison between I 3d XPS spectra of 0 mL, 0.5 mL and 0.75 mL TDP-I-treated $CsPbI_3$ NCs. The I 3d spectra remain nearly unchanged after TDP-I treatment, with no additional iodine species or noticeable binding-energy shifts observed. These results indicate that the iodide framework of $CsPbI_3$ is well preserved during the ligand-exchange process.

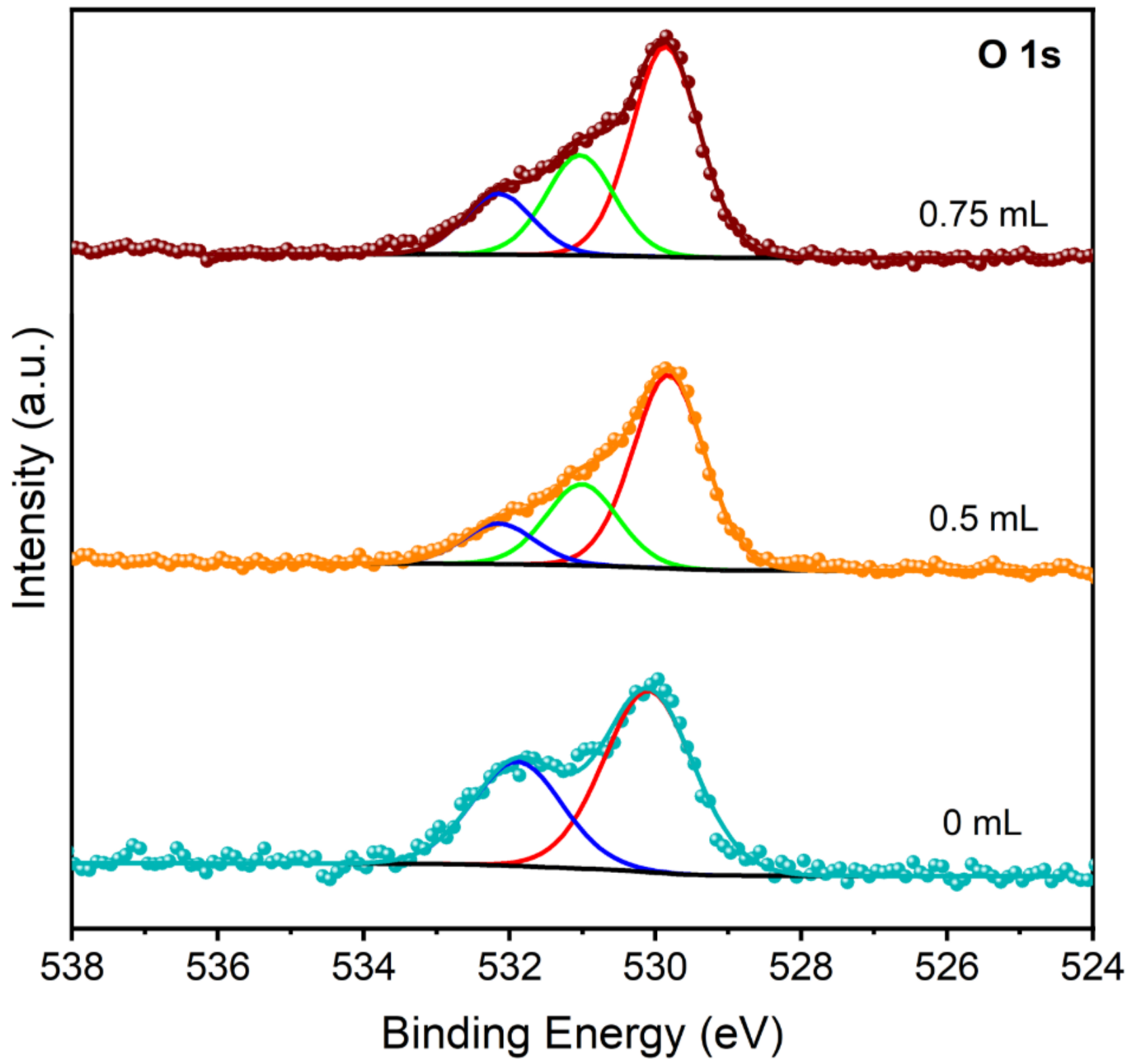

Figure S13: Comparison between O 1s XPS spectra of 0 mL, 0.5 mL and 0.75 mL TDP-I-treated $CsPbI_3$ NCs. The O 1s spectra can be deconvoluted into two or three components corresponding to metal-coordinated carboxylate oxygen (~530 eV) and weakly bound oxygen-containing species (~531 eV and ~532 eV). With increasing TDP-I treatment, the high-binding-energy component gradually decreases, indicating the removal of loosely bound oleate species and supporting the surface ligand-exchange process.

Table S1: Quantitative elemental analysis of 0 mL, 0.5 mL and 0.75 mL TDP-I-treated $CsPbI_3$ NCs through XPS.

Samples	Cs (%)	Pb (%)	I (%)	N (%)	Cs/Pb	I/Pb
0 mL	14.9	16.9	57.2	11.0	1.13	3.38
0.5 mL	15.1	17.1	56.2	11.6	1.13	3.29
0.75 mL	16.2	16.9	55.0	11.9	1.04	3.25

Table S2: N/P ratio analysis of 0 mL, 0.5 mL and 0.75 mL TDP-I-treated $CsPbI_3$ NCs through XPS.

Samples	N (%)	P (%)	N/P
0 mL	□	□	□
0.5 mL	74.5	25.5	2.92
0.75 mL	65.9	34.1	1.93

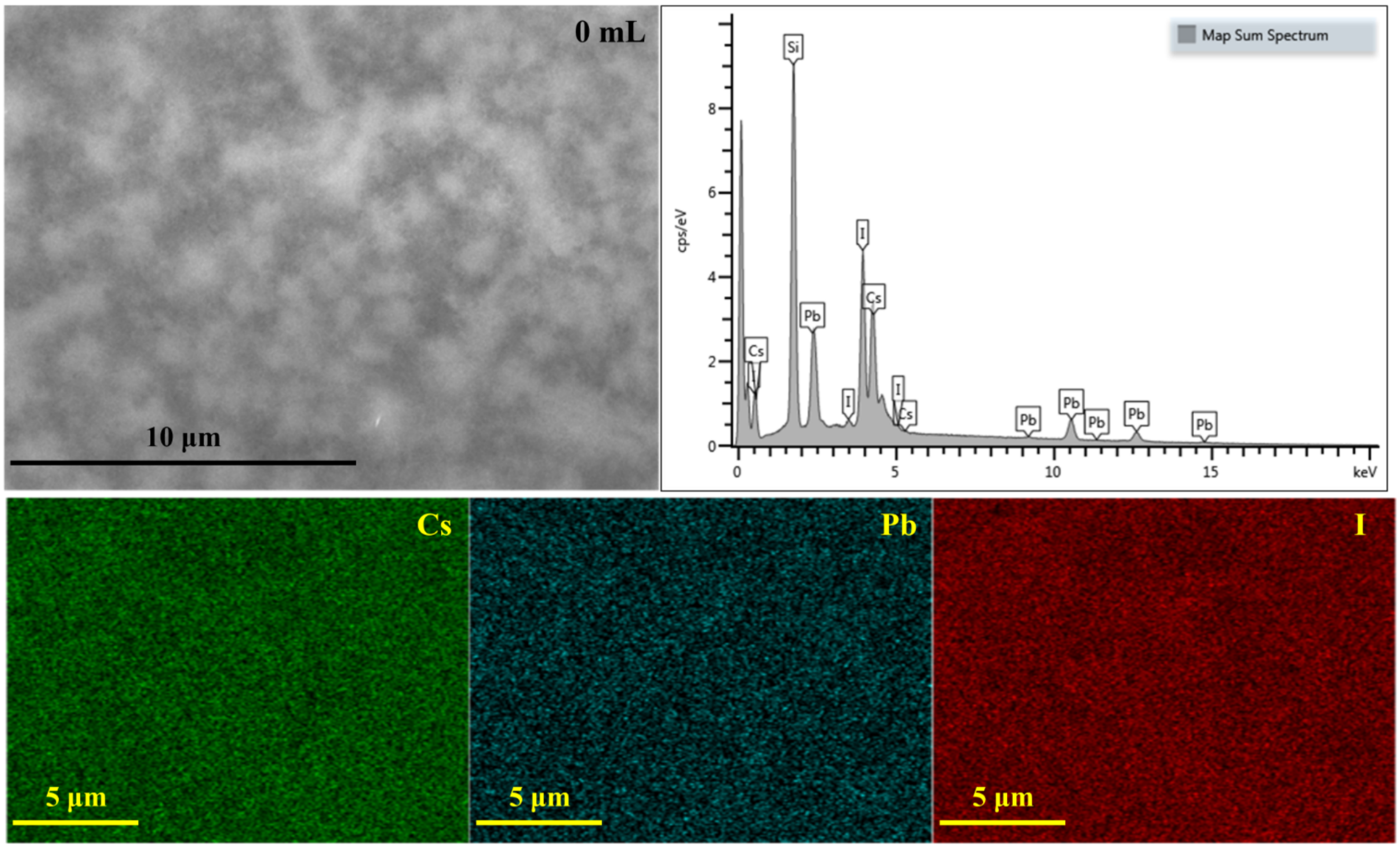

Figure S14: SEM-EDS elemental mapping of pristine $CsPbI_3$ NCs.

Table S3: Elemental analysis of the area shown in Figure S14 derived from SEM-EDS.

Map Sum Spectrum	Line Type	W_t %	W_t % Sigma	Atomic %
Cs	L series	12.89	0.17	13.72
Pb	L series	25.08	0.22	17.13

I	L series	62.03	0.23	69.16
P	K series	0	0	0
Total		100.00		100.00

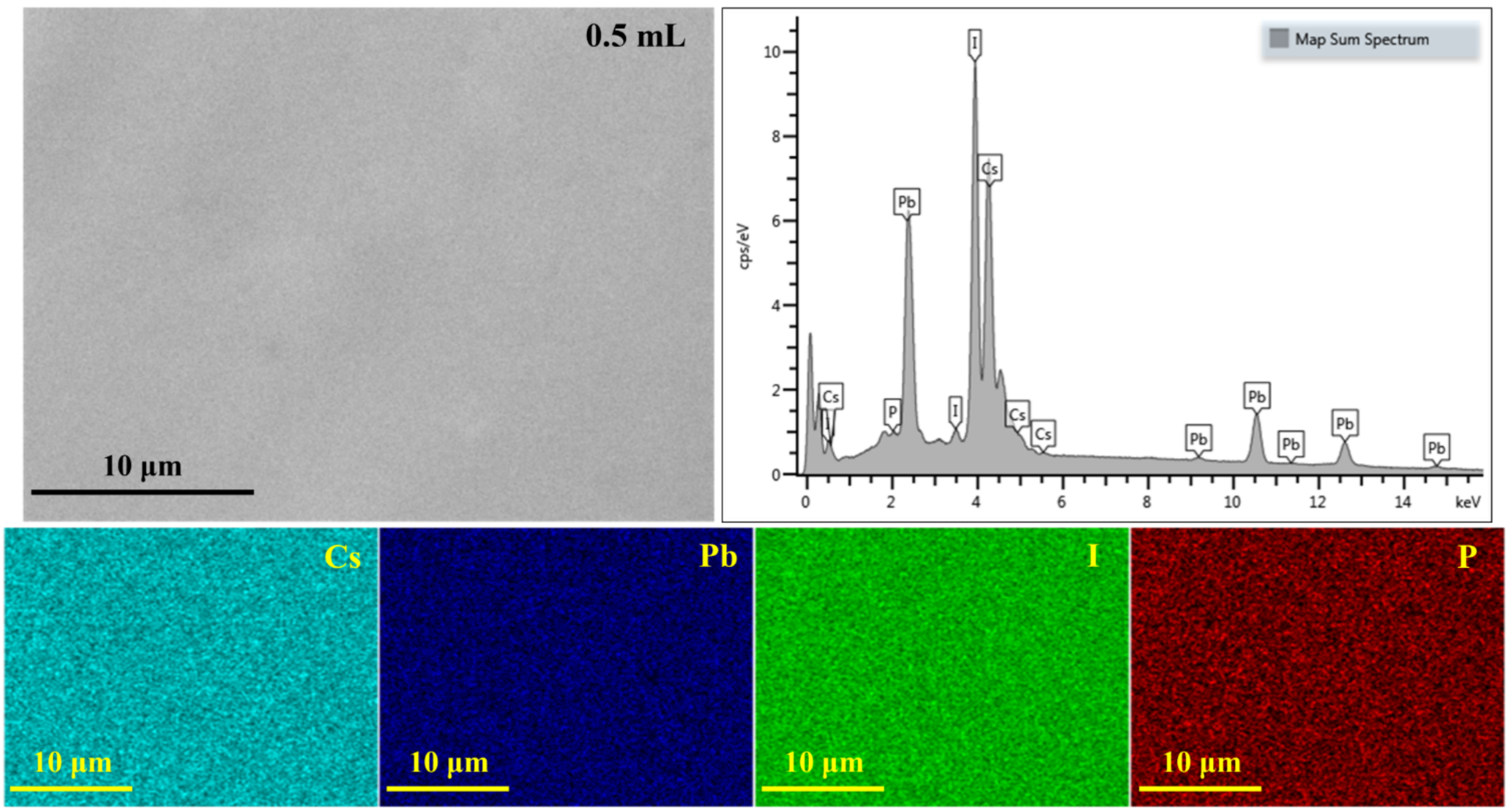

Figure S15: SEM-EDS elemental mapping of 0.5 mL TDP-I-treated $CsPbI_3$ NCs.

Table S4: Elemental analysis of the area shown in Figure S15 derived from SEM-EDS.

Map Sum Spectrum	Line Type	W_t %	W_t % Sigma	Atomic %

Cs	L series	13.28	0.09	13.90
Pb	L series	26.93	0.12	18.08
I	L series	59.06	0.13	64.74
P	K series	0.73	0.03	3.28
Total		100.00		100.00

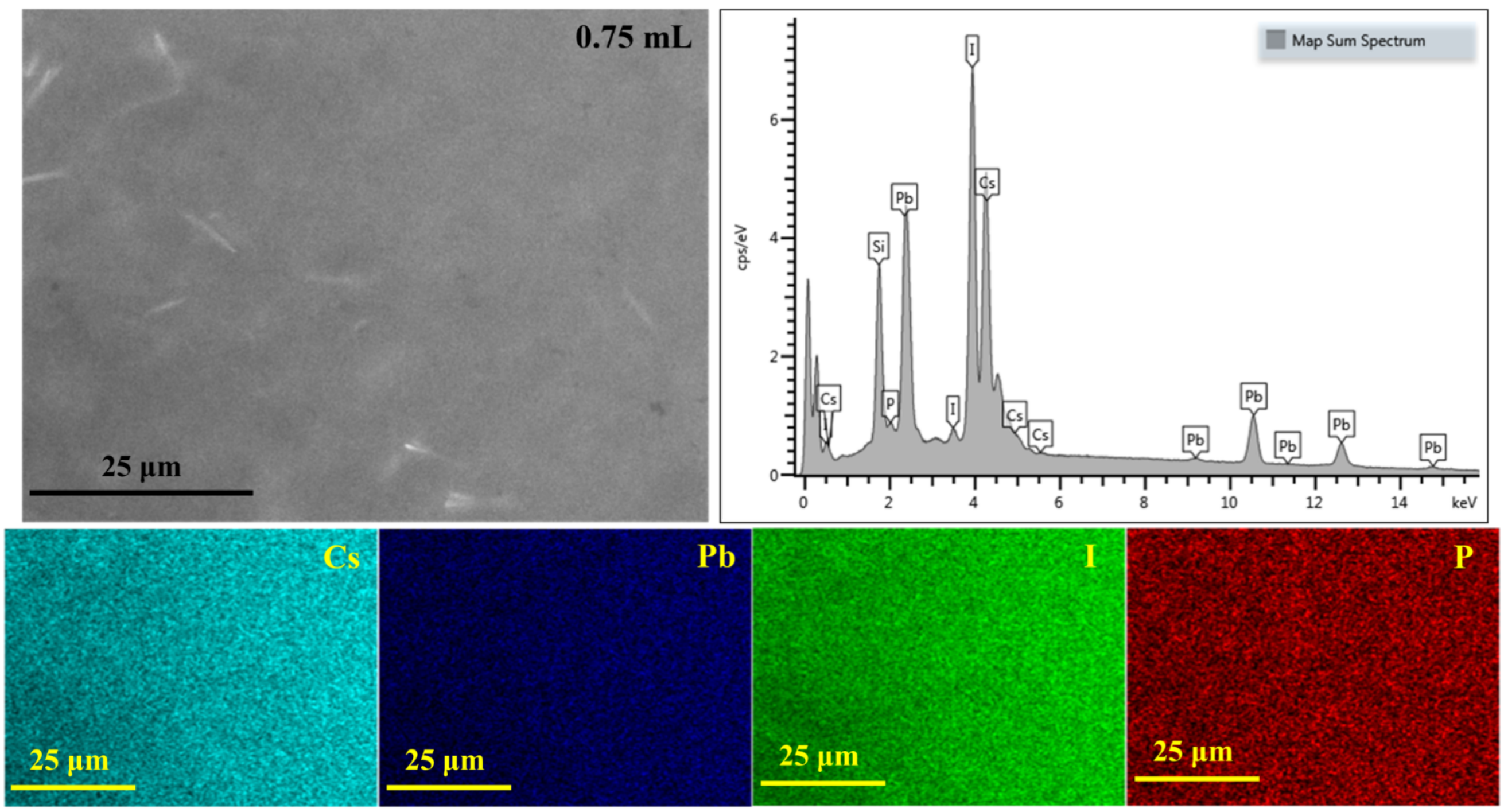

Figure S16: SEM-EDS elemental mapping of 0.75 mL TDP-I-treated $CsPbI_3$ NCs.

Table S5: Elemental analysis of the area shown in Figure S16 derived from SEM-EDS.

Map Sum Spectrum	Line Type	W_t %	W_t % Sigma	Atomic %
Cs	L series	12.28	0.10	12.66
Pb	L series	26.87	0.13	17.77
I	L series	59.70	0.13	64.45
P	K series	1.16	0.04	5.12
Total		100.00		100.00

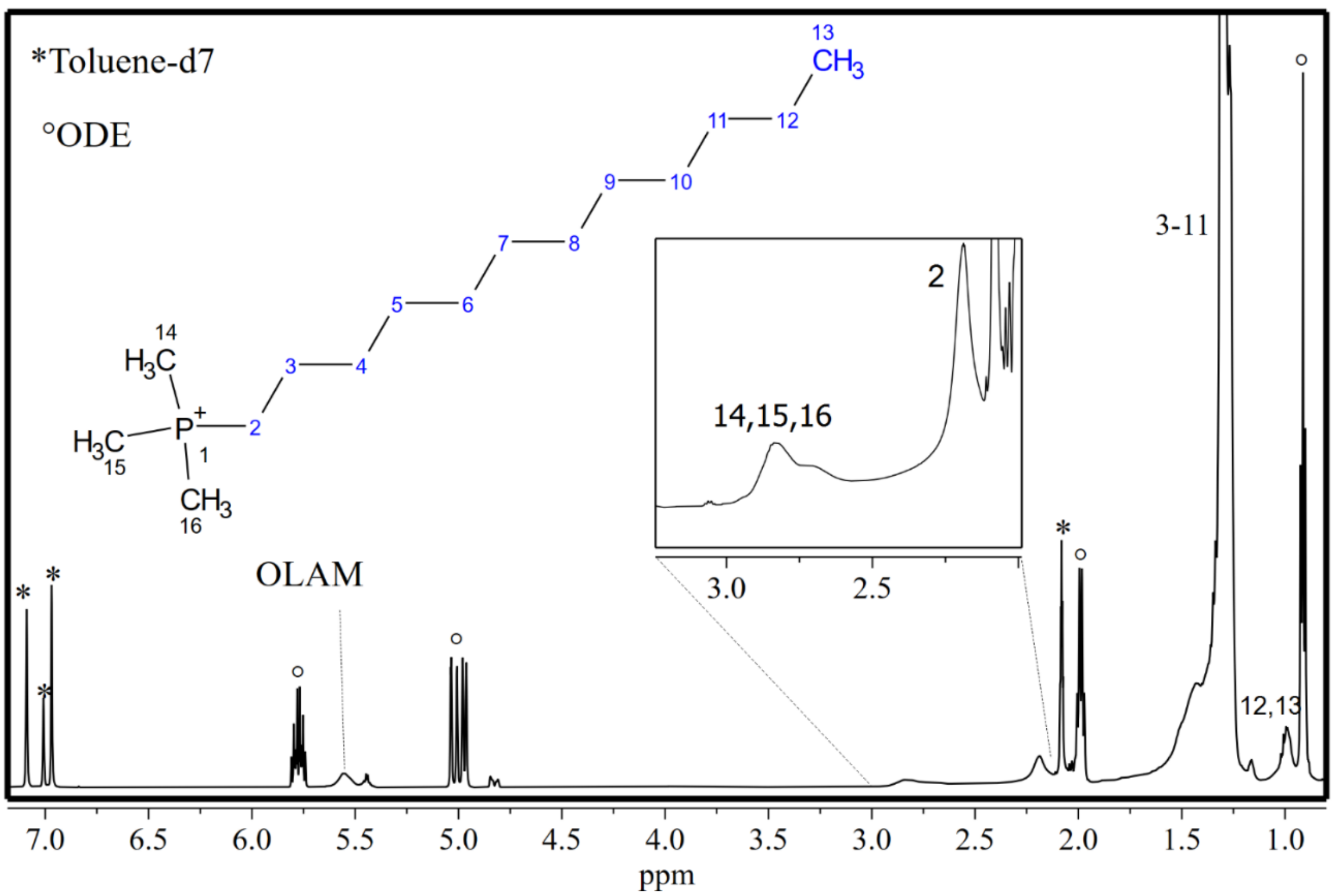

Figure S17: ^{1}H NMR spectrum of TDP-I-capped $CsPbI_3$ NCs in toluene-D. The diagnostic signals of TDP bound to the $CsPbI_3$ NC's surface are assigned in the spectrum. The structure formula and peak numbering are embedded. The residual OLAM double bound signal is indicated in the figure. The peaks of ligand which

interact with the NC's surface are broad without a clear peak multiplicity caused by and increased correlation time (τ$_C$).

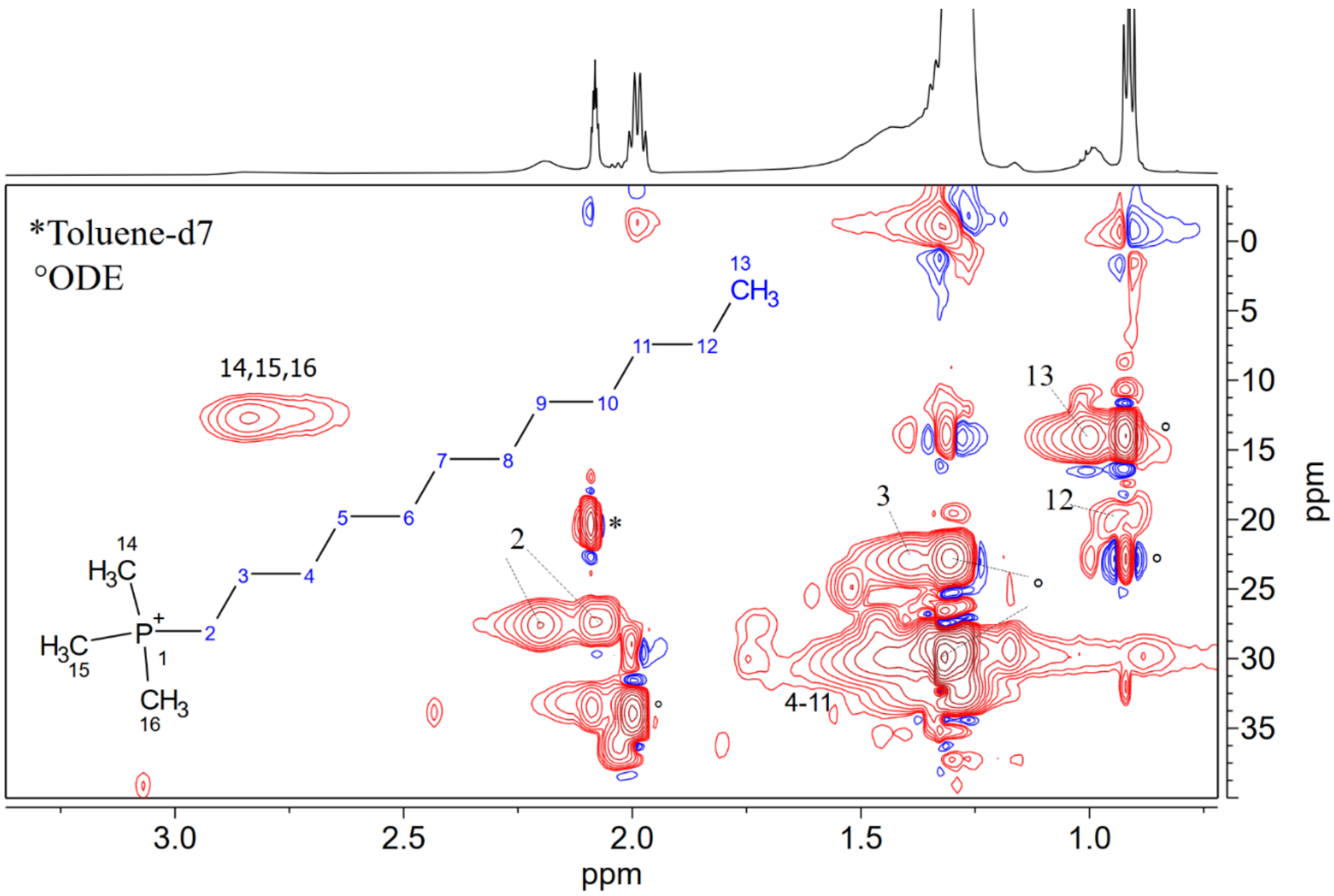

Figure S18: ^{1}H-^{13}C HSQC NMR experiment of TDP-I-capped $CsPbI_3$ NCs in toluene-D. The diagnostic signals of TDP bound to the $CsPbI_3$ NC's surface are assigned in the spectrum. The structure formula with peak numbering is embedded.

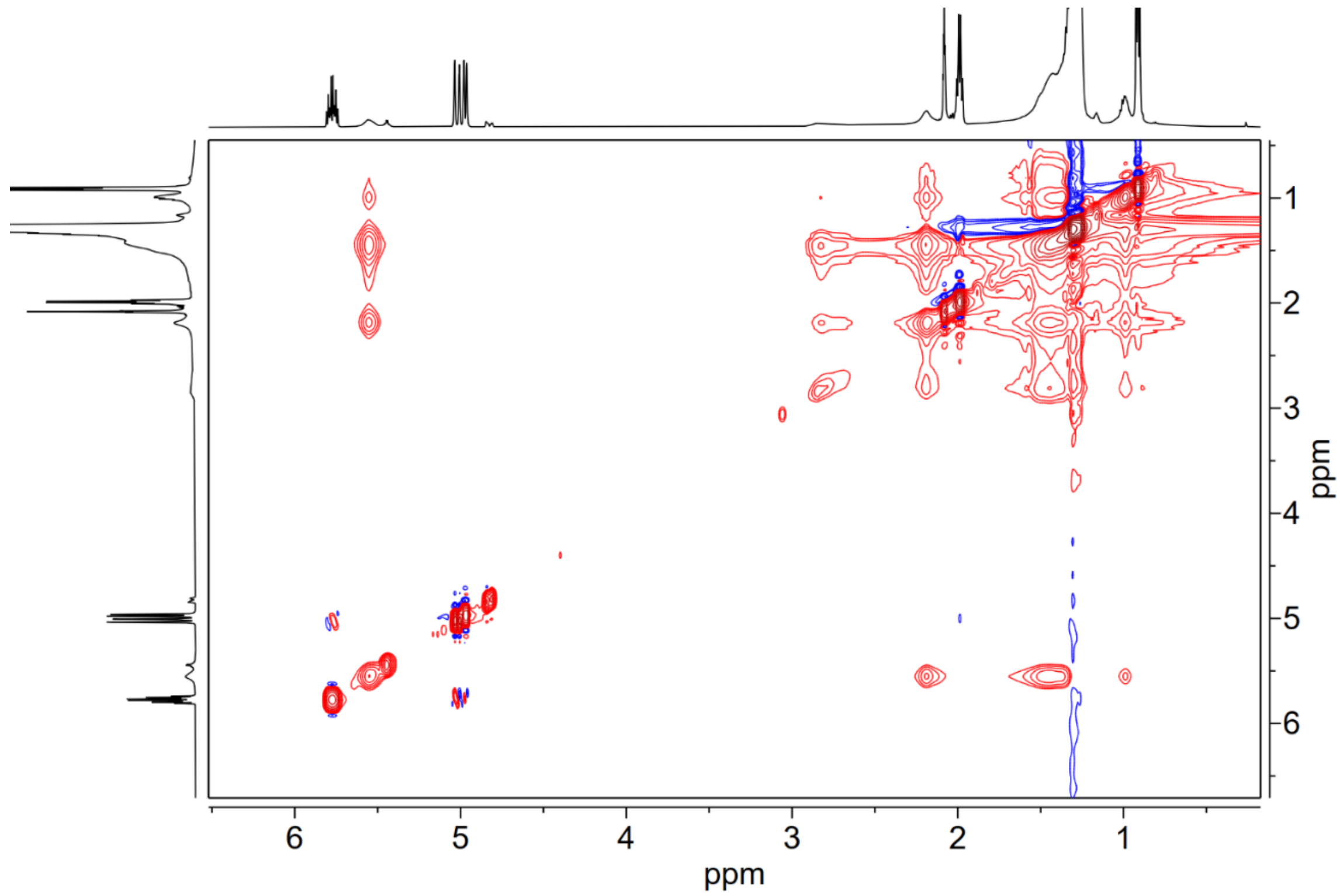

Figure S19: ^{1}H-^{1}H NOESY NMR experiment of TDP-I-capped $CsPbI_3$ NCs in toluene-D. The negative (red) NOE cross peaks are typical of species with long correlation time (τ_C) in solution, due to the binding with the NC's surface. Both OLAM and TDP dynamically interact with NC's surface by assuming the slow tumbling regime of the NC.

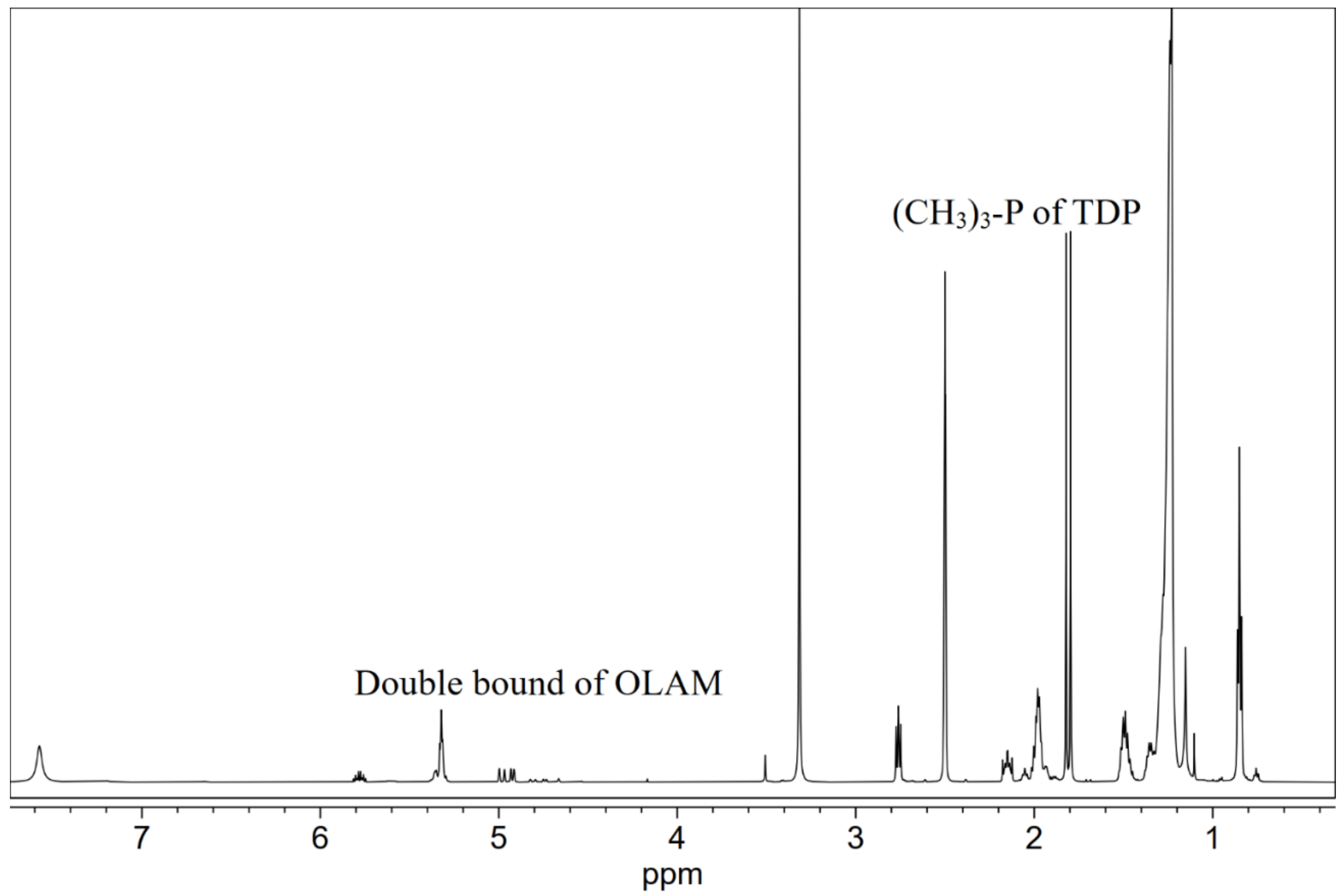

Figure S20: Absolute quantitative ¹H NMR spectrum of TDP-I ligand in DMSO-d6, ligand concentration is determined by comparing diagnostic integrated ligand peaks to that of a 10 mM standard dimethyl sulfone solution by using the PULCON method. Diagnostic peaks of ligands used for quantification are labeled in the figure.

Table S6: Summary of the parameters employed for the ligand-shell composition analysis.

	Pristine $CsPbI_3$ NCs		0.75 mL TDP-I-treated $CsPbI_3$ NCs		
**Ligands**	**Oleate**	**OLAm**	**Oleate**	**OLAm**	**TDP**
**[Pb] - ICP-OES (M)**	4.38E-05	4.38E-05	1.08E-05	1.08E-05	1.08E-05
**[Pb]-DMSO (M)**	4.38E-02	4.38E-02	5.41E-03	5.41E-03	5.41E-03
**Nº Pb atoms**	5.28E+18	5.28E+18	6.52E+17	6.52E+17	6.52E+17
**NCs size (nm)**	12	12	8	8	8
**NCs volume (nm^3)**	1728	1728	512	512	512
**a**	0.8856	0.8856	0.8856	0.8856	0.8856
**b**	1.2472	1.2472	1.2472	1.2472	1.2472
**c**	0.8577	0.8577	0.8577	0.8577	0.8577
**Unit cell volume (nm^3)**	9.47E-01	9.47E-01	9.47E-01	9.47E-01	9.47E-01
**Unit cells/NCs**	1.82E+03	1.82E+03	5.40E+02	5.40E+02	5.40E+02
**Nº Pb atoms/NC**	7.30E+03	7.30E+03	2.16E+03	2.16E+03	2.16E+03
**Nº NCs - DMSO sample**	7.23E+14	7.23E+14	3.01E+14	3.01E+14	3.01E+14
**[Ligands] (M)**	8.17E-04	1.01E-02	5.13E-04	2.03E-03	1.22E-03
**Nº ligand molecules**	9.84E+16	1.22E+18	6.18E+16	2.44E+17	1.46E+17
**Surface ligand (%)**	7.456420553	92.54357945	13.65451158	54.00585574	32.33963269

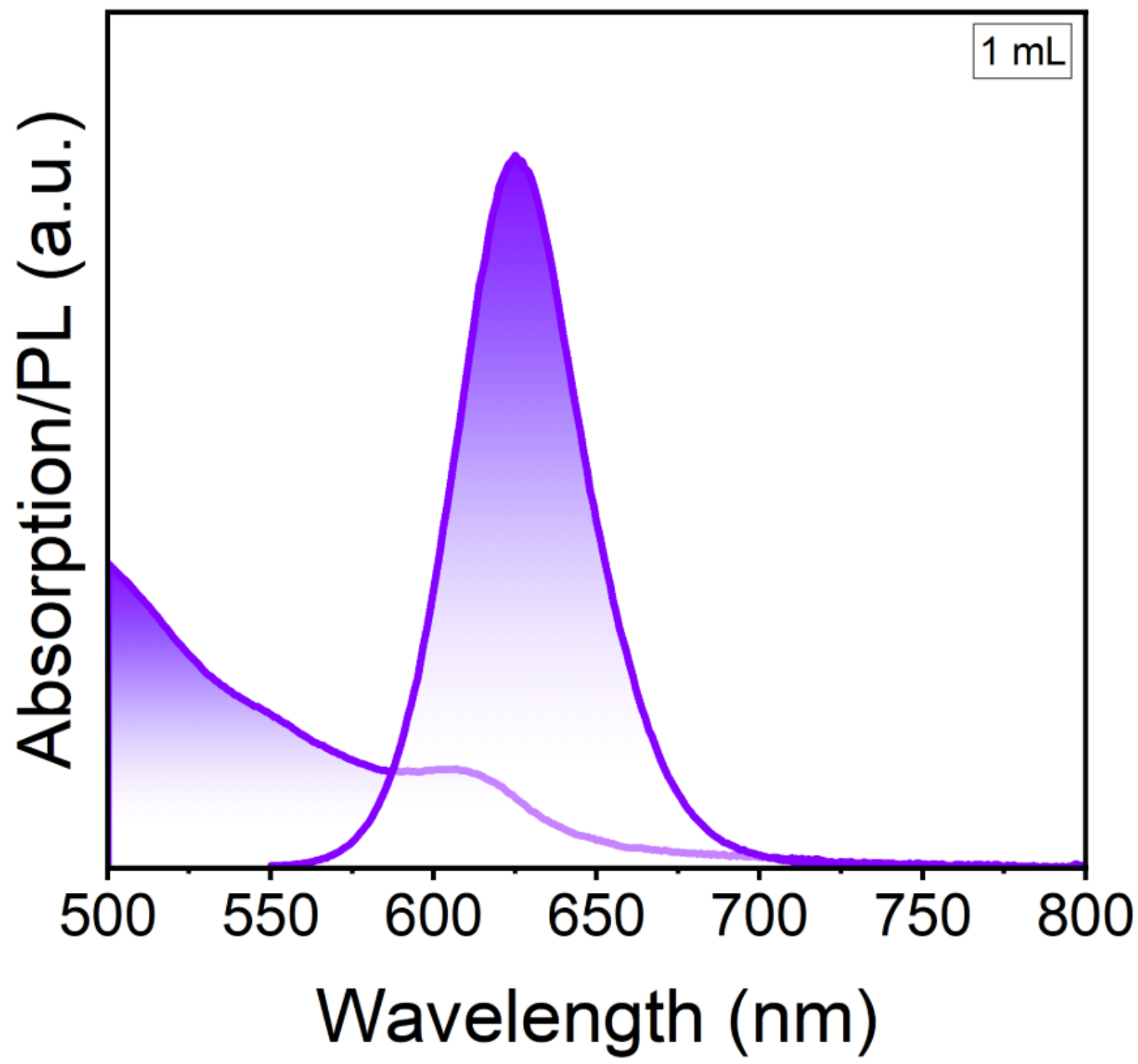

Figure S21: UV-visible absorption and PL spectra of 1 mL TDP-I-treated $CsPbI_3$ NCs solution.

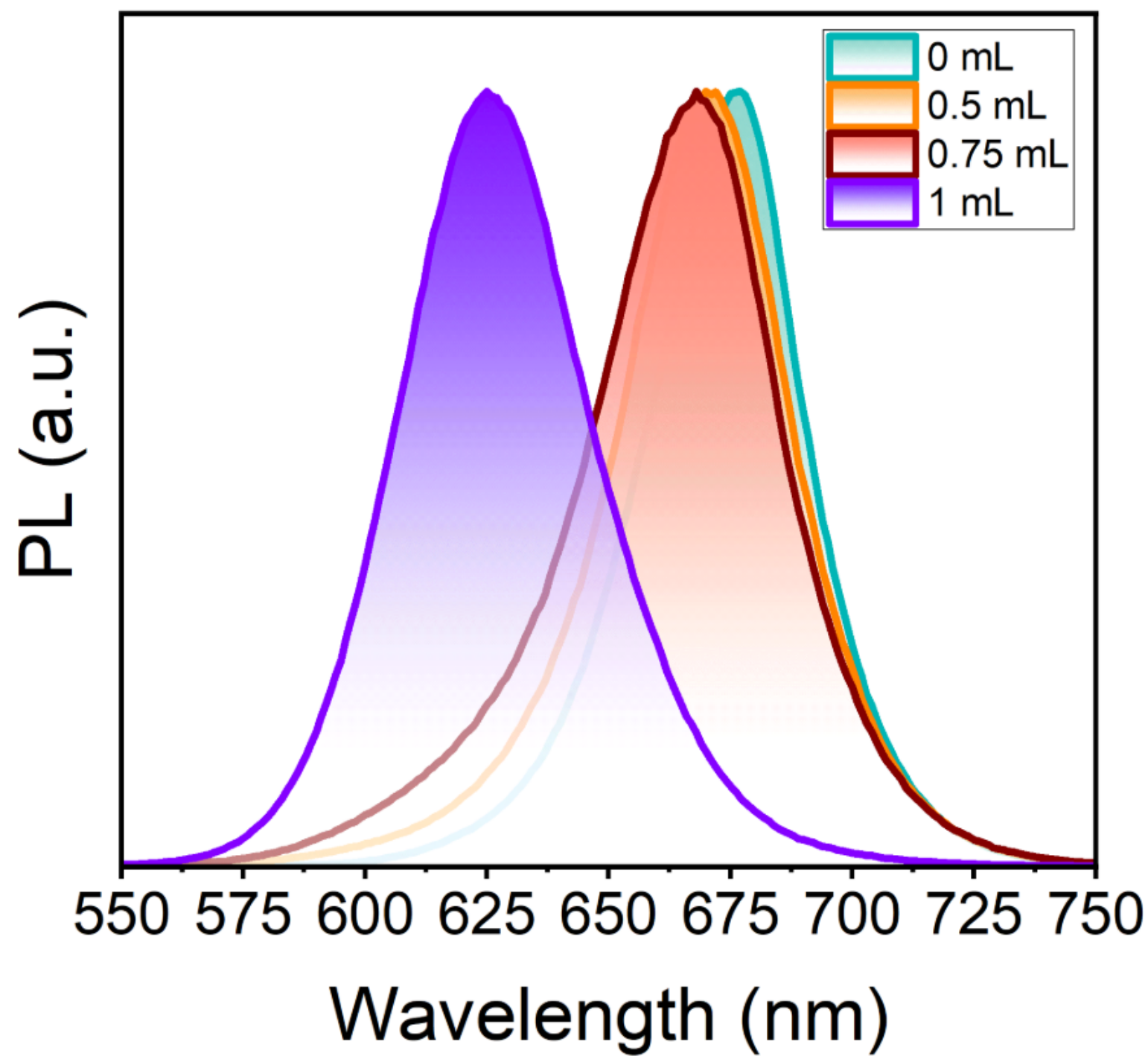

Figure S22: PL spectra shift of 0 mL, 0.5 mL, 0.75 mL and 1 mL TDP-I-treated $CsPbI_3$ NCs solution.

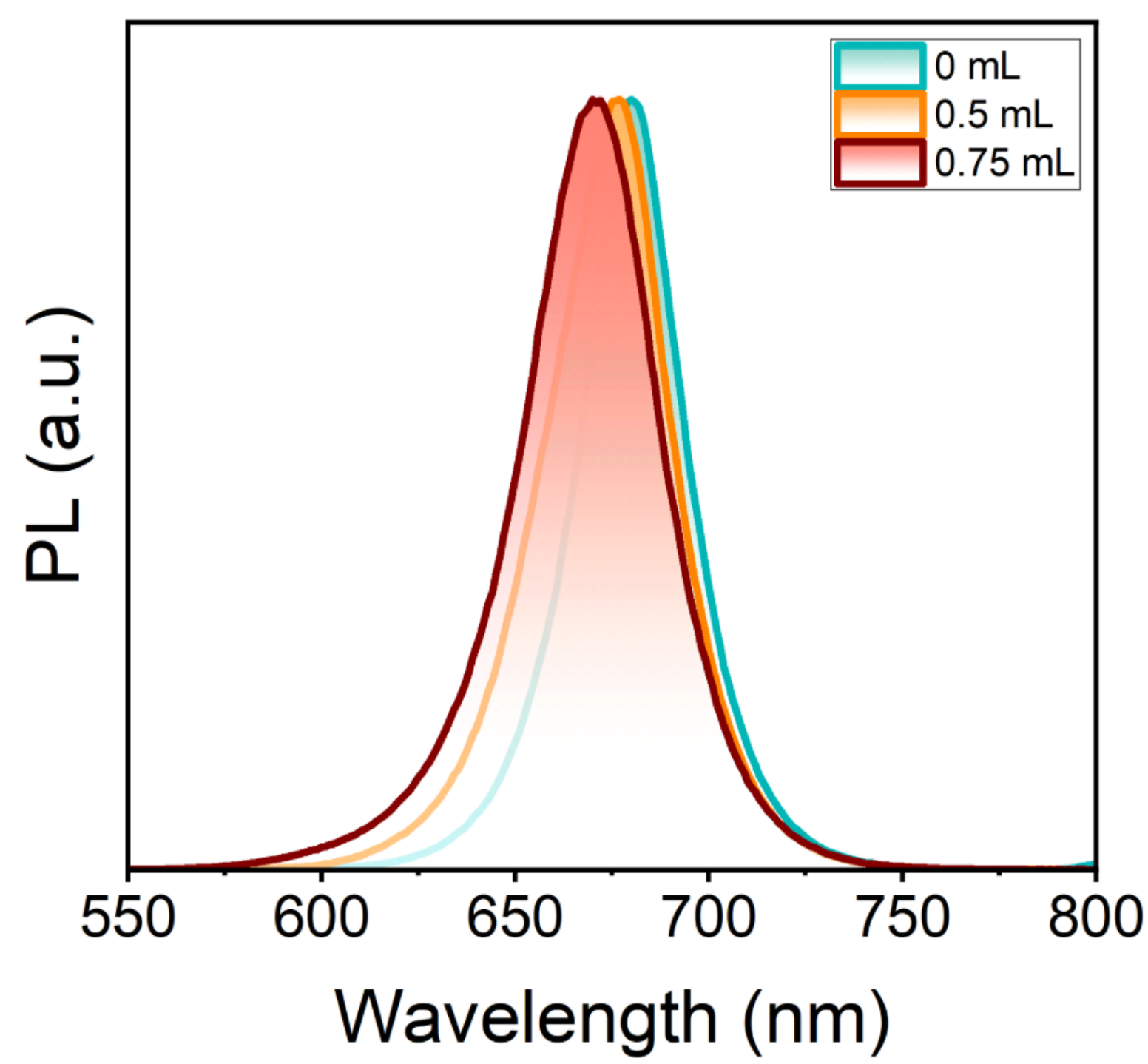

Figure S23: PL spectra of 0 mL, 0.5 mL and 0.75 mL TDP-I-treated $CsPbI_3$ NCs thin film.

Table S7: TRPL fitting parameters and average PL lifetime ($τ_{ave}$) of 0 mL, 0.5 mL and 0.75 mL TDP-I-treated $CsPbI_3$ NCs.

Samples	$τ_1$ (ns)	A_1 (%)	$τ_2$ (ns)	A_2 (%)	$τ_{avg}$ (ns)	$χ^2$

0 mL TDP-I	10.77	79.33	26.55	20.67	16.94	1.15
0.5 mL TDP-I	14.05	71.72	34.81	28.28	24.30	1.20
0.75 mL TDP-I	17.97	62.6	33.86	37.40	26.38	1.18

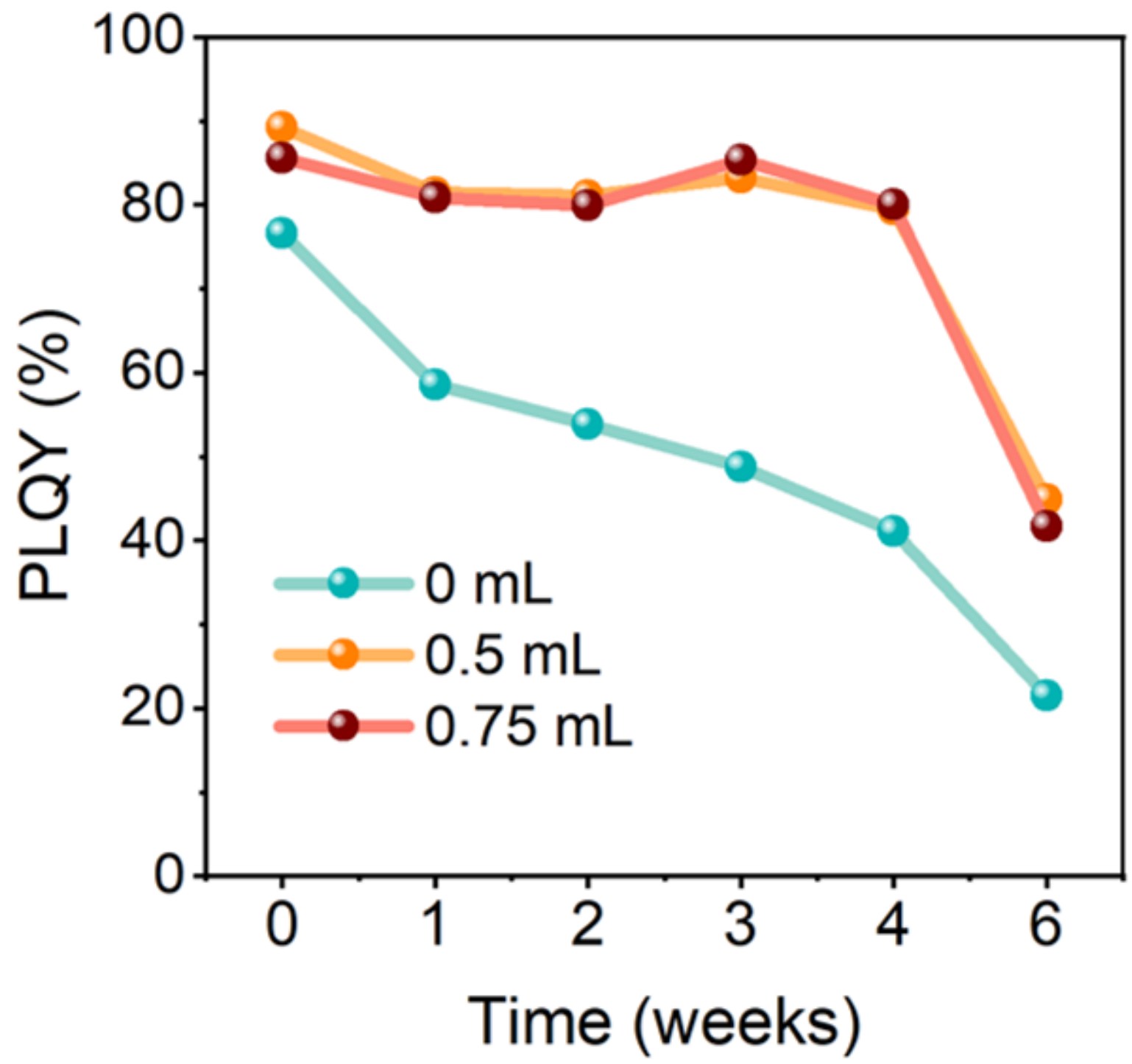

Figure S24: PLQY stability of 0 mL, 0.5 mL and 0.75 mL TDP-I-treated $CsPbI_3$ NCs in anhydrous toluene over time in the air.

Table S8: Calculated Force-Field Parameters for the $CsPbI_3$ NC Core and the Ligand-Capped NC. Charges (q) are provided in elementary charge units, while the distances (σ) are reported at nm.

	OLAm	TDP-I
	**Charges**	
**Cs**	0.754	0.612
**Pb**	1.630	1.231
**I**	-0.786	-0.614
**N**	-0.867	-
**H1 (Ammonium hydrogen)**	0.386	-
**P**	-	1.171
**C (P-bonded)**	-	-0.222
**H2 (P-bonded)**	-	0.029
	**Sigma σ**	
**Cs Cs**	0.576	0.4679257
**Cs I**	0.408	0.4008943
**Cs Pb**	0.457	0.4194387
**I I**	0.411	0.4302504
**I Pb**	0.345	0.3338201
**Pb Pb**	0.722	0.6811604
**Cs N**	0.496	-
**I N**	0.348	-
**Pb N**	0.369	-
**Cs H1**	0.442	-
**I H1**	0.272	-
**Pb H1**	0.375	-
**Cs P**	-	0.3859106
**I P**	-	0.4041482
**Pb P**	-	0.505
**Cs C**	-	0.371095

**I C**	-	0.3978751
**Pb C**	-	0.3842497
**Cs H2**	-	0.3312183
**I H2**	-	0.3057185
**Pb H2**	-	0.3214184

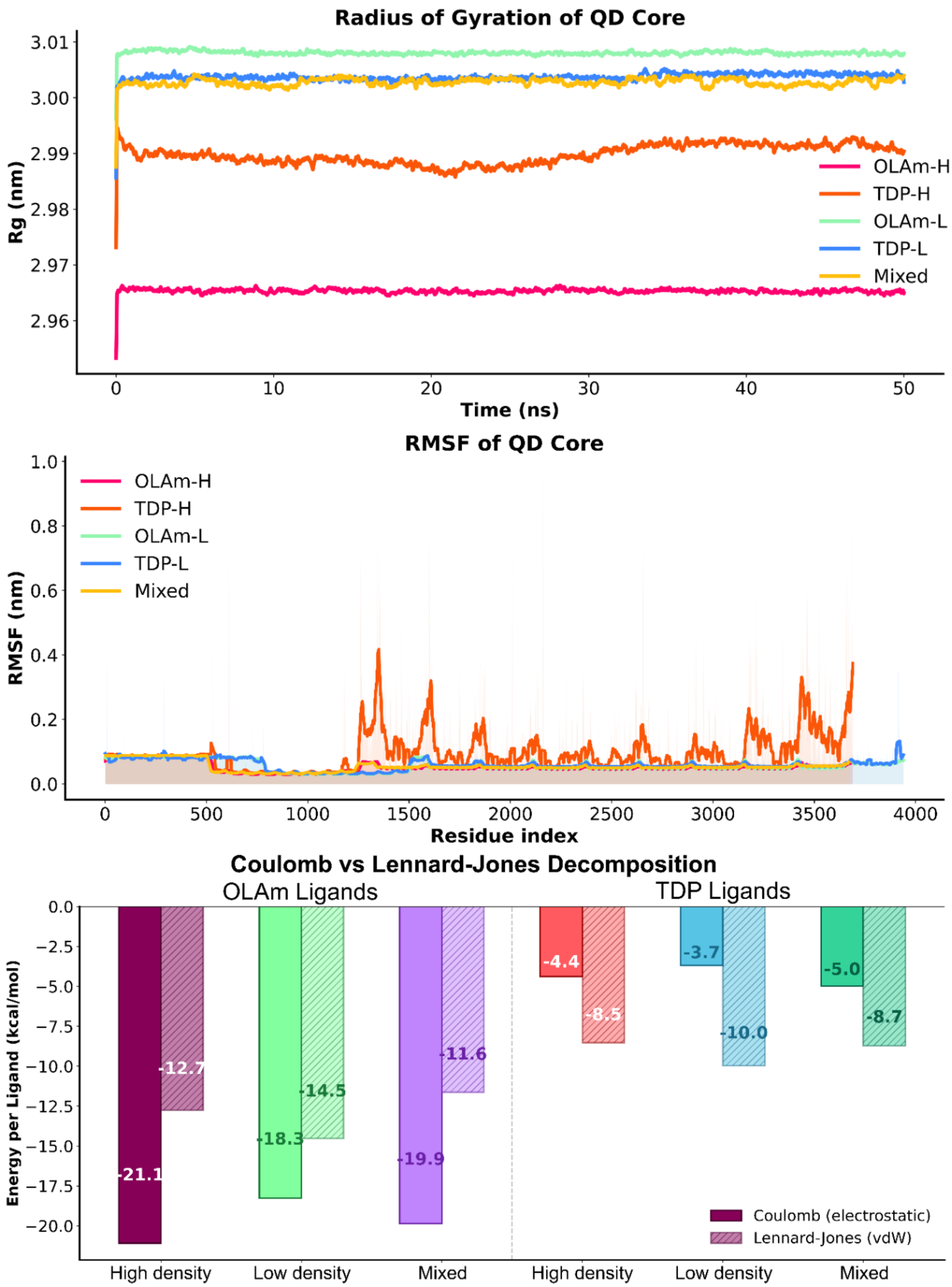

Figure S25: (Top) Time evolution of the radius of gyration (Rg) of the NC inorganic core over the 50 ns production trajectory, reflecting the overall structural compactness of each system. (Middle) Root-mean-square

fluctuation (RMSF) of the NC inorganic core atoms as a function of residue index, quantifying the time-averaged atomic displacement from the mean position over the full production trajectory. (Bottom) Decomposition of the per-ligand ligand–core interaction energy into short-range Coulomb (electrostatic, solid bars) and Lennard-Jones (dispersive/vdW, hatched bars) contributions for OLAm and TDP-I across all surface coverage models.

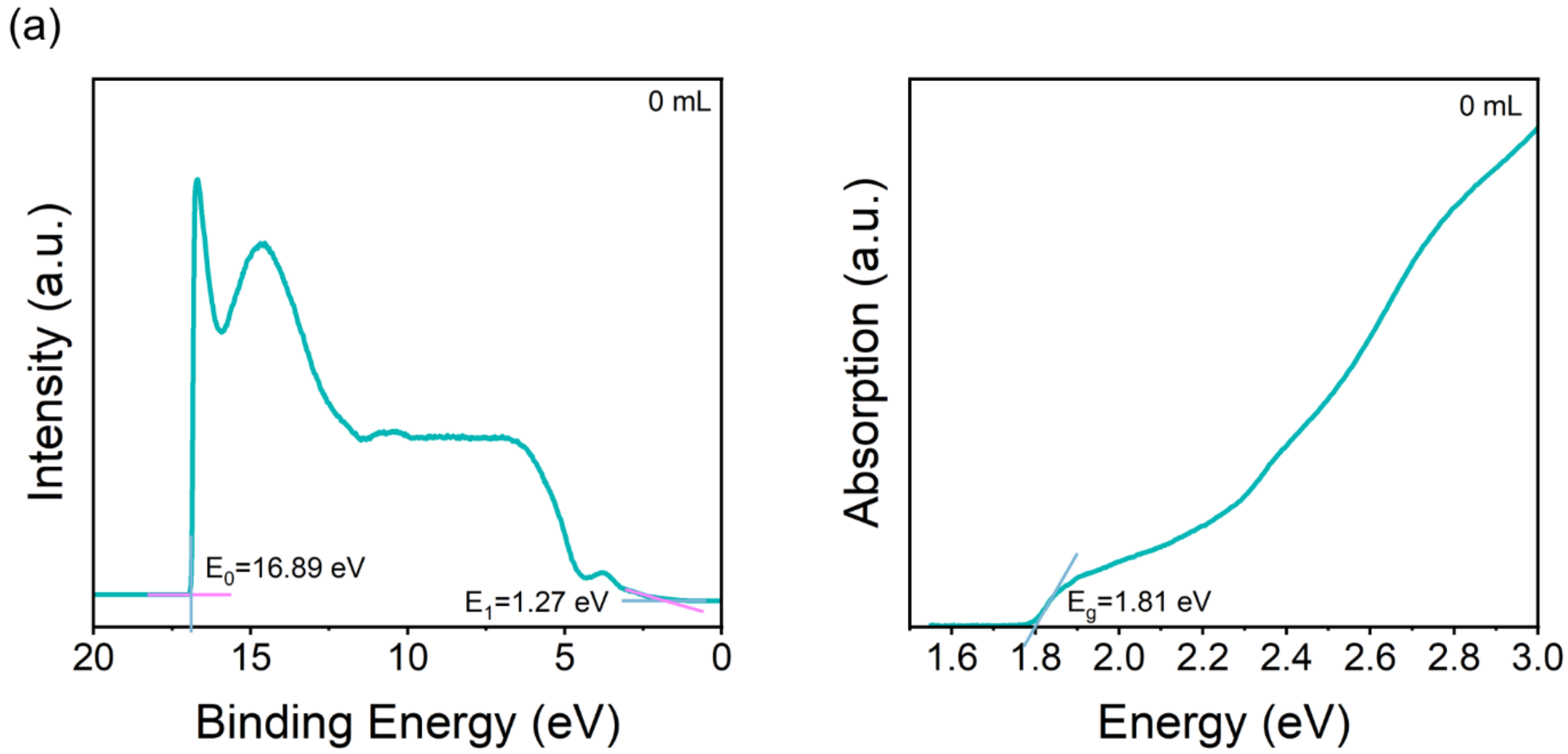

Figure S26: (a) UPS spectrum, and (b) optical absorption spectrum of pristine $CsPbI_3$ NCs.

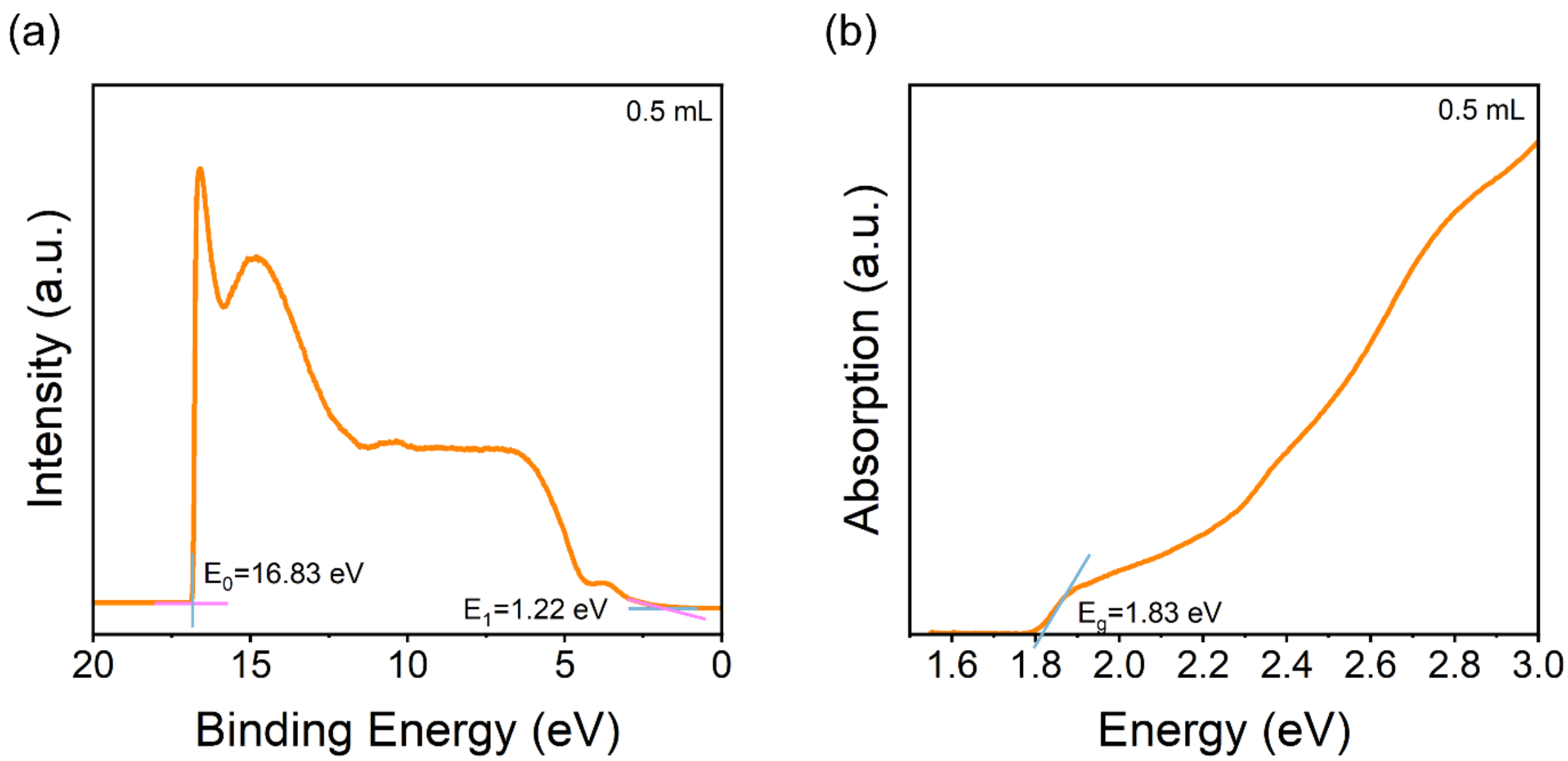

Figure S27: UPS spectrum and absorption spectrum of 0.5 mL TDP-I-treated $CsPbI_3$ NCs.

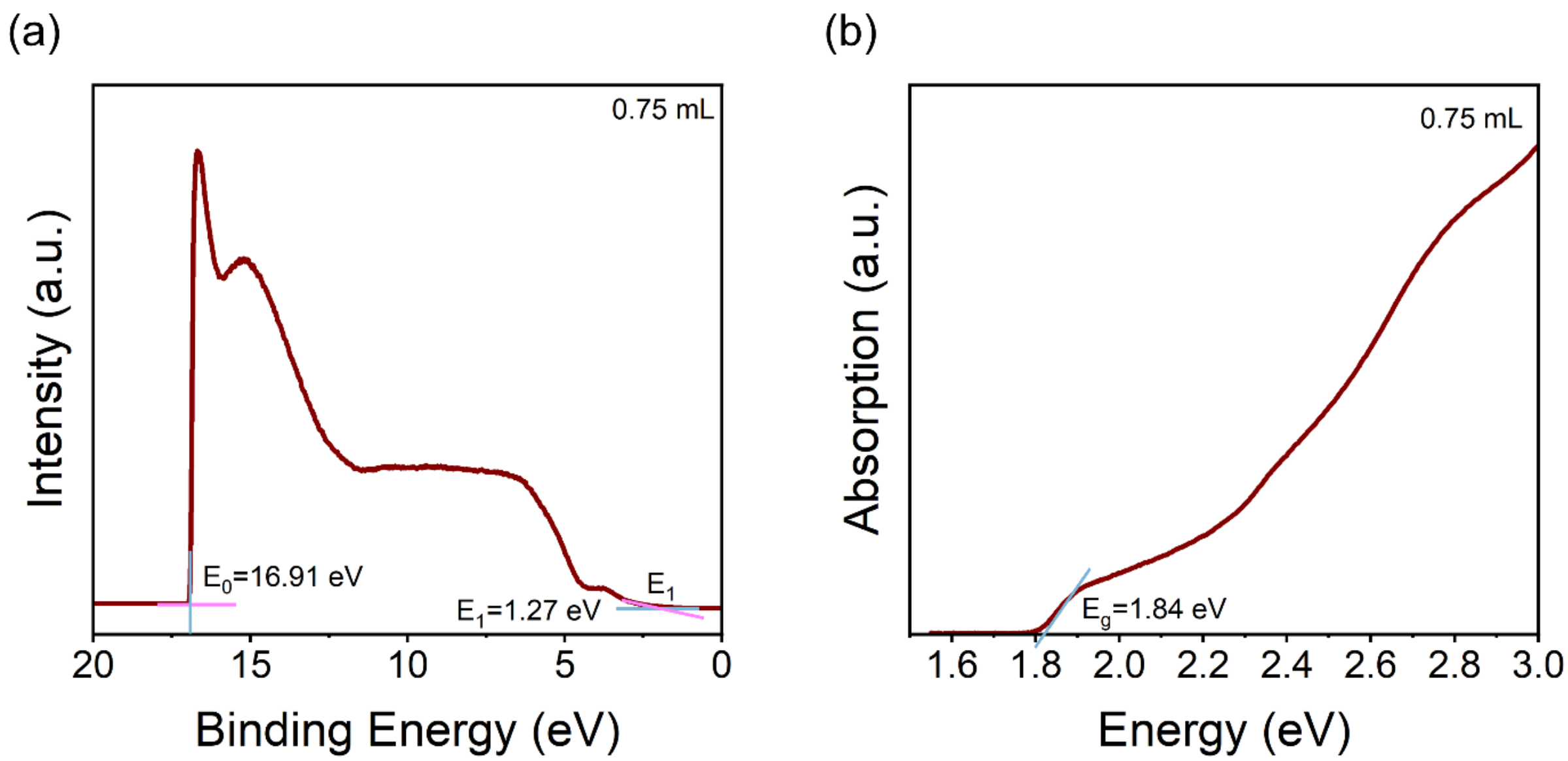

Figure S28: UPS spectrum and absorption spectrum of 0.75 mL TDP-I-treated $CsPbI_3$ NCs.

Table S9: UPS analysis of 0 mL, 0.5 mL and 0.75 mL TDP-I-treated $CsPbI_3$ NCs.

	WF	E_{ion}	$E_1=E_{ion}$-WF
0 mL	4.33	5.60	1.27
0.5 mL	4.39	5.61	1.22

0.75 mL	4.31	5.58	1.27

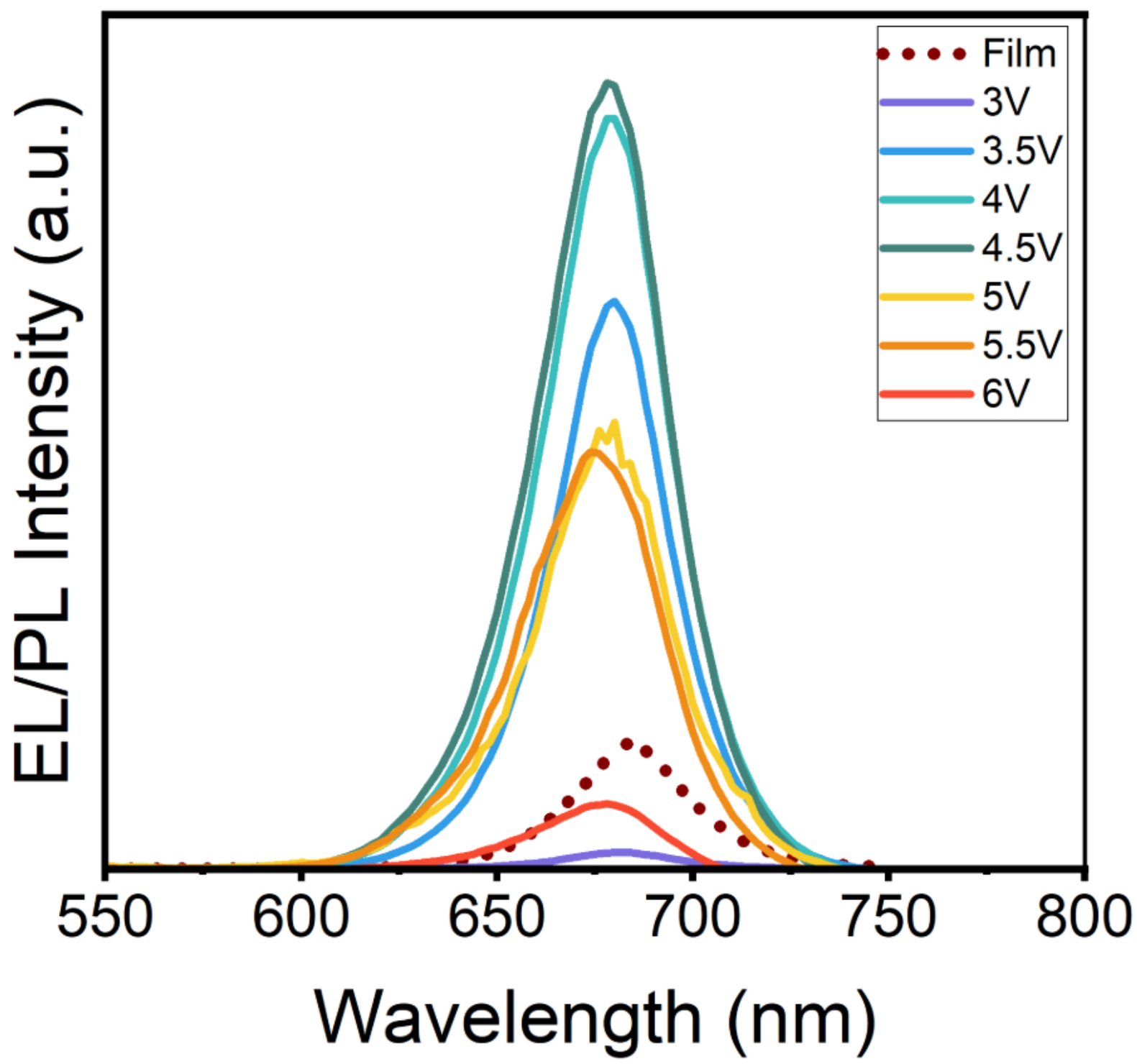

Figure S29: EL spectra of the pristine (0 mL) NCs based device under different applied voltages.

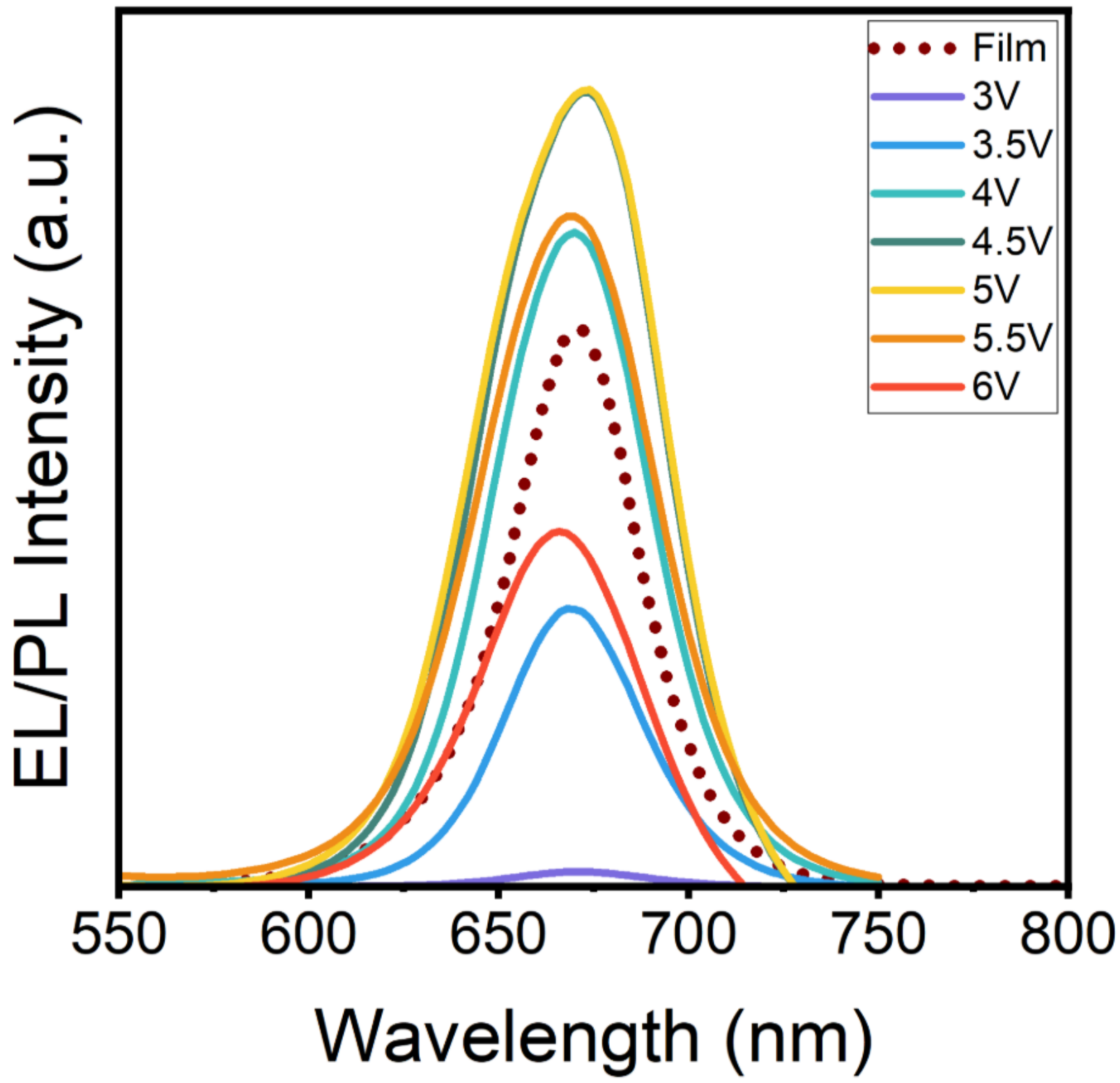

Figure S30: EL spectra of the 0.5 mL TDP-I-treated device under different applied voltages.

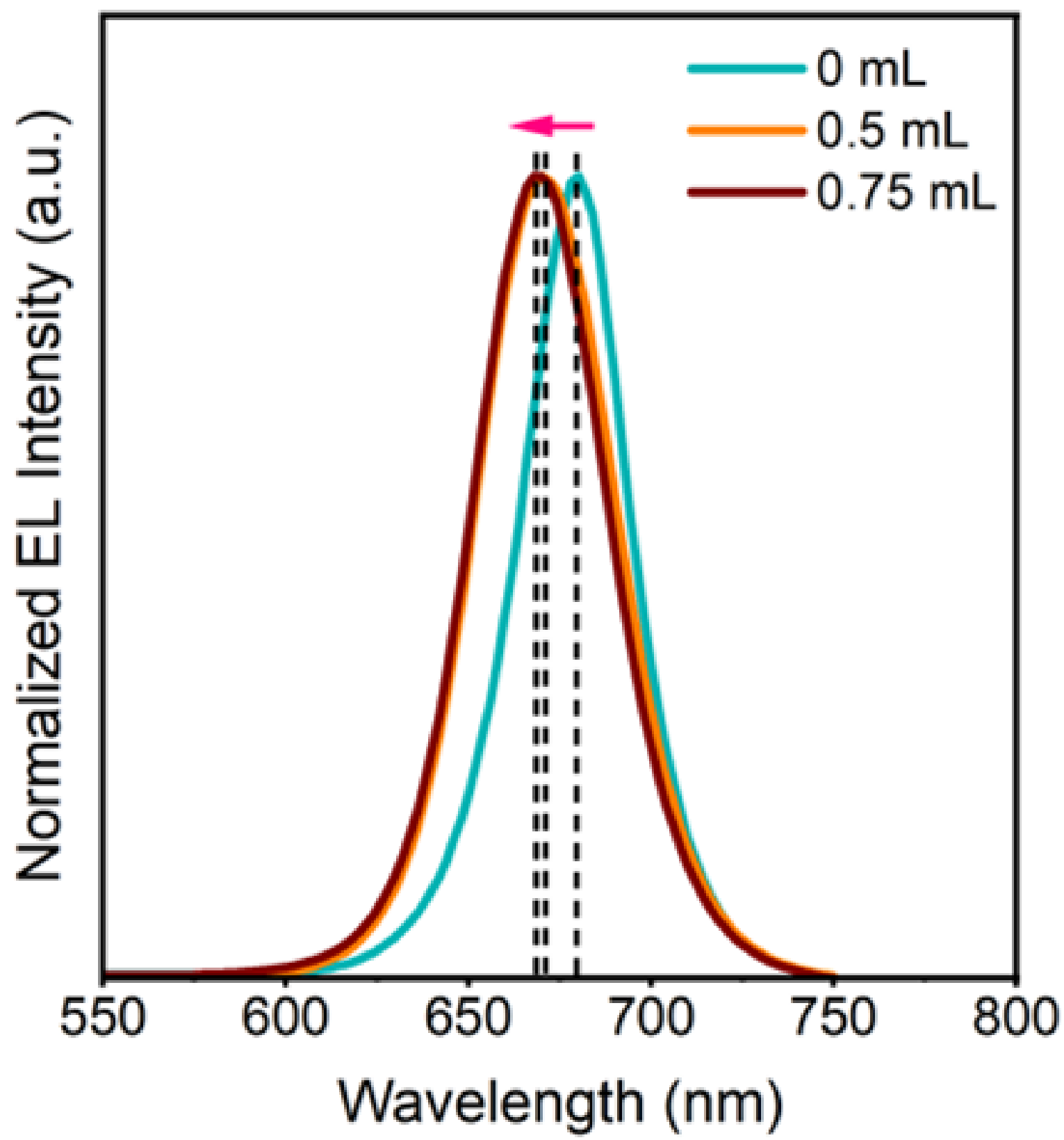

Figure S31: EL spectra shift of 0 mL, 0.5 mL and 0.75 mL TDP-I-treated $CsPbI_3$ NCs devices.

Table S10: Optical properties of 0 mL, 0.5 mL and 0.75 mL TDP-I-treated $CsPbI_3$ NCs films and devices.

	PL (nm)	PL fwhm (nm)	PLQY (%)	EL (nm)	EL fwhm (nm)	EQE (%)
0 mL TDP-I	680	35	41 ± 4	679	41	4.97
0.5 mL TDP-I	675	41	49 ± 5	672	50	10.50
0.75 mL TDP-I	671	44	47 ± 5	669	49	12.40

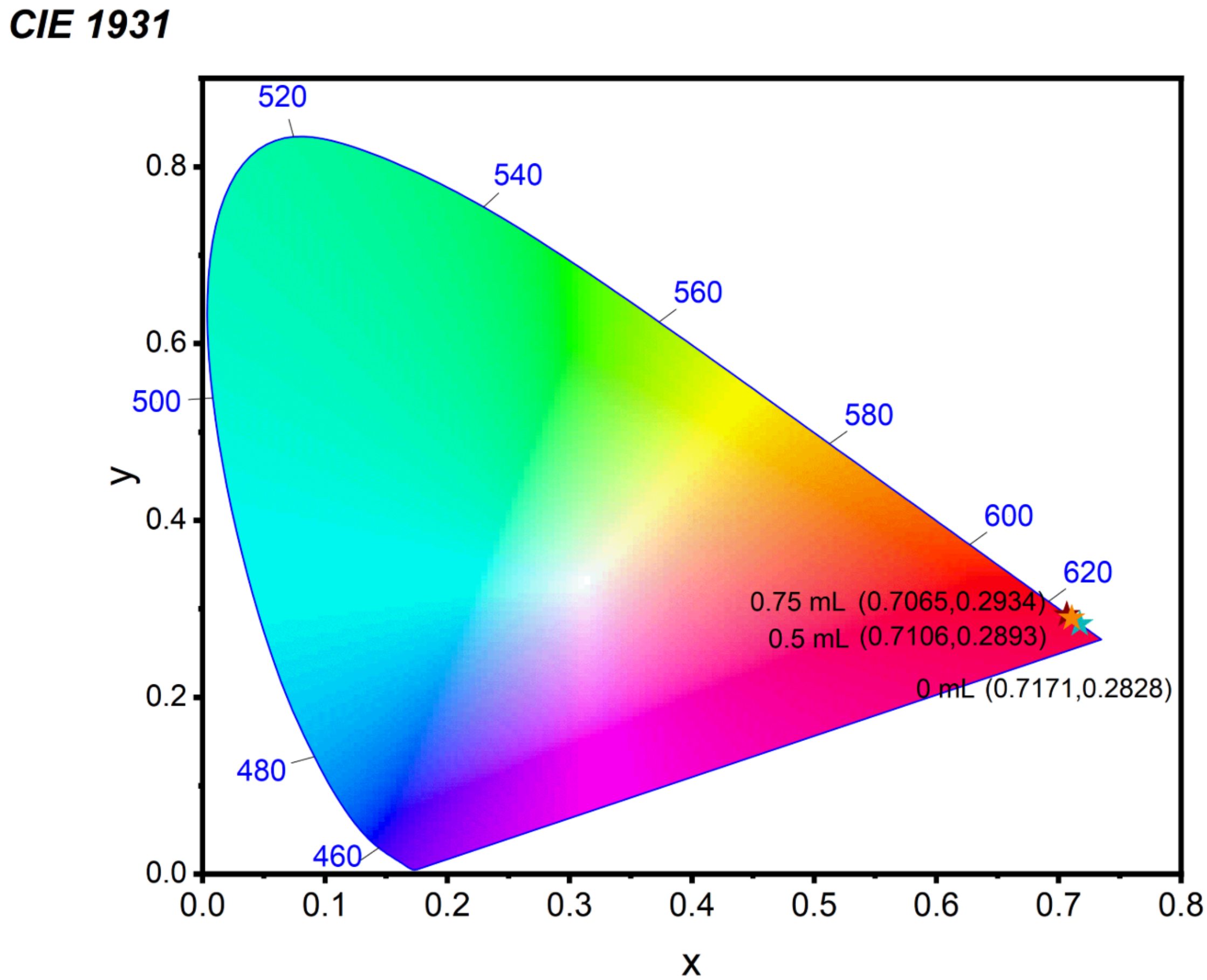

Figure S32: The Corresponding Commission Internationale de l'Éclairage (CIE) coordinates for 0 mL, 0.5 mL and 0.75 mL TDP-I-treated $CsPbI_3$ NCs devices.

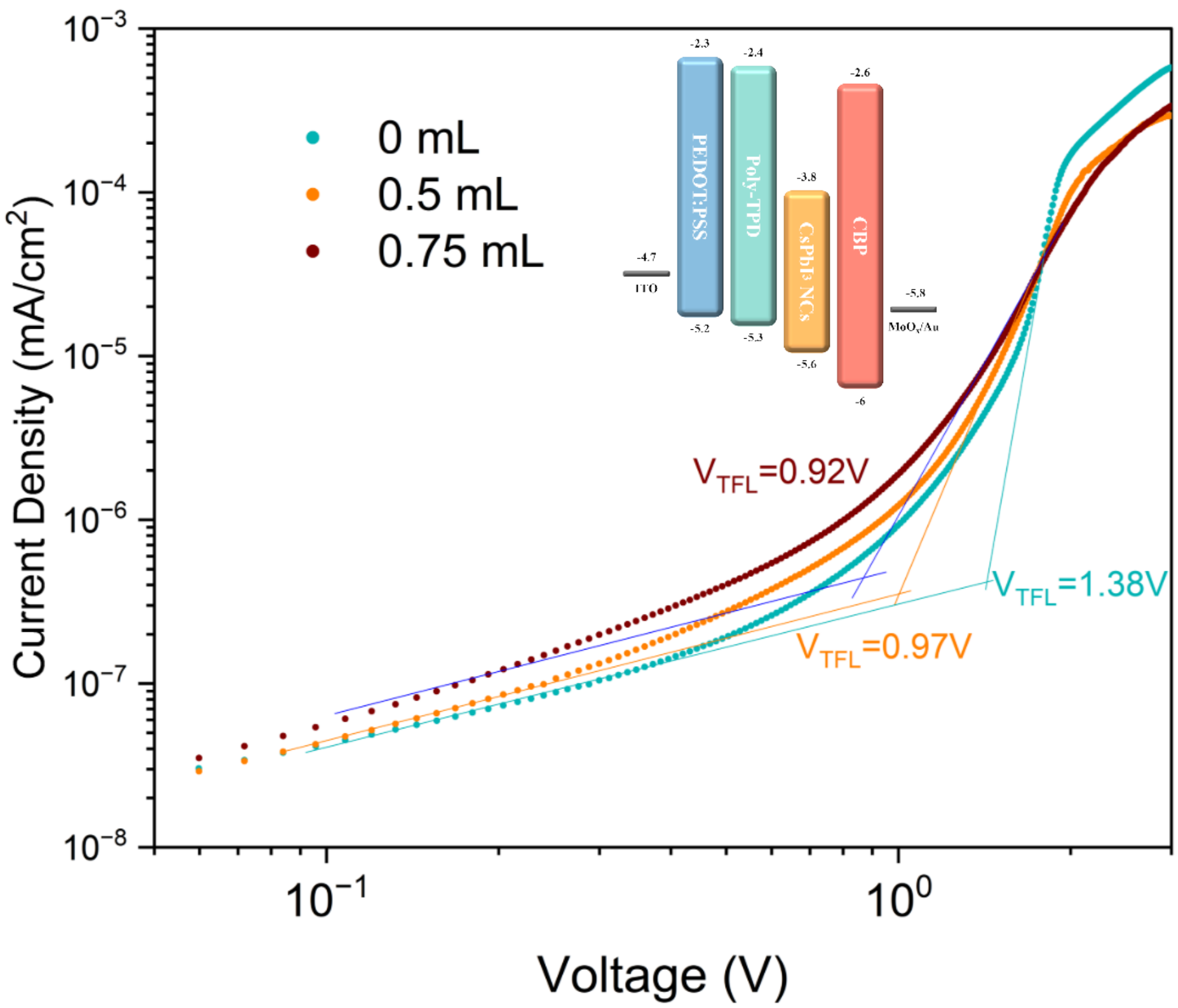

Figure S33: Space charge limit current (SCLC) measurements of 0 mL, 0.5 mL and 0.75 mL TDP-I-treated $CsPbI_3$ NCs based LEDs. Device structure and thickness of each layer: ITO/PEDOT: PSS (30 nm)/poly-TPD (30 nm)/$CsPbI_3$ NCs (150 nm) /CBP (15 nm) /MoO_x (5 nm)/ Au (100nm).

Note: We used the space-charge limit current (SCLC) method to study the trap state densities. We fabricated hole-only devices. The result indicates that the TDP-I-treated $CsPbI_3$ NCs films have lower trap-filled limit voltage (V_{TFL}). We calculated the trap state densities of pristine and exchanged NCs using the following equation:

$$N_{trap} = \frac{2\varepsilon_r\varepsilon_0}{eL^2}V_{TFL}$$

ε_r is the relative permittivity of perovskite (around 6.32 for $CsPbI_3$), ε_0 is the vacuum permittivity, *e* is the elemental charge, *L* is the thickness of the perovskite film (150nm). The results showed that TDP-I- treated $CsPbI_3$ NCs have fewer trap state densities in the hole-only (4.28×10^{16}, 3.01×10^{16}, and 2.86×10^{16} cm^{-3} for pristine, 0.5 mL TDP-I treated, and 0.75 mL TDP-I treated $CsPbI_3$ NCs samples) devices. In brief, the calculated traps decreased by about half after TDP-I treatment.

Table S11: LED performance of 0 mL, 0.5 mL and 0.75 mL TDP-I-treated $CsPbI_3$ NCs.

	EL (nm)	Turn-on Voltage (V)	Maximum EQE (%)	Maximum Luminance (cd/m^2)
0 mL	679	3.26	4.97	30

0.5 mL	672	3.06	10.50	438
0.75 mL	669	2.96	12.40	760

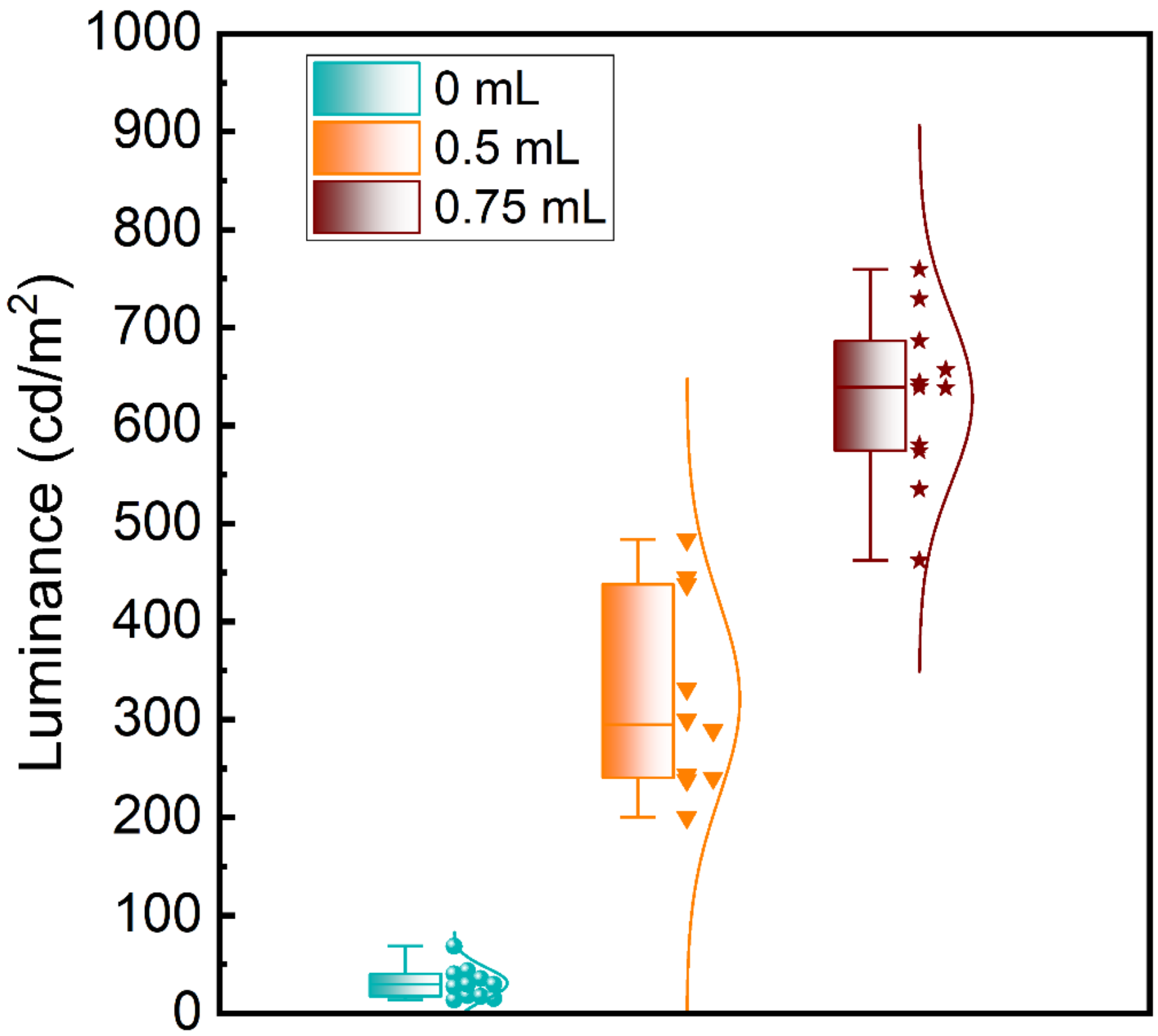

Figure S34: Statistics of Luminance of 0 mL, 0.5 mL and 0.75 mL TDP-I-treated $CsPbI_3$ NCs based LEDs.

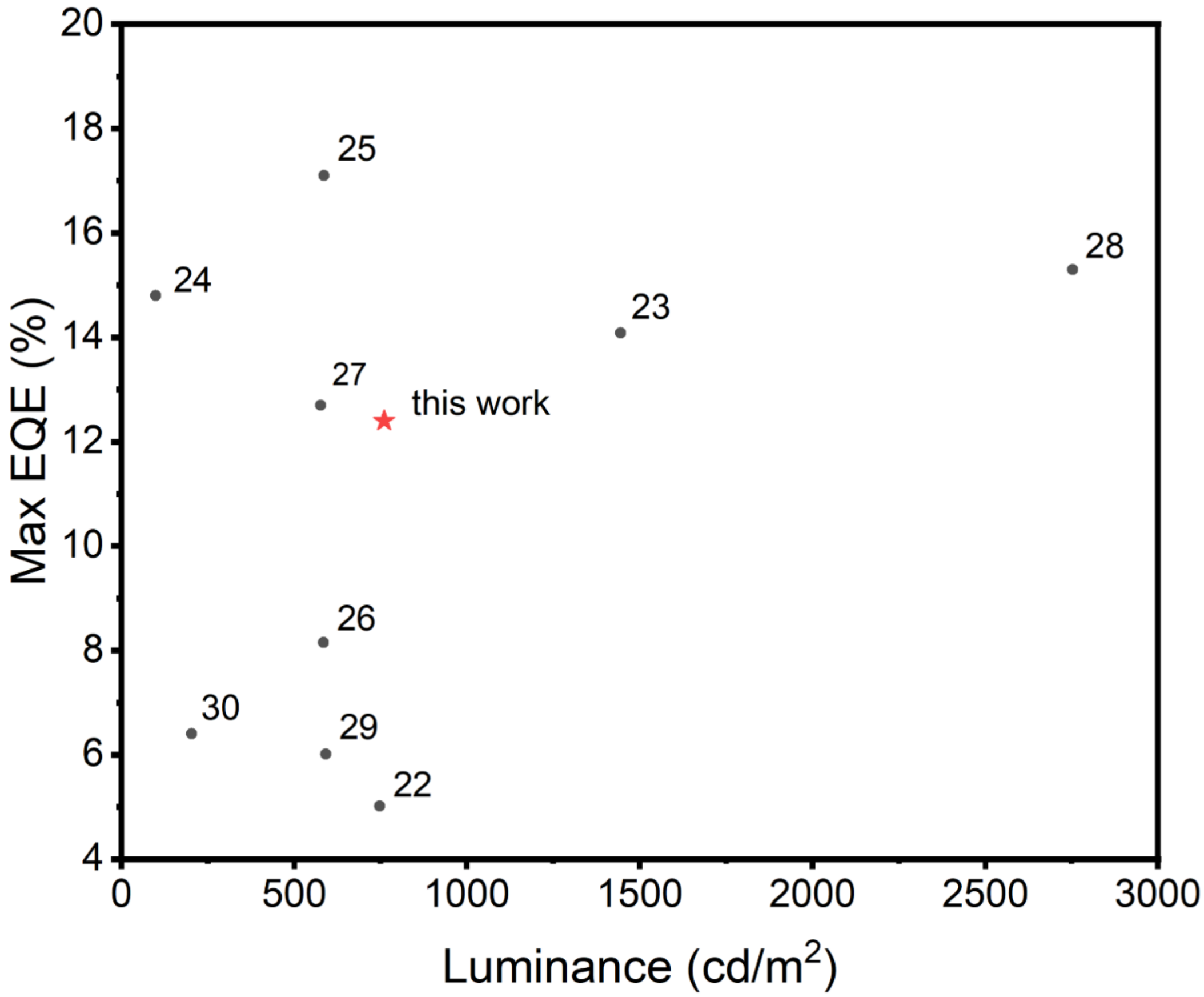

Figure S35: Comparison with $CsPbI_3$ NCs based LEDs presented in the literature using same device structure of the present work. (The numbers denote the corresponding references.)

Table S12. List of various $CsPbI_3$ NCs-based LEDs.

Device Configuration	EL (nm)	EQE (%)	Luminance (cd/m²)	Year	References
ITO/PEDOT: PSS/Poly-TPD/$CsPbI_3$ QDs/TPBi/LiF/Al	688	5.02	748	2018	Bidentate Ligand-Passivated $CsPbI_3$ Perovskite Nanocrystals for Stable Near-Unity Photoluminescence Quantum Yield and Efficient Red Light-Emitting Diodes.[22]
ITO/PEDOT: PSS/Poly-TPD/$CsPbI_3$ QDs/TPBi/LiF/Al	694	14.08	1444	2018	Surface Ligand Engineering for Near-Unity Quantum Yield Inorganic Halide Perovskite QDs and

					High-Performance QLEDs.[23]
ITO/PEDOT: PSS/Poly-TPD/$CsPbI_3$ NCs/TPBi/LiF/Al	692	14.8	100	2020	Efficient All-Inorganic Perovskite Light-Emitting Diodes with Improved Operation Stability.[24]
ITO/PEDOT: PSS/Poly-TPD/$CsPbI_3$ NCs/TPBi/LiF/Al	687	17.1	586	2021	Highly stable $CsPbI_3$:Sr^{2+} nanocrystals with near-unity quantum yield enabling perovskite light-emitting diodes with an external quantum efficiency of 17.1%.[25]
ITO/PEDOT: PSS/Poly-TPD/$CsPbI_3$ NCs/TPBi/LiF/Al	683	8.15	584.5	2022	Synergistic effect of multidentate ligands on $CsPbI_3$ perovskite nanocrystals surface for high efficiency deep red light-emitting diode.[26]
ITO/PEDOT: PSS/Poly-TPD/KI/$CsPbI_3$ NCs/TPBi/LiF/Al	687	12.7	576	2022	Passivation Layer of Potassium Iodide Yielding High Efficiency and Stable Deep Red Perovskite Light-Emitting Diodes.[27]
ITO/PEDOT: PSS/Poly-TPD/$CsPbI_3$ NCs/TPBi/LiF/Al	680	15.3	2753	2022	Enhancing the Efficiency and Stability of $CsPbI_3$ Nanocrystal-Based Light-Emitting Diodes through Ligand Engineering with Octylamine.[28]
ITO/PEDOT: PSS/Poly-TPD/$CsPbI_3$ NCs/TPBi/LiF/Al	690	6.01	592	2023	Highly stable lanthanide-doped $CsPbI_3$ perovskite nanocrystals with near-unity quantum yield for efficient red light-emitting diodes.[29]
ITO/PEDOT: PSS/Poly-TPD/$CsPbI_3$ NCs/TPBi/LiF/Al	675	6.4	204	2024	Room-Temperature Large-Scale Synthesis of

					Black-Phase $CsPbI_3$ Nanocrystals for Red Light-Emitting Diodes.[30]
ITO/PEDOT: PSS/Poly-TPD/$CsPbI_3$ NCs/TPBi/LiF/Al	670	12.4	760	2026	This work

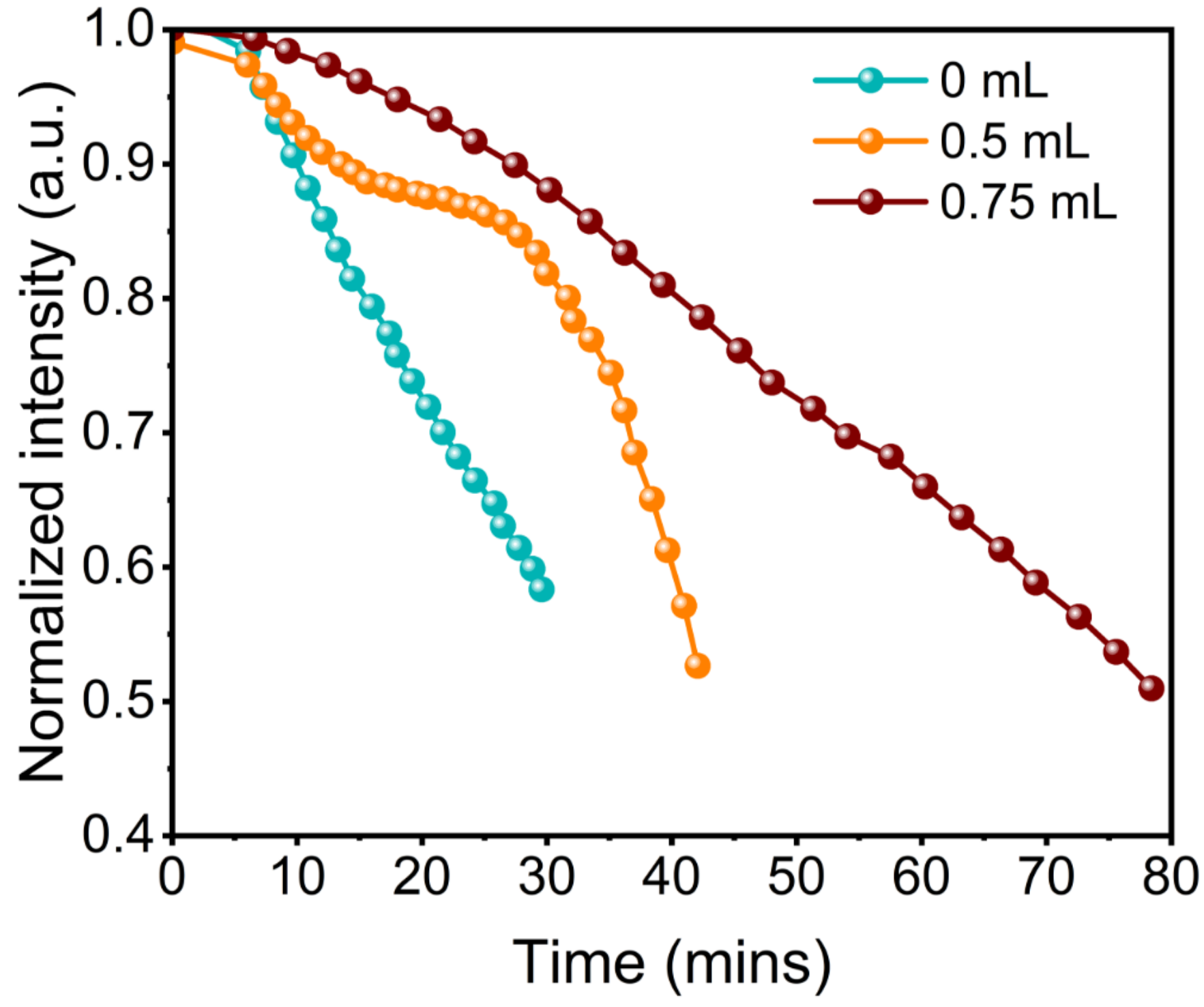

Figure S36: Operational stability of 0 mL, 0.5 mL and 0.75 mL TDP-I-treated $CsPbI_3$ NCs based LEDs evaluated under a constant current density of 1 mA cm^{-2}.

References:

1. Gerhard Wider, Lars Dreier, Measuring Protein Concentrations by NMR Spectroscopy. J. Am. Chem. Soc. 2006, 128, 8, 2571–2576.

2. Van Beek, B. Auto-FOX. Preprint at https://doi.org/10.5281/zenodo.7919377 (2023).

3. Cosseddu, S. & Infante, I. Force Field Parametrization of Colloidal CdSe Nanocrystals Using an Adaptive Rate Monte Carlo Optimization Algorithm. J. Chem. Theory Comput. 13, 297–308 (2017).

4. Marrink, S. J., Risselada, H. J., Yefimov, S., Tieleman, D. P. & de Vries, A. H. The MARTINI Force Field: Coarse Grained Model for Biomolecular Simulations. J. Phys. Chem. B 111, 7812–7824 (2007).

5. Van Beek, B., Zito, J., Visscher, L. & Infante, I. CAT: A Compound Attachment Tool for the Construction of Composite Chemical Compounds. J. Chem. Inf. Model. 62, 5525–5535 (2022).

6. Martínez, L., Andrade, R., Birgin, E. G. & Martínez, J. M. PACKMOL: a package for building initial configurations for molecular dynamics simulations. J. Comput. Chem. 30, 2157–2164 (2009).

7. Van Der Spoel, D. et al. GROMACS: Fast, flexible, and free. J. Comput. Chem. 26, 1701–1718 (2005).

8. GROMACS Documentation Release 2023.1 GROMACS Development Team. (2023).

9. Abraham, M. et al. GROMACS 2023.1 Manual. Preprint at https://doi.org/10.5281/zenodo.7852189 (2023).

10. Abraham, M. et al. GROMACS 2023.1 Manual. Preprint at https://doi.org/10.5281/zenodo.7852189 (2023).

11. Berendsen, H. J. C., van der Spoel, D. & van Drunen, R. GROMACS: A message-passing parallel molecular dynamics implementation. Comput. Phys. Commun. 91, 43–56 (1995).

12. Abraham, M. J. et al. GROMACS: High performance molecular simulations through multi-level parallelism from laptops to supercomputers. SoftwareX 1–2, 19–25 (2015).

13. Páll, S., Abraham, M. J., Kutzner, C., Hess, B. & Lindahl, E. Tackling Exascale Software Challenges in Molecular Dynamics Simulations with GROMACS. in 3–27 (2015). doi:10.1007/978-3-319-15976-8_1.

14. Lindahl, E., Hess, B. & van der Spoel, D. GROMACS 3.0: a package for molecular simulation and trajectory analysis. J. Mol. Model. 7, 306–317 (2001).

15. Hess, B., Kutzner, C., van der Spoel, D. & Lindahl, E. GROMACS 4: Algorithms for Highly Efficient, Load-Balanced, and Scalable Molecular Simulation. J. Chem. Theory Comput. 4, 435–447 (2008).

16. Pronk, S. et al. GROMACS 4.5: a high-throughput and highly parallel open source molecular simulation toolkit. Bioinformatics 29, 845–854 (2013).

17. Bussi, G., Donadio, D. & Parrinello, M. Canonical sampling through velocity rescaling. J. Chem. Phys. 126, (2007).

18. Berendsen, H. J. C., Postma, J. P. M., Van Gunsteren, W. F., Dinola, A. & Haak, J. R. Molecular dynamics with coupling to an external bath. J. Chem. Phys. 81, 3684–3690 (1984).

19. Parrinello, M. & Rahman, A. Polymorphic transitions in single crystals: A new molecular dynamics method. J. Appl. Phys. 52, 7182–7190 (1981).

20. Hess, B., Bekker, H., Berendsen, H. J. C. & Fraaije, J. G. E. M. LINCS: A Linear Constraint Solver for molecular simulations. J. Comput. Chem. 18, 1463–1472 (1997).

21. Essmann, U. et al. A smooth particle mesh Ewald method. J. Chem. Phys. 103, 8577–8593 (1995).

22. Jun Pan, Yuequn Shang, Jun Yin, Zhijun Ning*, Osman M. Bakr*. Bidentate Ligand-Passivated $CsPbI_3$ Perovskite Nanocrystals for Stable Near-Unity Photoluminescence Quantum Yield and Efficient Red Light-Emitting Diodes. J. Am. Chem. Soc. 2018, 140, 2, 562–565.

23. Guopeng Li, Jingsheng Huang, Hanwen Zhu, Yanqing Li*, Jian-Xin Tang*, Yang Jiang*. Surface Ligand Engineering for Near-Unity Quantum Yield Inorganic Halide Perovskite QDs and High-Performance QLEDs. Chem. Mater. 2018, 30, 17, 6099–6107.

24. Guoqing Cheng, Yan Liu, Tao Chen, Tongfei Shi*, Zhengguo Xiao*. Efficient All-Inorganic Perovskite Light-Emitting Diodes with Improved Operation Stability. ACS Appl. Mater. Interfaces 2020, 12, 15, 18084–18090.

25. Cheng Chen, Tongtong Xuan, Wenhao Bai, Le Wang, Rong-Jun Xie. Highly stable $CsPbI_3:Sr^{2+}$ nanocrystals with near-unity quantum yield enabling perovskite light-emitting diodes with an external quantum efficiency of 17.1%. Nano Energy, Volume 85, 2021, 106033, ISSN 2211-2855.

26. Qingyuan Qiao, Zhaohui Shen, Na Jiang, Suling Zhao, Zheng Xu. Synergistic effect of multidentate ligands on $CsPbI_3$ perovskite nanocrystals surface for high efficiency deep red light-emitting diode. Organic Electronics, Volume 107, 2022, 106550, ISSN 1566-1199.

27. Cheng Chen, Tongtong Xuan*, Yang Yang, Le Wang*, Rong-Jun Xie*. Passivation Layer of Potassium Iodide Yielding High Efficiency and Stable Deep Red Perovskite Light-Emitting Diodes. ACS Appl. Mater. Interfaces 2022, 14, 14, 16404–16412.

28. Hanming Li, Wei Dong, Xinyu Shen, Xiaoyu Zhang*, Qingfeng Dong*. Enhancing the Efficiency and Stability of $CsPbI_3$ Nanocrystal-Based Light-Emitting Diodes through Ligand Engineering with Octylamine. J. Phys. Chem. C 2022, 126, 2, 1085–1093.

29. Wenlin Xia, Zhenwei Ren, Zhishuai Zheng, Chengzhao Luo, Jie Li, Wenchen Ma, Xin Zhou, Yu Chen; Highly stable lanthanide-doped $CsPbI_3$ perovskite nanocrystals with near-unity quantum yield for efficient red light-emitting diodes. Nanoscale 2023; 15 (3): 1109–1118.

30. Ju-Hyun Yoo, Kyung Yeon Jang, Jihye Jang, Cheolmin Park, Tae-Woo Lee, Jin-Woo Park*. Room-Temperature Large-Scale Synthesis of Black-Phase $CsPbI_3$ Nanocrystals for Red Light-Emitting Diodes. ACS Appl. Nano Mater. 2024, 7, 24, 28119–28130.
